# Violence against children in Latin America and Caribbean countries: a comprehensive review of national health sector efforts in prevention and response

**DOI:** 10.1186/s12889-016-3562-3

**Published:** 2016-09-22

**Authors:** Andrea L. Wirtz, Carmen Alvarez, Alessandra C. Guedes, Luisa Brumana, Cecilie Modvar, Nancy Glass

**Affiliations:** 1 Department of Epidemiology, Center for Public Health and Human Rights, Johns Hopkins Bloomberg School of Public Health, Baltimore, USA; 2 Community Public Health Nursing, Johns Hopkins School of Nursing, Baltimore, USA; 3 Family, Gender and Life Course Department, Pan American Health Organization/World Health Organization, Regional Office for the Americas, Washington, DC USA; 4 UNICEF Latin America and Caribbean Regional Office, Panama, Panama

**Keywords:** Violence against children, Health system, Latin America, Caribbean

## Abstract

**Background:**

Violence against children (VAC) remains a global problem. The health sector has an opportunity and responsibility to be part of the multi-sector collaboration to prevent and respond to VAC. This review aimed to assess the health sector’s response to VAC among Latin American & Caribbean (LAC) countries, particularly as it relates to physical violence, sexual violence, and neglect.

**Method:**

National protocols for the identification and provision of health care to child survivors of violence, abuse and neglect were solicited in partnership with UNICEF and PAHO/WHO country offices within the LAC region. A parallel systematic review was undertaken in January 2015 to review studies published in the last 10 years that describe the regional health sector response to VAC.

**Results:**

We obtained health sectors guidelines/protocols related to VAC from 22 of 43 (51 %) countries and reviewed 97 published articles/reports that met the review inclusion criteria. Country protocols were presented in Spanish (*n* = 12), Portuguese (*n* = 1), and English (*n* = 9). Thematic areas of country protocols included: 1) identifying signs and symptoms of VAC, 2) providing patient-centered care to the victim, and 3) immediate treatment of injuries related to VAC. The systematic review revealed that health professionals are often unaware of national protocols and lack training, resources, and support to respond to cases of VAC. Further, there is limited coordination between health and social protection services.

**Conclusions:**

VAC remains an international, public health priority. Health professionals are well-positioned to identify, treat and refer cases of VAC to appropriate institutions and community-based partners. However, poor protocol dissemination and training, limited infrastructure, and inadequate human resources challenge adherence to VAC guidelines.

**Electronic supplementary material:**

The online version of this article (doi:10.1186/s12889-016-3562-3) contains supplementary material, which is available to authorized users.

## Background

Violence against children (VAC) is a human rights violation and global public health problem. Negative and lifelong effects are associated with VAC, including impacts on physical, mental, and reproductive health as well as social and cognitive development [1]. VAC is defined as any physical or sexual abuse, or neglect; which are often categorized as “child abuse”, “child maltreatment” and other variations of these terms in the published literature. Further, VAC includes all forms of physical and sexual violence and emotional abuse, neglect, negligent treatment and exploitation that is perpetrated against minors aged 18 years and under [2, 3]. VAC is also associated with other forms of violence in the household, such as intimate partner violence and intergenerational cycles of violence [4]. Further, VAC has negative impacts on educational attainment of survivors, as well as labor and productivity when survivors reach adulthood [5]. At the societal level, VAC has multiple costs that impact development. These include direct costs, such as the medical costs of treatment and visits to health care providers; indirect costs, such as lost productivity, disability, decreased quality of life, and premature death; and costs borne by the justice system and institutions, including costs for social welfare and foster care, expenditures associated with apprehending and prosecuting offenders, and costs to the educational system [1].

Every year, over half of the world’s children are victims of physical, sexual, and emotional violence [6]. Prevalence estimates of recent violence (past 12 months) among children have suggested that over 30 % of children in Latin America experienced recent violence [7]. However, in the LAC region, population-based studies are heterogeneous in methodology and definitions challenging any comparisons of estimates calculated for childhood physical and sexual violence across settings and countries. In El Salvador, a nationally representative study reported that 42 % of women and 62 % of men reported physical violence before age 15 years [8]. In Guatemala and Honduras, 5 % and 8 %, respectively, of adult women reported experiencing sexual violence during childhood [8]. Given the diversity of definitions used across studies and settings, the stigma associated with being identified as a VAC victim, as well as the regulatory and ethical issues associated with conducting surveys/interviews with children on their experiences of VAC [9], existing estimates of prevalence in VAC is likely an underestimate of the true prevalence in LAC.

Within this context, national health protocols are instrumental in enhancing multi-sectoral collaboration and guiding the different sectors in responding to children in need of protection, treatment, care and referral. Healthcare professionals are in a unique position to raise awareness of VAC as a public health issue and to promote both prevention and response of VAC within and outside of healthcare settings [9]. Health professionals have frequent contact with parents/caregivers and children, such as during routine visits for required vaccinations or school physical exams and/or for common childhood illnesses as well as when children seek services in the emergency department for an injury. Health professionals should not only be able to identify children in dangerous situations when there are visible injuries, but also have the skills to identify a child survivor even when the patient’s chief complaint is seemingly unrelated to violence, abuse or neglect [9].

The Pan American Health Organization/World Health Organization Regional Office for the Americas (PAHO/WHO) and UNICEF Latin America and the Caribbean Regional Office (LACRO) has VAC prevention and response as a major focus of their programs. As such, PAHO/WHO and UNICEF are collaborating to partner with governments to develop, implement and monitor evidence-based health sector protocols to provide guidance to health care professionals in meeting their critical role in VAC prevention and response. All countries in LAC have ratified the UN Convention on the Rights of the Child (CRC), and in recent years, Argentina, Bolivia, Brazil, Costa Rica, Honduras, Nicaragua, Peru, Uruguay, and Venezuela have made important steps to address VAC through the full legal prohibition of corporal punishment [10]. Several countries, such as El Salvador, Guatemala, Honduras, and Nicaragua, also supported development of or revisions to national protocols or guidelines intended to guide the health sector’s response to VAC [11]. In LAC, child protection falls under the mandate of what is generally referred to as Comprehensive National Child Protection Systems (Sistemas Nacionales de Protección Integral) [12]. Though diverse, these systems generally intend to coordinate the relations among all government and non-governmental agencies who comprise the Child Protection Systems in order to ensure children are protected against or provided with appropriate care following violence, abuse, or neglect. It is unknown the extent to which national protocols have been developed in LAC to guide response by health professionals to VAC and how protocols have impacted the identification, care, treatment and referrals of children from the health sector to appropriate protections services.

The overall purpose of this study is to collaboratively assess the health sector’s response to VAC within the LAC region, by reviewing both national health sectors protocols for VAC and published research articles, particularly those discussing health sector response to physical violence, sexual violence, and neglect, in order to provide guidance and recommendations to strengthen national health sector response to VAC.

## Methods

To achieve this purpose, we conducted a comprehensive review, which included two different data sources and parallel activities: 1) collection and review of existing LAC national protocols for the identification and provision of health care to child survivors of violence, abuse and neglect; and 2) systematic review of published articles and reports to examine current types of response, training, interventions, and collaborations between the child protection and health sectors to address VAC.

### Review of clinical protocols

In collaboration with PAHO and UNICEF, the team solicited country-level health sector protocols using email requests disseminated by PAHO/UNICEF colleagues to focal points in each of the 43 LAC country offices. Email recipients were informed about the purpose of the project and asked to collaborate by providing the project team with an electronic copy of the most recent national VAC protocol for the health sector response. Two reminder emails were subsequently sent to country focal points over a two-month period to follow-up with the request if the national protocol had not been received.

The review of the health sector protocols focused on identification of guidance offered to health care professionals in support of optimal response to violence, abuse and neglect of children. We adapted an existing review matrix developed to assess protocols addressing violence against women [13] and included the following categories: purpose of the protocol, definition of child physical abuse, definition of child sexual abuse, components of access to care (e.g., service hours), health care professionals and roles referenced, components of services/actions recommended, treatment plans/follow up, and mandatory reporting requirements.

### Systematic review of peer reviewed publications

**Fig. 1:**
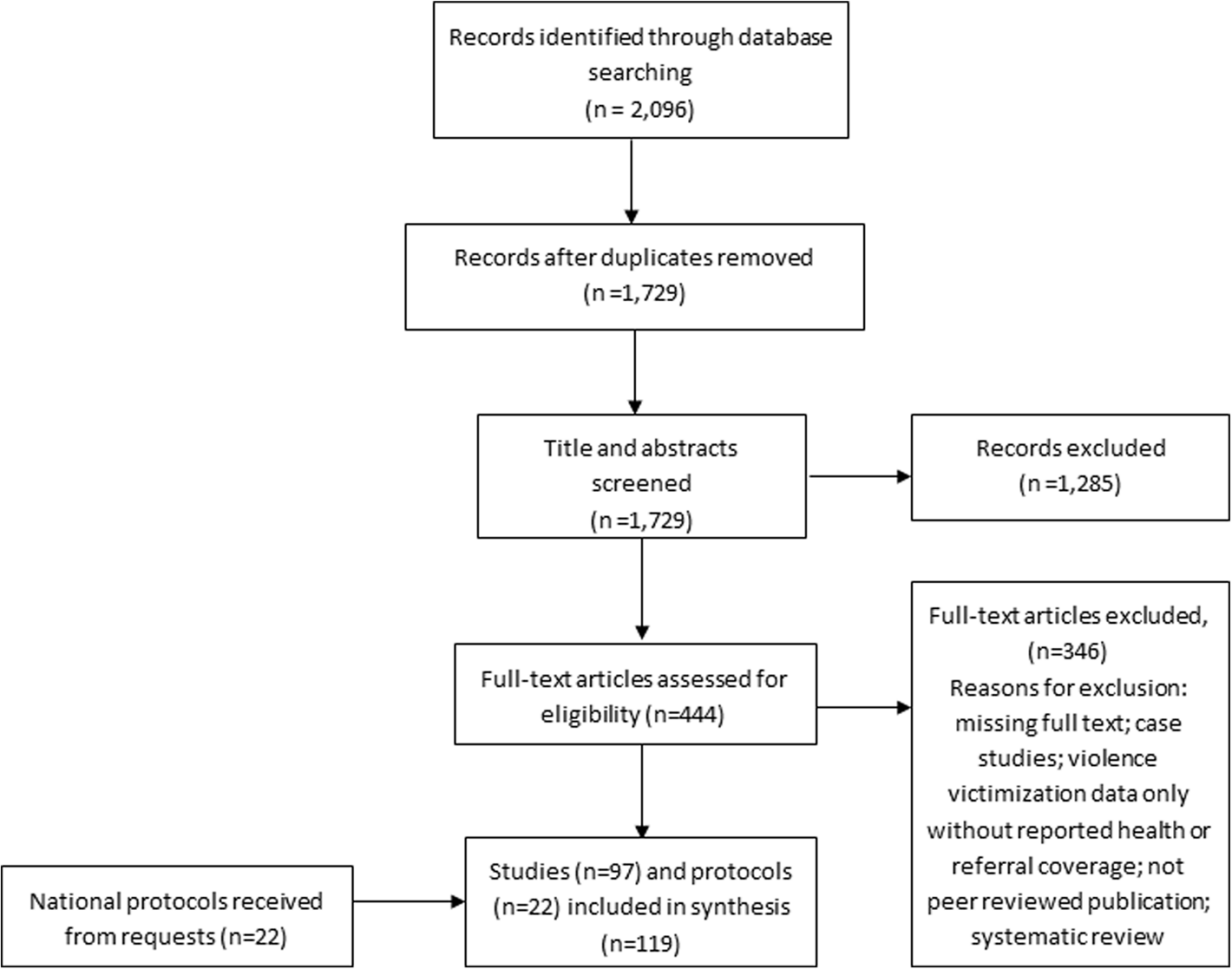
PRISMA flow diagram

The systematic review of peer-reviewed public health, medicine, and nursing literature was conducted to map the reported health sector response, guiding curriculum, interagency collaboration, as well as strengths and weaknesses of the current health sector response to VAC in the LAC region. Searched databases included Pubmed, Embase, PsycInfo, CINAHL, and LILACS and the review was conducted following PRISMA guidelines [14]. The first four databases index global publications from scientific, health, and nursing sources. PAHO and WHO jointly developed the LILACS database to index scientific and technical literature from the LAC region (http://lilacs.bvsalud.org/en/). A public health informationist collaborated with the authors to develop search terms and conduct the database searches. Databases were searched between the dates of 27 January 2005 and 26 January 2015. Searches followed controlled vocabulary and keywords, which were used in combination for the concepts of child abuse, health sector response, and LAC countries with no restriction on language of the publication/report. Index specific search terms are provided in the Additional file 1. Identified publications were compiled in an Endnote reference manager (Thomson Reuters, Version 7) and duplicates were removed. A title and abstract review was used to identify eligible literature and was followed by a full text review and data extraction of eligible publications. Reviews and data extraction of English and Spanish articles were conducted by two of the authors (AW and CA). Two research assistants who were fluent in Portuguese (native speakers from Brazil) reviewed and extracted relevant data from literature published in Portuguese language. All data extraction was documented in English; while no formal translation process was used, selected studies were discussed amongst the authors and research assistants to verify content and applicability to the study purpose. Figure 1 provides the PRISMA flow diagram.

Peer reviewed publications were included if they met the following criteria: participants included children (up to age 18) and/or health care professionals (physicians, social workers, nurses, or health administrators); study population was in a LAC country; study addressed health sector activities, training, and/or policies regarding child (up to age 18) violence, abuse, neglect and protection; or were intervention studies with VAC related outcomes; and were original research articles or reports that addressed the above criteria. Publications were excluded if they focused exclusively on prevalence or sequelae of VAC without reporting any health service or other service provided; focused on domestic violence/abuse of adults but did not include children; did not include full texts (were abstracts only); or if the study population was not in LAC countries – e.g., child abuse among Latino populations in the U.S. As noted above, the purpose of the systematic review was to examine the current types of health sector response for VAC, evaluating the quality of the studies presented in the publications was not a component of this review.

To identify the health sector response and linkages to other relevant sectors, such as child protection, criminal justice, legal system, we developed data extraction forms pertaining to three domains: 1) health services and linkages to other related services; 2) training/curriculum/ health professional response to VAC; and, 3) health sector based interventions to prevent or respond to VAC. Additional information related to the study design (location, year of study, design, sample size, population) were also included for data extraction. Table 1 displays the types of data that were extracted from the published literature for each domain. Trained research assistants and co-authors fluent in Portuguese or Spanish independently extracted data from identified articles. All extracted data were reviewed for accuracy by one of the first authors (AW).

**Table 1:** Key domains and data extracted from peer reviewed literature in the LAC region

Domain	Health services and linkage	Training/curriculum	Interventions
Categories:	Study (Author, year pub),	Study (Author, year pub),	Study (Author, year pub),
	Country,	Country,	Country,
	Year(s) of study,	Year(s) of study,	Year(s) of study,
	Study design, sample size (N), and target population	Targeted profession/ degree for training	Study design, sample size (N), and target population
	Accessibility of services,	Training content	Intervention description
	Types of services offered,	Duration of training	Identified gaps/limitations
	Linkage to other services,	Method of evaluation	Results/Impact
	Follow-up plans	Identified gaps/limitations.	
	Identified gaps/limitations.	Results/Impact	
	Results/Impact		

## Results

### National health sector protocol review

We received health sector protocols from 22 out of 43 countries contacted (51 % response rate). The 22 countries that provided protocols include: Anguilla, Antigua & Barbuda, Argentina, Barbados, Bolivia, Brazil, British Virgin Islands, Chile, Colombia, Dominica, Dominican Republic, El Salvador, Grenada, Guatemala, Guyana, Nicaragua, Paraguay, Peru, St. Kitts & Nevis, Trinidad & Tobago, Uruguay, and Venezuela. Protocols were presented in Spanish (*n* = 12), Portuguese (*n* = 1), and English (*n* = 9). Dates of publication of the national protocols ranged from 2001 – 2014; with 5 national protocols not providing dates of publication [15–19]. Additional file 1: Appendix 2 displays brief descriptions of each of the 22 national protocols received and reviewed.

### National protocol objectives

The objectives and target audience for the protocols informed the recommendations provided in the protocols. The main objectives for the protocols varied from broad guidelines, such as presenting guidance for how to respond to domestic violence, to more specific guidelines related to protection of at-risk children and standards of care and treatment, as well as for reporting and investigating cases of VAC. Protocols intended for a broad audience – police, teachers, and health care professionals– included general guidance and focus on details for VAC documentation. Over half of the received protocols (*n* = 13, 59 %) [15, 19–32] targeted health care professionals as the sole intended audience. The health care professionals referenced in the protocols with specific roles and responsibilities in cases of VAC included physicians, nurses, social workers, and psychologists. These health sector specific protocols included details about patient care, treatment plans, and patient follow-up.

### Identification of VAC

Protocol guidance for identification of VAC focused on three main components: the definition of VAC, description of risk factors utilized to recognize potential cases of VAC, and description of signs and symptoms of VAC.

Case definitions are critical to guiding a health professional identification and response to VAC. Several protocols provided extensive details on the definitions of different forms of VAC, such as physical violence, psychological violence, neglect, abandonment, child labor, and sexual abuse, while others provided no definition. Specifically, eight (36.4 %) [15–17, 21, 29, 33–36] of the country protocols did not include definitions for child abuse, including physical violence, and two of these country protocols failed to provide definitions for sexual violence. For those that did provide a definition (*n* = 15, 68.2 %) [18–20, 22, 26–28, 37–47], all but one included the concept of a power differential between the perpetrator and victim in the definition. Age limits were included in several definitions, as exemplified in the protocol from Colombia: *“Sexual abuse is a crime when sexual intercourse occurs with someone under the age of 14 - any sexual act with someone under the age of 14 is a criminal act”* [24].

The majority (*n* = 13, 59 %) of protocols described risk factors for VAC [17, 19, 20, 24, 26, 27, 30, 31, 37–39, 42, 45, 48, 49]. Protocols in which risk factors were addressed primarily focused on low socio-economic status and substance abuse by parents/caregivers. Nine protocols (41 %) identified domestic violence or intimate partner violence as a risk factor for VAC [17, 19, 20, 29, 31, 38, 42, 45, 48].

Although two-thirds (*n* = 15; 68.2 %) of reviewed country protocols provided definitions of VAC, almost all protocols included extensive lists of signs and symptoms indicative of various types of violence, abuse and neglect. These lists included both physical and behavioral indicators. Physical signs and symptoms included facial injuries on infants, and bruising with a pattern (e.g., belt and buckle marks). Behavioral indicators of VAC often included nighttime bedwetting; inflicted self-harm; and being sexually expressive beyond what is age appropriate. Two protocols (Dominican Republic and Guatemala) [27, 48] instructed service providers to suspect VAC when two or more signs/symptoms are present. However, the evidence and effectiveness of this strategy for identifying VAC was not referenced. In addition to recognizing injuries and behavioral indicators, guidance recommended that health professionals be alert to injuries with inconsistent explanation by parent and/or child as to how the injury occurred.

### Response to identified or suspected VAC

Consistent among the protocols, particularly those targeted to health care professionals, were the components of history taking, physical assessment, provision of necessary treatment, and follow-up care. Methods to interview the child survivor were the most consistently emphasized component in this process. The majority of protocols provided details on where and how to best interview the child: “*Make sure the child is comfortable, avoid interrogation, avoid being judgmental, use open ended questions…information about the injuries or findings on assessment should also be asked to the parents/guardians”* [42]. One protocol (Venezuela) recommended considering the possibility of alcohol intoxication by the child or other psychological problem if the child/adolescent could not recall details surrounding the alleged abuse [35].

Only about one-third of protocols (*n* = 7; 31.8 %) [19, 20, 22, 24, 26, 29, 31] specified who should conduct the interview with the identified/suspected child survivor. Five (23 %) country protocols specified that once VAC was suspected, personnel from a child protection or social welfare agency were responsible for interviewing the child or were required to be present while a health care professional interviewed the child [19, 24, 27, 40, 48]. Two protocols (Grenada and St. Kitts & Nevis) underscored the importance of investigative personnel from child protection or social welfare present during the interview, in order to avoid having the child survivor tell his/her story multiple times [19, 40].

Two-thirds of submitted country protocols (*n* = 15; 68.2 %) [16–18, 20–22, 24, 27, 28, 34, 35, 38, 42, 44, 45] did not specify which type of health care professional (nurse or physician) should conduct the child’s physical assessment. However, six protocols (27.3 %) [15, 19, 29, 31, 43, 47] noted that a physician or nurse should conduct the exam, while five protocols (22.7 %) [15, 17, 20, 26, 40] indicated that “qualified” or trained professionals should conduct the physical assessment. Instructions for the physical assessment emphasized documentation of injuries and collection of forensic evidence; however, no definitions on what constituted “forensic evidence” were provided in the protocols.

Treatment recommendations focused on treatment of injuries, diagnostic tests for sexually transmitted infections (STI), post-exposure prophylactic treatment for HIV, and emergency contraception. The Colombia national protocol included the possibility of an abortion for child survivors of sexual violence; however, it was unclear whether parental/guardian consent was required for an abortion in addition to child consent [24]. Guidance in protocols submitted by eight countries (36.4 %) did not extend beyond prophylactics and treatment of injuries.

Three country protocols (13.6 %) [27, 44, 48] included treatment plans with recommendations that were based on the severity of injuries or type of VAC. For example, the Dominican Republic protocol indicated that mild abuse, defined as no threat to life or physical injury required family counseling; severe abuse, defined as threat to life required inpatient care and contact with social services to explore housing options [25]. However, the protocol did not provide guidance on the assessment method to determine mild to severe VAC.

Procedures for psychological treatment and follow-up care for child survivors were inconsistent across the submitted protocols. Almost all protocols emphasized the importance of offering mental health services and psychological treatment to survivors; however, the methods for implementing this recommendation were not described. Half of submitted protocols (*n* = 11; 50.0 %) [17, 22, 24, 26, 27, 31, 38, 40, 42, 45, 48] provided guidance on follow-up care. Options for follow-up care provided in the protocols included: home-visits and follow-up clinic visits by the child within a few weeks. The protocols lacked detail on the roles and responsibilities of health care and child protection sectors, especially with regard to follow-up services for the child survivor and family, for example only two country protocols (Chile and Uruguay) designated the safety assessment as the role/responsibility of social workers and psychologists. Four protocols (18.2 %) [16, 26, 31, 42] included recommendations to service providers to evaluate the child’s safety within the home and make a determination of whether the child can safely return or should be placed in the care of a guardian.

Two protocols (Argentina and Colombia) addressed the importance of access to care for survivors. Both of these protocols underscored the importance of providing immediate care to the survivor that is free of charge, available within the survivors area of residence, available 24 h a day / 7 days a week, and confidential [21, 24]. None of the reviewed protocols addressed VAC services in rural settings or other settings with limited resources/services. Further, there was no indication on submitted protocols of what services could be provided in the absence of a “trained” professional, such as referrals to community based programs by community health workers.

### Mandatory reporting

Half of the submitted protocols (*n* = 11; 50.0 %) [17, 19–22, 27, 29, 35, 38, 40, 42] indicated that any professional, particularly health care professionals, that suspected or had confirmed a VAC case should report the case to the local welfare/protection agency or the police. Four country protocols (Anguilla, Barbados, British Virgin Islands, and Grenada; 18.2 %) indicated that failure to report a case within the proposed time frame, often within 24–72 h, was punishable by fine. Five protocols (22.7 %) specified mandatory reporting of *all* forms of suspected VAC [17, 20, 22, 35, 40]. For example, the Anguilla protocol states “…*teacher, employer, medical provider, who has reasonable grounds to believe that the minor is being abused shall report this to a police officer within a reasonable time period. Failure to report may result in 2 years imprisonment and/or $5000.00 fine*” (US$1,851.85) [20]. In six protocols (27.3 %) [17, 20, 22, 29, 35, 40], guidance on mandatory reporting of what was defined as “milder” forms of violence, abuse and neglect was not provided. For example, in the Guatemala protocol, if only one indicator of VAC was noted on exam, the recommendation was to review good parenting practices [27].

Other than guidance to refer cases of suspected VAC to a social or child protection agency, none of the protocols presented a clear system of referral for child survivors. There were, however descriptions on how to notify leadership/superiors in the health care administration of suspected or confirmed VAC. For example, in two protocols (Antigua & Barbuda, and St. Vincent & the Grenadines), multiple steps were to be taken before the suspected VAC case could be reported to child protection or social welfare agency [16, 18]. The protocol from Antigua and Barbuda, for example, stipulates that if a nurse were to learn of child abuse, he/she should provide either written or verbal notification to his/her immediate superior; this report would then move through a chain of command in the health care setting prior to the report being released to the child welfare department (CWD) where it would be formalized by CWD to determine a care plan [18]. Protocols that required multiple steps within the health care setting prior to reporting the incident to authorities did not include a feedback mechanism. In other words, if a nurse initiates the reporting process for suspected child abuse, it is unclear if the nurse is ever informed of the outcome of his/her report.

### Education/dissemination of protocols

Importantly, despite most protocols indicating its intended audience few presented an education or dissemination plan for the protocol. Four protocols (18 %) [15, 22, 29, 47] specifically addressed the need for ongoing training and capacity building for health and service providers. However, processes for dissemination of the protocol to the intended audience was not explicit in the 22 submitted protocols.

### Systematic literature review of health sector response to VAC

The systematic review identified 2,096 potentially eligible articles via the electronic search. After removal of duplicate publications and non-relevant articles through title and abstract screens, 444 eligible articles were identified for full-text review. Of these articles, slightly more than half (*n* =242; 54.5 %) were Portuguese-language texts and the rest in English or Spanish language (Fig. 1. PRISMA search diagram). After review of the full-text, a total of 97 articles were included in the final data extraction set. One article provided data for four countries [11]. The majority (*n* = 79; 81.4 %) of the articles identified were from Brazil.

### Health services and linkages to other service sectors

Descriptions of health services and linkages to other services, such as child protection, were identified in peer-reviewed publications from Brazil, Colombia, Dominican Republic, El Salvador, Guatemala, Mexico, Nicaragua, and Venezuela (Table 2). Years of publication for these articles ranged from 2005-2012 and years in which the study or evaluations were implemented ranged from 2001-2009, though several articles did not report the year(s) of implementation, thus preventing identification of whether country protocols were developed prior to or after the conduct of the study. Publications that described the study design utilized for the research, reported using the following: observational studies, qualitative approaches, site evaluations, survey research, and/or mixed methods approaches (combination of qualitative and quantitative approaches). Additional file 1: Table S1 summarizes the identified studies that describe services for VAC and linkage to services.

**Table 2:** Numbers of articles per country reporting services provided in response to VAC, linkage to care/support, and training for health providers related to VAC and interventions

Country	Services for VAC & linkage to care	Education & curriculum	VAC focused interventions
Brazil	53	15	11
Chile	ND	1	1
Colombia	1	1	2
Dominican Republic	ND	ND	ND
El Salvador	1^a^	ND	ND
Guatemala	2^a^	ND	1
Honduras	1^a^	ND	ND
Mexico	1	ND	1
Nicaragua	2^a^	ND	ND
Peru	ND	ND	2
Puerto Rico	ND	1	ND
Trinidad & Tobago	ND	ND	1
Venezuela	1	1	ND

^a^ One study provided data for 4 countries; ND: no data identified from articles meeting eligibility criteria

### Identification of VAC

In Colombia and Chile, researchers described the development and use of screening tools to identify cases of VAC in the health sector; however no results in terms of impact on identification of child survivors, provision of care directly or via referrals were included in the studies [50, 51]. A study from Colombia described the mandatory reporting and surveillance system for VAC [52]. Results described an improved ability to document a wide range of VAC cases, increased visibility of VAC, and ability to inform advocacy and policy on sexual violence. Improved adherence to national protocols and improved cross-sector coordination were also noted [53]. The surveillance system, however, was reported to be a ‘failure’ in Cerrito and Candelaria, Colombia, where there was a lack of political will for implementing the system [53].

### Response to identified or suspected VAC

Approximately one-fourth of studies (*n* = 27; 27.8 %) reported the types of services offered within the health setting and linkages to other service sectors. One review from Colombia provided a brief description of the all-in-one center that combined healthcare and law enforcement in one location to respond to VAC. The study noted, however, that there remained a shortage in terms of skilled human resources, particularly legal investigators, to serve survivors and families [54]. Further, the authors noted that this all-in-one approach presented situations in which the perpetrator and survivor may cross paths in the center [54]. In another study, researchers conducted an international evaluation (El Salvador, Guatemala, Honduras, and Nicaragua) to assess medical facilities and adherence to guidelines on response to sexual violence (SV) among women and children [11]. This evaluation described the clinical services offered in response to SV and linkage to other services across the countries, as well as highlighted gaps, particularly lack of dissemination of guidelines and implementation strategy. Findings from this work reportedly led to health sector protocol revisions in 2009-10 for each of the four countries.

Four other studies conducted in Guatemala, Mexico, Nicaragua, and Brazil assessed the attitudes and behaviors of health care professionals toward VAC. The target professionals for these studies predominantly included nurses and described the barriers/challenges to identification and reporting of VAC in these diverse countries [55–61]. Challenges highlighted in studies included the lack of collaboration between health and other sectors, including child protection services and police; low levels of reporting of suspected cases to authorities even when mandatory reporting is required; poor dissemination of protocols to diverse clinical settings with lack of training and support for protocol implementation, including identification, assessment, treatment, referral, reporting and follow-up care for child survivors and families. Service providers explained their failure to implement protocols as the result of a lack of training on VAC, fear of becoming involved in legal issues, fear of reprisal by family or perpetrator, and/or beliefs that VAC was a private family affair [57].

Qualitative and mixed methods studies (*n* = 21) from Brazil described conflicting results on services for survivors of VAC. For example, in Brazil, the Program of Integrated and Referential Actions (PAIR), a national program that aims to integrate all services for children survivors of violence was evaluated over 3 years (2003 – 2006). The evaluation found that PAIR is a useful entity for integrating and strengthening access of children and families to the Tutelary Council - the justice system council responsible for investigating suspected VAC cases in Bahia State [62]. However, other research identified gaps between institutions, in particular the Tutelary Council and the Family Health Program, for reporting VAC. For example, among 23 professionals who provided care for cases of VAC in Family Health Program, only 8 (23 %) reported referring the survivor to the Tutelary Council, Police Office, or Public Prosecutor’s Office, limiting onward access to justice and protection systems [63]. Response to inquiries about reasons for not referring was low; however, 15 professionals did provide insight about low referrals. Reasons included lack of official instrument available for reporting the occurrence of VAC (4/15, 27 %), fear of retaliation by the aggressor and lack of evidence (7/15, 47 %), and lack of awareness of referral obligations (2/15, 13 %). Costa and others [64] described the “Multi-familiar Program” in Brazil, a pilot study of a therapy intervention that includes the use of play and theatre therapy for child survivors. Child survivors of sexual violence who had completed the judicial process were referred to receive the therapy from the Multi-familiar Program, which included counseling and play therapy. Qualitative feedback from participants (children and families), however, suggested that earlier initiation of therapy for child survivors was needed and authors argued that it was unnecessary for child survivors to wait the lengthy duration of the judicial process in Brazil to receive therapy. No evidence of the effectiveness of the therapy for child survivors was provided by the authors [64]. A subsequent study of cognitive behavior therapy (CBT) and play therapy by Costa and colleagues [65] demonstrated positive outcomes, including child and family reporting greater communication within the family, as well as with others in group therapy.

### Interventions to address and respond to VAC

Nineteen reviewed publications described interventions within the health sector or linking community and health sector to prevent and respond to VAC. These publications included studies implemented from 2003-2013 in Brazil, Chile, Colombia, Guatemala, Mexico, Peru, and Trinidad & Tobago and are presented in Additional file 1: Table S2 [50, 51, 53, 66–80]. Studies from Brazil and Guatemala focused on the evaluation of a combined intervention to develop a subnational interdisciplinary network of providers involved in the response to VAC and to implement the new VAC protocol [66, 77]. In Brazil, the interdisciplinary network of providers for response to sexual violence for children (aged <18 years) was associated with increased reporting and service seeking by survivors, increased numbers of referrals, and increased speed in identification of perpetrators [66]. Agusti [77] described the use of a comprehensive program for response to sexual violence that included active case finding, psychological support, reproductive health and use of a provider network in Guatemala to care for child and adult survivors of sexual violence. Over half of the survivors identified within two years of implementation were under 18 years of age and included both male and female survivors. However, post-intervention results were not provided on the impact of this comprehensive program [77].

With respect to mental health services, Habigzang and colleagues [72, 76] conducted several studies of provision of group, cognitive behavioral therapy (CBT) among young female survivors of sexual violence in Brazil, aged 9-16 years, and found reductions in symptoms consistent with depression, stress, and post-traumatic stress disorder (PTSD) [69, 76].

Three studies described community-based interventions. Celia and colleagues [73] described *Vida Centro Humano,* a program that targeted poor populations in Porto Alegre, Brazil. The project provided integrated health education, culture, leisure, science, technology, and professional training to address social factors such as poverty to prevent violence [73]. No results of the intervention were provided with the description of the program [73]. Two publications reported on the *Juntos*, cash transfer project, conducted among low-income caregivers of children aged 19 years and younger of the primarily Quechuan-speaking region of Chuschi, Peru [79]. A distinguishing feature of the *Juntos* program, as reported by the authors, was the inclusion of community facilitators who provided support in linking beneficiaries to program implementers; in sum, improving communication between the two groups. The publication noted the potential benefits of this economic intervention and reductions in VAC, but no study has been conducted to assess the impact of cash transfer on VAC [79]. Gomez (2010) also described the Chilean intervention, *Viviendo en Familia,* which was grounded in ecological theories of violence and neglect [50]. The intervention focused on providing a multi-faceted approach to services and addressing various social determinants that increase risk to violence. Each program serves 70–80 children and their families for 12–18 months, offering: comprehensive individual and family assessment; psychosocial counseling and workshops; individual and family counseling; home visits; and brief psychotherapy. The intervention was provided in eight brief sessions over the course of 12–18 months to male and female children, under the age of 18 years, and their families after identification of VAC. No results of the intervention were provided [50].

Lastly, the *Breaking the Silence* (BTS) intervention in Trinidad & Tobago targeted 3 communities consisting of 13 villages and utilized a community-based approach to develop the intervention model [80]. Activities included education, skills building, and service provision offered by an interdisciplinary team comprised of counselors, health professionals, child care workers, social workers, and nurses trained to work with individuals and families in the community for health promotion and disease prevention. The intervention activities were targeted to all members of the community and reflected the general needs identified by the three communities involved in the intervention model design. The model intervention activities introduced gender and gender ideologies and how these ideologies affect differential risks, attitudes, and treatment approaches related to VAC. Preliminary assessment of the intervention found overall improvements in knowledge and awareness of VAC, capacity building, and motivation to act in response to VAC. In each community, community members had established and sustained support services to identify and help survivors of VAC [80].

### Education, training, and curriculum on response to VAC

Nineteen publications discussed either training (*n* = 16; 16.5 %) or development and testing (*n* = 3; 3.1 %) of new health provider training curriculum on topics relevant to VAC [52, 81–98]. Additional file 1: Table S3 provides the list and summaries of articles. The majority described cross-sectional surveys to assess baseline training and/or describe past experiences of training for professional development. Several of these articles reported that health professionals, including doctors, nurses, and dentists did not have specific training or had only limited training on identification and response to VAC. This lack of training resulted in their reports of low capacity to provide adequate care to the child survivor and family [69, 86, 87, 90, 96]. Other reports noted that health professionals were unaware of steps necessary to respond to suspected VAC [87, 96].

One Brazilian study explored the perspectives and experiences of medical and nursing faculty related to implementation of pre-services training on intra-familial violence [93]. Although not formally part of the curriculum, less than half of the faculty of medicine (40 %) and the majority of nursing faculty (71 %) reported discussing family violence in their classes. However, faculty reported feeling ill-equipped and discomfort when students raised the issue of VAC, consequently limiting opportunities to strengthen pre-service training [93]. Such reports among medicine and nursing faculty reinforce the importance of pre-service training. One study from Puerto Rico found that despite requirements stipulated by the national protocol that all health professionals be certified in the provision of post-rape care, 73 % of participating physicians and 67 % of nurses reported no training in clinical management of sexual assault for adults or children [96]. Among this same sample of physicians and nurses, only one-third (34 %) of those surveyed were aware of the process for conducting assessments and physical exams for sexual assault and approximately 75 % (81 % physicians and 74 % nurses) reported that they had ignored physical signs and symptoms of sexual assault, despite that 66 % correctly identified emotional indicators of sexual assault [96]. Though formal reasons for low recognition of physical signs were provided, lack of training likely explains this lack of capacity. Only 31 % of participating health providers (27 % physicians and 32 % nurses) reported ever receiving training and, of these, 50 % had received only one training session about sexual abuse.

Only three articles reviewed presented results of training evaluations [83, 97, 98]. Cardoso and colleagues [83] described training that was targeted to community health workers in Brazil, which aimed to increase capacity and self-efficacy to address violence across the life course. The training did not focus solely on VAC; however, the topic was included in a comprehensive 20-h curriculum that also included violence against women, violence against the elderly, professional ethics, communication and teamwork. No specific results related to the impact of training on response to VAC were provided, though participants reported feeling well-qualified and more valued in their work at the end of the training [83].

One article from Colombia provided a proposal for an integrated in-service nursing and law curriculum on prevention and response to violence across the life course, addressing gaps between sectors described above [95]. No details were provided on the implementation or impact of the proposed study, nor the integrated educational program [95]. Lidchi and colleagues [89] utilized the training curriculum developed by the International Society for the Prevention of Child Abuse and Neglect (IPSCAN) and Centro de Estudos Integrados, Infancia Adolescencia e Saude (CEIIAS) to address child protection within broader health, education, and social services in Brazil. The development of the curriculum and training took a ‘bottom up’ approach, utilizing information gathered during pre-training surveys to inform the development of training curriculum and foster an integrated approach. For example, curriculum was developed to address 1) participant concerns/beliefs about identification and legal response to VAC; 2) beliefs and gaps in knowledge related to VAC and response; 3) provide information about service activation and response, coordination, and 4) review existing procedures for response to VAC [89]. However, no post-training evaluation results were provided to assess the impact of this training for participants or the quality of services provided [89]. Finally, Fernandez and colleagues (2007) described a study which sought to provide training on a socio-educational intervention with simulation, dioramas and mind maps that facilitate self-diagnosis of mistreatment among women and children and promote family values [97]. Training was conducted among medical students, medical residents, nurses and other health professionals in Venezuela over 20 sessions. The study described that the training intervention resulted in reductions in violence, strengthening of family values, and improvements in school performance, however, the authors provided limited description of methods and analysis used for the evaluation to support findings provided [97].

## Discussion

The purpose of this review was to describe the national health sector’s response to VAC within the LAC region using both national protocols and published articles. This review has revealed that, in spite of the prevalence and sequelae associated with VAC, best practices for responding to VAC particularly in resource-constrained areas remain limited. The content of national protocols reviewed and findings of health sector response varied tremendously with the majority of protocols providing limited guidance for VAC response to health care professionals; these findings validate the need for developing and implementing evidence-based policy and protocols that include opportunities for pre- and in-service training, tools to enhance identification, survivor-centered treatment and care, referrals to other service sectors and ongoing follow-up and psychosocial support to the child survivor and family as appropriate. Strengthening national protocols through broad dissemination of protocols with training on implementation and adherence, as well as research and monitoring will enhance the health sector’s effectiveness in prevention and response to VAC.

### Overview of national protocols

Our review of national protocols found that across the pool of submitted protocols, core components, such as definitions of VAC or specific types of violence, and signs and symptoms of VAC were included, though to varying degrees. Age limits and considerations of power dynamics were also included in these definitions and are important features which may facilitate greater awareness of violence, abuse and neglect, particularly in situations where physical injuries or symptoms of violence, abuse and neglect may not be apparent. Inclusion of definitions of VAC in protocols is critical to preventing ambiguity in health providers’ understanding of what constitutes violence, abuse and neglect and, ultimately, strengthening their understanding of role and responsibility in response once VAC is identified. We also noted multiple gaps in the national protocols on identification and management of suspected cases of VAC within health care settings; particularly in cases without obvious injuries or other signs/symptoms. As most health care professionals likely inspect for substantial physical evidence, such as injuries to justify suspicion, evidence suggests that not all forms of violence have obvious signs and symptoms [99–101]. Evidence remains lacking on how to best guide providers on identification of cases of suspected VAC in cases where physical signs/symptoms are absent. It remains important to include within protocol guidance instructions for health care professionals to be attuned to “minor” injuries or patterns of health care visits, injuries that do not match explanation, or vague complaints that can also be associated with VAC.

The level of detail and extent of guidance for treatment, referral and follow-up care was heterogeneous across protocols and often reflective of the target population anticipated to utilize the protocol. The plan of care for survivors of sexual violence presented in 15 of the submitted LAC country protocols aligned with recommendations for clinical management of sexual violence [102]. However, only two protocols (Chile and Trinidad & Tobago) specified clinical management for child and adolescent survivors of sexual assault. All other country protocols did not provide any clear delineation between clinical management of adolescents (14 years and older) versus younger children. More evidence is needed in low and middle-income countries to determine best practices for responding to the child and adolescent sexual violence among other forms of VAC. To this end, WHO is currently developing policy and clinical guidelines on child maltreatment and sexual violence against children and adolescents [103, 104].

### Evidence for recommendations within national protocols

#### Evidence for plans of care

Multiple protocols included recommendations for follow-up care for the child after violence/neglect has been suspected. Protocols from five countries recommended home visits (Chile, El Salvador, St. Vincent & Grenadines, Uruguay, and Peru) and cognitive behavior therapy (CBT) (Chile) for survivors and family members [26, 39, 42]. The recommendation for home visits was based on findings from the Olds Home Visit Model [105]. However, the Olds Model includes multiple home visits addressing several issues, such as maternal well-being and parenting skills for example. None of the protocols reviewed described what prevention and response activities related to VAC should be included in the home visit. Given that the model requires extensive training for home visitors prior to implementing, the Olds Model as is, may not be practical for settings with limited resources for training of health care professionals [105]. Cluster randomized controlled trials from other resource-limited settings have demonstrated reductions in specific unintentional injuries, such as burns and poisonings, among children aged 10 years and younger through the use of a paraprofessional home visitation program (HVP) [106]. Similarly, in the U.S. the SafeCare protocol, which aims to prevent child maltreatment and increase protective factors, was adapted and implemented among low-income Latino families [107]. Employing paraprofessionals with diverse educational backgrounds, the study demonstrated feasibility for use among this population. Taken together, such approaches may be adapted for home visits targeting VAC prevention across diverse settings in the LAC region.

#### Trauma-informed practices

There is a dearth of evidence on which to base recommendations on best practices for service provision for prevention and response to VAC. However, there is a growing body of literature on “trauma-informed care” (TIC) and the use of a “trauma-informed approach” to caring for survivors, with evidence suggesting improvements in post-traumatic symptoms and behavior problems among child survivors, patient-provider relationships [108], and broader improvements in collaboration across sectors [109, 110]. TIC is a commitment from the health care organization and all staff at all levels to reduce the risk of re-traumatizing the child when providing treatment and follow-up services [111, 112]. In this model, staff members are  expected to understand the potential impact of trauma on individuals, families, and communities and be able to recognize the signs of trauma and responding appropriately. More importantly, the organization or system offering TIC, must have policies and procedures that would not further exacerbate the sequelae from trauma [113]. Components of TIC include creating a safe environment for the survivor, fostering trust and transparency with the survivor and his/her family, allowing the survivor to be a partner and be involved in the decision-making about his/her care, and being culturally-sensitive.

Though most research to-date comes from settings outside of LAC, it is a possible that such a model could be adapted to settings in the LAC region. In fact, this review found that elements of TIC were apparent in almost all (90 %) of the protocols. For example protocols from 20 countries recommended creating a safe environment for survivors and providing patient-centered care. Also, protocols recommended conducting an interview in a way that is non-judgmental and encourages the patient to tell his/her story about the violence/neglect. Less apparent in the protocols was guidance on fostering trust and transparency with survivors and family members, as appropriate. Such recommendations would typically describe methods about how the provider should communicate with both the child and family member/caretaker to inform them of why inquiries about violence/abuse were being made. Additionally, recommendations should encourage communication to inform the patient/family that the disclosure, history, and assessment information will be used ultimately to help the child survivor recover and prevent reoccurrence rather than focusing on identification and punishment of the perpetrator [9].

#### Mandatory reporting

Mandatory reporting of suspected VAC to either a child protection agency or the police was present in about half submitted protocols. One rationale for mandatory reporting is that it communicates that VAC is not tolerated and obligates providers in the health sectors and other sectors to act on the care through reports to authorities [101, 114]. Gilbert (2009) raises the potential harms associated with providers' mandatory reporting of VAC as follows: “we do not know whether the process from recognition to reporting and subsequent interventions by child protection agencies improves lives of children overall.”(2009, p.168). This point is particularly relevant in low-resource settings in which alternative options for safety for child survivors (e.g., foster care, group homes, safe houses, shelters) may not be available when children are removed from homes. Further, evidence from the systematic review suggests that providers have limited awareness of reporting requirement or confidence in their ability to respond to VAC and concern about potential legal proceedings, even when reporting is mandatory. As a result, health professional describe low levels of reporting VAC to appropriate authorities [69, 86, 87, 90, 96]. Wekerle [114] argues that mandatory reporting should be conceptualized as part of a continuum of care, such that it is not the only intervention but the starting point to promoting resiliency and supporting the child survivor.

### Gaps & strengths of the current health sector response to violence against children in the LAC region

Despite the existence of guidance on services for VAC survivors in some protocols, our systematic review found that most publications initially identified by the search (and subsequently excluded) often reported statistics related to types of VAC reported in the health sector, child characteristics, and risk factors for VAC but did not focus the types of services provided to the child, including referrals and follow-up care. This dearth of information does not necessarily imply that such services are not being provided to child survivors; however, it does highlight the low prioritization of evaluation of service provision and health sector intervention for VAC and, ultimately, the gap that exists between research, policy, and programming. The development and implementation of health sector protocols and programs, however, require ongoing evaluation and monitoring, at the minimum, for continued quality improvement.

A clear gap across protocols was the limited dissemination of the protocol to target audiences, lack of strategies for implementation and of health professional and other service sector training. Findings from the systematic review mirrored these observations. In fact, most salient was the finding from the review of the existing scientific literature in LAC that most health professionals have limited to no training with respect to providing care for VAC, are unaware of reporting requirements and protocols, and/or of steps for reporting or intervening on suspected VAC cases [69, 86, 87, 90, 96]. Further, health professionals who do report cases or suspected cases of VAC are often frustrated by the lack of follow-up by child protection or child welfare systems, as they are left unaware of what happens to the survivors after the report and fear that the child remains at risk for VAC. Health professionals indicate that the lack of follow-up on cases leads to their hesitation to report VAC. Taken together, these issues highlight the importance of developing a clear dissemination plan for national protocols, appropriate interdisciplinary training centered on the components of the national protocol, and feedback mechanisms for the health sector and professionals who are involved in the VAC case.

An important strength to build upon is the recognition in protocols and in the literature that an interdisciplinary response to VAC is needed. Often noted in the studies from Brazil was the importance of training on response for VAC for diverse health professional providers, such as dentists and other health providers such as audiologists, with whom children come in regular contact [93, 115, 116]. Nurses were also noted to be at the forefront of care and well positioned to identify potential VAC; however, given limited access to protocols and training on referral pathways, nurses are not always aware of how to effectively respond with the goal of increasing child safety [72, 117, 118]. Indeed, collaboration across diverse health sector providers can strengthen prevention and response to VAC.

Research from Brazil provided evidence of positive outcomes associated with interdisciplinary collaboration and linkage to respond to VAC. Findings from the Program of Integrated and Referential Actions (PAIR) in Brazil, a national program that aims to integrate all services to children survivors of violence, suggests that this approach was successful in strengthening access of children and families to the Tutelary Council for investigation of suspected cases of violence/neglect [62]. As a cross-sectional study, however, assessment of long-term improvements and impact is limited. Broader insights on systems-level approaches may be obtained from the literature on IPV, reviews of which found that successful programs had onsite, dedicated staff who had the expertise to support the survivor. Such support may not be feasible in all LAC health care settings; however, professionals could, at a minimum, be provided with a protocol that included contact information for national or local hotline or programs with experts to provide guidance on cases of VAC [119]. All country protocols addressed the importance of community involvement in responding to VAC. Indeed, the BTS model from Trinidad & Tobago, which provided an interdisciplinary team of health professional and social workers to partner with the community for education and skills building, is one example of a community-based effort to prevent and respond to VAC [80]. Subsequently, this program has been scaled up in all Eastern Caribbean countries with support by UNICEF [120]. A partnership between such community groups and health care settings would be a way for both to effectively respond to VAC.

### Limitations

This review may be viewed in light of several limitations. Overall, protocols and peer-reviewed publications were available for approximately half of the target countries. This leaves questions about the availability and quality of national protocols on VAC in several LAC countries for which data were missing. Further questions remain on availability and types of guidance for countries lacking protocols and on the forms and quality of service provided to child survivors and families, given that no assessment was found to evaluate the quality of care from the survivors’ perspective. Findings from submitted protocols and identified publications may not be generalized to the entirety of LAC countries. We initially envisioned that peer reviewed publications would enable us to map what is being done in the field juxtaposed to the recommendations included in the national protocols. However, there was a substantial gap in the literature on the types of services provided, quality of care, referrals, and follow-up care for survivors of VAC. This gap in the literature prevented such mapping efforts and leaves questions about what services across the multiple sectors are actually being provided and how these services are related to the national protocols. Moreover, identified publications tended to be of relatively small sample sizes and report cross-sectional studies and qualitative research, limiting assessment of effectiveness, impact, and coverage of services.

## Conclusions

VAC remains an urgent international, public health priority. Health professionals are well-positioned to prevent and respond to the occurrence VAC in collaboration with multiple sectors. In response, WHO is currently developing policy and clinical guidelines on child maltreatment and sexual violence against children and adolescents [103, 104]. In light of this urgency and increased international attention, the study used both national protocols and publications to assess the health sector’s response to VAC among LAC countries, in order to provide guidance and recommendations to strengthen national health sector response to VAC. Though protocols often had clear descriptions for identification of VAC, several gaps were noted in health sector response and particularly in training of health professionals that resulted in poor compliance to guidelines and low perceptions of responsibility and self-efficacy in responding to VAC. Simultaneous development of evidence-based approaches and services for VAC with wide dissemination of national protocols and pre-and-in service training can strengthen multi-sector prevention and response with the goal of improving health and social outcomes across the life course for survivors of VAC.

## Additional file

Additional file 1: Contains systematic review search terms, country protocol descriptions, and data tables describing identified peer reviewed literature describing health sector response, interventions to address VAC, and education/training on VAC response for health professionals. (DOCX 170 kb)

## Abbreviations

IPSCAN: International society for the prevention of child abuse and neglect
LAC: Latin America and the Caribbean
GBV: Gender-based violence
PAHO: Pan American Health Organization
PTSD: Post-traumatic stress disorder
STI: Sexually transmitted infection
SV: Sexual violence
VAC: Violence against children
VAW: Violence against women
