# Supplementary material for: Violence against children in Latin America and Caribbean countries: a comprehensive review of national health sector efforts in prevention and response
Source: BMC Public Health. 2016 Sep 22;16:1006. doi: 10.1186/s12889-016-3562-3 (PMC5034460; doi:10.1186/s12889-016-3562-3)
Supplement: Additional file 1: — Contains systematic review search terms, country protocol descriptions, and data tables describing identified peer reviewed literature describing health sector response, interventions to address VAC, and education/training on VAC response for health professionals. (DOCX 170 kb) [file 12889_2016_3562_MOESM1_ESM.docx]

**Violence against children in Latin America and Caribbean Countries: A comprehensive review of national health sector efforts in prevention and response**

**Authors:** AL Wirtz*, C Alvarez*, AC Guedes, L Brumana, C Modvar, and N Glass.
* Joint first authors

**Additional File Contents:**

[***1. Search Strategy***	2](#_Toc459734438)

[***2. Protocol Descriptions by Country:***	7](#_Toc459734439)

[***3. Data tables***	13](#_Toc459734440)

[**Table S1.** Studies describing health services, provision of care, and referral to other services (N=59)	13](#_Toc459734441)

[**Table S2.** Studies describing exposure to training or developed and tested curriculum to train health providers on addressing VAC (N=19)	63](#_Toc459734442)

[**Table S3.** Studies describing interventions to address VAC in the health sector (N=20)	80](#_Toc459734443)

***1. Search Strategy***

**PubMed**

#1	"Child Abuse"[Mesh] OR "child abuse"[tw] OR "child abuses"[tw] OR "child neglect"[tw] OR "child maltreatment"[tw] OR "child mistreatment"[tw] OR "violence against children"[tw] OR "child sexual abuse"[tw] OR "Corporal punishment"[tw] OR "family violence"[tw] OR "harsh parenting"[tw] OR "abused children"[tw]

#2	("Violence"[Mesh] OR Violence[tw]) AND ("Adolescent"[Mesh] OR "Child"[Mesh] OR child*[tw] OR "Infant"[Mesh] OR adolesc*[tw] OR infant*[tw] OR teen*[tw] OR youth*[tw])

#3	#1 OR #2

#4	"Mandatory Reporting"[Mesh] OR "Health Personnel"[Mesh] OR "Mental Health Services"[Mesh] OR "Counseling"[Mesh] OR "Screening"[tw] OR "Referral and Consultation"[mesh] OR "Child Welfare" [mesh] OR "Child Health Services"[mesh] OR "Adolescent Health Services"[Mesh] OR "referral"[tw] OR healthcare[tw] OR "health care"[tw] OR "physician"[tw] OR "physicians"[tw] OR "doctor"[tw] OR "doctors"[tw] OR "assistant"[tw] OR "assistants"[tw] OR "nurse"[tw] OR "nurses"[tw] OR "social worker"[tw] OR "social workers"[tw] OR "provider"[tw] OR "providers"[tw] OR "clinician"[tw] OR "clinicians"[tw] OR "fieldworker"[tw] OR "fieldworkers"[tw] OR "field worker"[tw] OR "field workers"[tw] OR "child protection"[tw]

#5	"Americas"[Mesh:NoExp] OR "Caribbean Region"[Mesh] OR West Indies[Mesh:NoExp] OR South America[Mesh:NoExp] OR Latin America[Mesh:NoExp] OR Central America[Mesh:NoExp] OR "Antigua and Barbuda"[Mesh] OR Argentina[Mesh] OR Barbados[Mesh] OR Belize[Mesh] OR Bolivia[Mesh] OR Brazil[Mesh] OR Chile[Mesh] OR Colombia[Mesh] OR Costa Rica[Mesh] OR Cuba[Mesh] OR Dominica[Mesh] OR Dominican Republic[Mesh] OR Ecuador[Mesh] OR El Salvador[Mesh] OR "French Guiana"[Mesh] OR Grenada[Mesh] OR Guatemala[Mesh] OR Guyana[Mesh] OR Haiti[Mesh] OR Honduras[Mesh] OR Jamaica[Mesh] OR Mexico[Mesh] OR Netherlands Antilles[Mesh] OR Nicaragua[Mesh] OR Panama[Mesh] OR Paraguay[Mesh] OR Peru[Mesh] OR Puerto Rico[Mesh] OR "Saint Kitts and Nevis"[Mesh] OR Saint Lucia[Mesh] OR "Saint Vincent and the Grenadines"[Mesh] OR Suriname[Mesh] OR "Trinidad and Tobago"[Mesh] OR Uruguay[Mesh] OR Venezuela[Mesh]

#6	Caribbean[tiab] OR West Indies[tiab] OR South America[tiab] OR Latin America[tiab] OR Central America[tiab] OR Antigua[tiab] OR Barbuda[tiab] OR Argentina[tiab] OR Aruba[tiab] OR Bahamas[tiab] OR Barbados[tiab] OR Belize[tiab] OR Bermuda[tiab] OR Bolivia[tiab] OR Brasil[tiab] OR Brazil[tiab] OR Cayman Islands[tiab] OR Chile[tiab] OR Colombia[tiab] OR Costa Rica[tiab] OR Cuba[tiab] OR Dominica[tiab] OR Dominican Republic[tiab] OR Ecuador[tiab] OR El Salvador[tiab] OR Grenada[tiab] OR Guadeloupe[tiab] OR Guatemala[tiab] OR Guiana[tiab] OR Guyana[tiab] OR Haiti[tiab] OR Honduras[tiab] OR Jamaica[tiab] OR Martinique[tiab] OR Mexico[tiab] OR Montserrat[tiab] OR Netherlands Antilles[tiab] OR Nicaragua[tiab] OR Panama[tiab] OR Paraguay[tiab] OR Peru[tiab] OR Puerto Rico[tiab] OR Saint Kitts[tiab] OR St Kitts[tiab] OR Nevis[tiab] OR Saint Lucia[tiab] OR St Lucia[tiab] OR Saint Vincent[tiab] OR St Vincent[tiab] OR Grenadines[tiab] OR Suriname[tiab] OR Surinam[tiab] OR Trinidad[tiab] OR Tobago[tiab] OR Turks[tiab] OR Caicos[tiab] OR Uruguay[tiab] OR Venezuela[tiab] OR Virgin Islands[tiab]

#7	#5 OR #6

#8	#3 AND #4 AND #7

**Embase**

#1 Child abuse terms:

'child abuse'/exp OR 'family violence'/exp OR ("child abuse" OR "child abuses" OR "child neglect" OR "child maltreatment" OR "child mistreatment" OR "violence against children" OR “child sexual abuse” OR “Corporal punishment” OR “family violence” OR “harsh parenting” OR “abused children”):ti,ab

#2

('violence'/exp OR violence:ti,ab) AND ('adolescent'/exp OR 'child'/exp OR 'infant'/exp OR child*:ti,ab OR adolesc*:ti,ab OR infant*:ti,ab OR teen*:ti,ab OR youth*:ti,ab)

#3 Latin America terms:

'South and Central America'/exp OR (“Caribbean” OR “West Indies” OR “South America" OR "Latin America" OR "Central America" OR "Antigua" OR "Barbuda" OR "Argentina" OR "Aruba" OR "Bahamas" OR "Barbados" OR "Belize" OR "Bermuda" OR "Bolivia" OR "Brasil" OR "Brazil" OR "Cayman Islands" OR "Chile" OR "Colombia" OR "Costa Rica" OR "Cuba" OR "Dominica" OR "Dominican Republic" OR "Ecuador" OR "El Salvador" OR "Grenada" OR "Guadeloupe" OR "Guatemala" OR "Guiana" OR "Guyana" OR "Haiti" OR "Honduras" OR "Jamaica" OR "Martinique" OR "Mexico" OR "Montserrat" OR "Netherlands Antilles" OR "Nicaragua" OR "Panama" OR "Paraguay" OR "Peru" OR "Puerto Rico" OR "Saint Kitts" OR "St Kitts" OR "Nevis" OR "Saint Lucia" OR "St Lucia" OR "Saint Vincent" OR "St Vincent" OR "Grenadines" OR "Suriname" OR "Surinam" OR "Trinidad" OR "Tobago" OR "Turks" OR "Caicos" OR "Uruguay" OR "Venezuela" OR "Virgin Islands"):ti,ab

#4 Health sector

'mandatory reporting'/exp OR 'health care personnel'/exp OR 'health service'/exp OR 'counseling'/exp OR 'patient referral'/exp OR 'child welfare'/exp OR ("Screening" OR "referral" OR healthcare OR "health care" OR "physician" OR "physicians" OR "doctor" OR "doctors" OR "assistant" OR "assistants" OR "nurse" OR "nurses" OR "social worker" OR "social workers" OR "provider" OR "providers" OR "clinician" OR "clinicians" OR "fieldworker" OR "fieldworkers" OR "field worker" OR "field workers" OR "child protection"):ti,ab

(#1 OR #2) AND #3 AND #4

**LILACS	http://lilacs.bvsalud.org/en/**

Limited to 2005-2015: 928 hits

((("Child Abuse" OR "Maltrato a los Niños" OR "Maus-Tratos Infantis" OR MH:I01.198.240.856.350.250$ OR MH:I01.880.735.900.350.250$ OR MH:I01.880.787.293.292$ OR MH:SP9.120.060$ OR "child abuses" OR "child neglect" OR "child maltreatment" OR "child mistreatment" OR "violence against children" OR "child sexual abuse" OR "Corporal punishment" OR "family violence" OR "harsh parenting" OR "abused children") OR ((Violence OR Violencia OR MH:I01.198.240.856$ OR MH:I01.880.735.900$) AND (Adolesc$ OR MH:M01.060.057 OR MH:SP4.011.127.413.609$ OR Child$ OR Niño$ OR Criança$ OR MH:M01.060.406$ OR MH:SP4.011.127.413.654$ OR Infant$ OR Lactante$ OR Lactente$ OR MH:M01.060.703$ OR teen$ OR youth$))) AND ("Mandatory Reporting" OR "Notificación Obligatoria" OR "Notificação de Abuso" OR MH:I01.880.604.583.080.134.300$ OR MH:I01.880.604.583.527$ OR MH:I01.880.604.622.249$ OR MH:N03.706.535.615$ OR MH:N03.706.657.249$ OR "Health Personnel" OR "Personal de Salud" OR "Pessoal de Saúde" OR MH:M01.526.485$ OR MH:N02.360$ OR MH:SH1.030.020.020.010$ OR MH:VS3.004.001$ OR "Mental Health Services" OR "Servicios de Salud Mental" OR "Serviços de Saúde Mental" OR MH:F04.408$ OR MH:N02.421.461$ OR MH:SP2.031.267$ OR Counseling OR Consejo OR Aconselhamento OR MH:F02.784.176$ OR MH:F04.408.413$ OR MH:N02.421.143.303$ OR MH:N02.421.461.363$ OR "Referral and Consultation" OR "Remisión y Consulta" OR "Referência e Consulta" OR MH:N04.452.758.849$ OR "Child Welfare" OR "Bienestar del Niño" OR "Bem-Estar da Criança" OR MH:I01.880.787.293$ OR "Child Health Services" OR "Servicios de Salud del Niño" OR "Serviços de Saúde da Criança" OR MH:N02.421.143.130$ OR MH:SP2.031.222.143$ OR "Adolescent Health Services" OR "Servicios de Salud del Adolescente" OR "Serviços de Saúde do Adolescente" OR MH:N02.421.044$ OR MH:SP2.031.232$ OR Screening OR referral OR healthcare OR "health care" OR physician$ OR doctor$ OR assistant$ OR nurse$ OR "social worker" OR "social workers" OR provider$ OR clinician$ OR fieldworker$ OR "field worker" OR "field workers" OR "child protection") AND (DA:2005$ OR DA:2006$ OR DA:2007$ OR DA:2008$ OR DA:2009$ OR DA:2010$ OR DA:2011$ OR DA:2012$ OR DA:2013$ OR DA:2014$ OR DA:2015$))

**CINAHL**

#1	(MH "Child Abuse+") OR (MH "Child Abuse Survivors") OR (MH "Neglect (Omaha)") OR (MH "Abuse (Omaha)") OR (MH "Domestic Violence+")

#2	TI ( "child abuse" OR "child abuses" OR "child neglect" OR "child maltreatment" OR "child mistreatment" OR "violence against children" OR "child sexual abuse" OR "Corporal punishment" OR "family violence" OR "harsh parenting" OR "abused children" ) OR AB ( "child abuse" OR "child abuses" OR "child neglect" OR "child maltreatment" OR "child mistreatment" OR "violence against children" OR "child sexual abuse" OR "Corporal punishment" OR "family violence" OR "harsh parenting" OR "abused children" )

#3	((MH "Violence+") OR TI (violence) OR AB (violence)) AND ((MH "Adolescence+") OR (MH "Child+") OR (MH "Minors (Legal)") OR TI (child* OR adolesc* OR infant* OR teen* OR youth*) OR AB (child* OR adolesc* OR infant* OR teen* OR youth*))

#4	S1 OR S2 OR S3

#5	(MH "Mandatory Reporting") OR (MH "Health Personnel+") OR (MH "Mental Health Services+") OR (MH "Counseling+") OR (MH "Referral and Consultation+") OR (MH "Child Welfare+") OR (MH "Child Health Services+") OR (MH "Adolescent Health Services")

#6	TI ("Screening" OR "referral" OR healthcare OR "health care" OR physician* OR doctor* OR assistant* OR nurse* OR "social worker" OR "social workers" OR provider* OR clinician* OR fieldworker* OR "field worker" OR "field workers" OR "child protection") OR AB ("Screening" OR "referral" OR healthcare OR "health care" OR physician* OR doctor* OR assistant* OR nurse* OR "social worker" OR "social workers" OR provider* OR clinician* OR fieldworker* OR "field worker" OR "field workers" OR "child protection")

#7	S5 OR S6

#8	ADD Country terms – controlled vocabulary

#9	Add country terms – TI/AB

#10	S8 OR S9

#11	S4 AND S7 AND S10

**PsycINFO**

#1	DE "Child Abuse" OR DE "Battered Child Syndrome" OR DE "Child Neglect" OR DE "Pedophilia"

#2	TI ( "child abuse" OR "child abuses" OR "child neglect" OR "child maltreatment" OR "child mistreatment" OR "violence against children" OR "child sexual abuse" OR "Corporal punishment" OR "family violence" OR "harsh parenting" OR "abused children" ) OR AB ( "child abuse" OR "child abuses" OR "child neglect" OR "child maltreatment" OR "child mistreatment" OR "violence against children" OR "child sexual abuse" OR "Corporal punishment" OR "family violence" OR "harsh parenting" OR "abused children")

#3	(DE "Violence" OR DE "Domestic Violence" OR DE "Intimate Partner Violence" OR TI violence OR AB violence) AND (AG childhood OR AG adolescence OR TI (adolesc* OR infant* OR teen* OR youth*) OR AB (child* OR adolesc* OR infant* OR teen* OR youth*))

#4	S1 OR S2 OR S3

#5	DE "Abuse Reporting" OR DE "Child Abuse Reporting" OR DE "Health Personnel" OR DE "Allied Health Personnel" OR DE "Medical Personnel" OR DE "Mental Health Personnel" OR DE "Mental Health Services" OR DE "Community Mental Health Services" OR DE "Counseling" OR DE "Community Counseling" OR DE "Group Counseling" OR DE "Peer Counseling" OR DE "School Counseling" OR DE "Professional Referral" OR DE "Professional Consultation" OR DE "Child Welfare" OR DE "Child Guidance" OR DE "Child Care Workers" OR DE "Health Care Services" OR DE "Health Service Needs"

#6	TI ("Screening" OR "referral" OR healthcare OR "health care" OR physician* OR doctor* OR assistant* OR nurse* OR "social worker" OR "social workers" OR provider* OR clinician* OR fieldworker* OR "field worker" OR "field workers" OR "child protection") OR AB ("Screening" OR "referral" OR healthcare OR "health care" OR physician* OR doctor* OR assistant* OR nurse* OR "social worker" OR "social workers" OR provider* OR clinician* OR fieldworker* OR "field worker" OR "field workers" OR "child protection")

#7	S5 OR S6

#8	PL Caribbean OR PL West Indies OR PL South America OR PL Latin America OR PL Central America OR PL "Atlantic Islands" OR PL Antigua OR PL Barbuda OR PL Argentina OR PL Aruba OR PL Barbados OR PL Belize OR PL Bolivia OR PL Brasil OR PL Brazil OR PL Chile OR PL Colombia OR PL Costa Rica OR PL Cuba OR PL Dominica OR PL Dominican Republic OR PL Ecuador OR PL El Salvador OR PL Grenada OR PL Guatemala OR PL Guiana OR PL Guyana OR PL Haiti OR PL Honduras OR PL Jamaica OR PL Mexico OR PL Netherlands Antilles OR PL Nicaragua OR PL Panama OR PL Paraguay OR PL Peru OR PL Puerto Rico OR PL Saint Kitts OR PL St Kitts OR PL Nevis OR PL Saint Lucia OR PL St Lucia OR PL Saint Vincent OR PL St Vincent OR PL Grenadines OR PL Suriname OR PL Surinam OR PL Trinidad OR PL Tobago OR PL Uruguay OR PL Venezuela

#9	TI (Caribbean OR "West Indies" OR "South America" OR "Latin America" OR "Central America" OR "Atlantic Islands" OR Antigua OR Barbuda OR Argentina OR Aruba OR Barbados OR Belize OR Bolivia OR Brasil OR Brazil OR Chile OR Colombia OR Costa Rica OR Cuba OR Dominica OR "Dominican Republic" OR Ecuador OR "El Salvador" OR Grenada OR Guatemala OR Guiana OR Guyana OR Haiti OR Honduras OR Jamaica OR Mexico OR "Netherlands Antilles" OR Nicaragua OR Panama OR Paraguay OR Peru OR "Puerto Rico" OR "Saint Kitts" OR "St Kitts" OR Nevis OR "Saint Lucia" OR "St Lucia" OR "Saint Vincent" OR "St Vincent" OR Grenadines OR Suriname OR Surinam OR Trinidad OR Tobago OR Uruguay OR Venezuela) OR AB (Caribbean OR "West Indies" OR "South America" OR "Latin America" OR "Central America" OR "Atlantic Islands" OR Antigua OR Barbuda OR Argentina OR Aruba OR Barbados OR Belize OR Bolivia OR Brasil OR Brazil OR Chile OR Colombia OR Costa Rica OR Cuba OR Dominica OR "Dominican Republic" OR Ecuador OR "El Salvador" OR Grenada OR Guatemala OR Guiana OR Guyana OR Haiti OR Honduras OR Jamaica OR Mexico OR "Netherlands Antilles" OR Nicaragua OR Panama OR Paraguay OR Peru OR "Puerto Rico" OR "Saint Kitts" OR "St Kitts" OR Nevis OR "Saint Lucia" OR "St Lucia" OR "Saint Vincent" OR "St Vincent" OR Grenadines OR Suriname OR Surinam OR Trinidad OR Tobago OR Uruguay OR Venezuela)

#10	S8 OR S9

#11	S4 AND S7 AND S10

***2. Protocol Descriptions by Country:***

A total of 21 national protocols from LAC were received. Below brief overviews of each country protocol are provided (Note: Mexico is listed below but the information is not from an official protocol but rather a provided website). Overall the majority of the protocols focused on severe cases sexual and physical abuse. There was less guidance on how to council parents and follow-up on “milder” forms of abuse, which are likely, still harmful to the child (for example psychological abuse or hitting a child in anger). In addition, considering that these protocols are primarily for low and middle income countries, protocols did not address alternatives to care for situations in which abuse may be identified in a rural area or establishments where there are no “trained” staff members available. Nevertheless, the majority of the protocols provide patient-centered and therapeutic guidance on how to inquire about potential child abuse, as well as, extensive lists on indicators or signs/symptoms of child abuse.

A preliminary overall comparison between the country protocols and literature, suggests that there remains a disconnect between developers of the protocols and the practicing health care workers. Despite the extant protocols the consistent theme across countries is lack of awareness among health care workers on how to handle problems as well as fear and discomfort with reporting.

**Anguila (2014)(Anguilla Min. of Health and Social Development et al., 2014)**

This protocol was intended for a broad audience including health care professionals, police officers, principals, teachers, and social workers. The protocol emphasizes documentation of findings, and reporting of cases to authorities – the Department of Social Development. All professionals are responsible for reporting suspected cases to police, failure to do so may result in imprisonment or a fine - $5000.00. Specific to the health care sector, "appropriately trained and skilled practitioners" should conduct medical exams in cases of sexual abuse. Further, hospitals should have a system for identifying repeat attenders.

**Antigua & Barbuda (nd)(Government of Antigua and Barbuda, (nd))**

This protocol was intended for a broad audience, however focused on the involvement of schools in addressing child abuse. Responding to this abuse was primarily the responsibility of social services. The role of health care providers focused on assessment of the victim – regarding the provider role how to conduct the history and reporting data from the assessment. All suspected cases of abuse are to be reported to the Citizen’s Welfare Division and the police for formal investigation.

**Argentina (2011)(Argentina Min. de Salud, 2011)**

The purpose of this protocol is to guide the care of victims of sexual abuse at any stage of life and up to 6 months after the episode. In addition to providing guidelines on how to conduct the intake to how to evaluate the victim, and collect forensic evidence, this protocol also highlights the need for an interdisciplinary team in order to facilitate an optimal health sector response to child and sexual abuse.

**Barbados (nd)(Marshall-Haris, (nd); Pemberton-Gaskin, (nd))**

This protocol targets a general audience and emphasizes reporting of child abuse, as well as, informing professionals who interact with children on how to identify the signs and symptoms of abuse. Specific to the health sector – medical practitioners are noted as those who should report injuries suggestive of abuse. In keeping with the focus on reporting, development of guidelines for a referral system are encouraged.

**Bolivia (2014)(Bolivia Ministerio de Salud y Deportes & Policía Nacional, 2010)**

This protocol provided an action plan for inter-organizational collaboration among those who influence public policy and health services; community members were also included. The protocol focused on violence prevention. Addressing child abuse specifically was not addressed.

**Brazil (2010)(Ministerio da Saude, 2002; Ministerio da Saude, Secretaria de Atenção à Saúde, & Departamento de Ações Programáticas e Estratégicas, 2010, 2011)**

This protocol presents the idea of a “line of care” – intended to demonstrate the ideal practice of the continuity of care that ought to be provided to victims of abuse. The protocol emphasizes the point of receiving the child; this “entry” into the line of care is space in which the victim is supposed to feel welcomed and the professional assumes responsibility for helping the victim resolve the situation. The protocol provides detailed guides on how to interview the victim, addresses the attitudes of providers, the diagnostic studies and treatment that should be offered, and a plan for follow-up.

**British Virgin Islands (nd)(Governement of the Virgin Islands, (nd))**

This protocol was intended to guide and support government agencies coordinated prevention, reporting, investigation, and management of child abuse/neglect, and therefore intended for an interdisciplinary audience. The report emphasizes documentation and reporting of child abuse across all sections. This protocol also addresses capacity building in the areas of interviewing techniques – especially forensic interviewing and procedures for the police, social workers, judiciary, and medical practitioners.

**Chile (2013)(Ministerio de Salud, 2011; Ministerio de Salud & Gobierno de Chile, 2013)**

This protocol was designed to contribute the protection and minimization of the consequences of child abuse through the detection, and appropriate care to children and adolescents who have been victims of violence by their family members or caretakers. The protocol provides guidance on reporting practices, how to interview, and examine the child. Follow-up on all cases is emphasized along with therapy options for the victims as well as family. This protocol also provides evidence for best practices/successful models of secondary prevention of child abuse and treatment.

**Colombia (2011)(Dirección General de Salud Pública & Ministro de la Proteccion Social, 2011)**

This protocol is intended to be a useful tool for the professionals and institutions who may come upon a case of child abuse; this tool in addition to professional and personal skills, and institutional support can facilitate humane and effective care which is essential to providing quality care at a time when one is most vulnerable. The protocol recommends that care should be available 24 hours a day and free of charge. An interdisciplinary response is emphasized, as well as, the professional roles needed to implement the ideal model for a response to sexual violence.

**Dominica (2005)(The Social Welfare Division, Ministry of Community Development, & Gender Affairs and Information, 2005)**

This protocol provides a general overview about the different types of child abuse as well as the potential indicators of abuse. Once indicators of abuse are identified, authorities are to be notified immediately; in addition the child’s information should be stored/documented in a registry. The welfare division is the designated department for receiving reports of child abuse. The general roles and responsibilities of health care workers are also provided, such as to identify and provide support for families at risk of child abuse and neglect and develop and conduct primary prevention programmes.

**Dominican Republic (2014)(Ministerio de Salud Pública, 2013; Ministerio Salud Pública, 2014)**

The protocol is intended for child and adolescent health care providers who work within the Violence Detection and treatment division within the National Health System. This protocol provides detailed guidance on history taking, assessment, diagnostic exams, and treatment of victims. These guidelines are also “tailored” to the severity of the case and include plans for follow-up. The use of this protocol is mandatory and includes suggested quality indicators to evaluate the efficacy of the protocol.

**El Salvador (2012)(El Salvador Ministerio de Salud, 2012, 2014)**

This protocol is intended to guide health care workers with the Social Services Integrated Network to carry out the health promotion and care for those experiencing domestic violence. The protocol provides guidance on how the health care worker should assess the victim, including guidance on diagnostic studies and how to treat STIs. There is also detail on follow-up care including therapy for the child and the family.

**Grenada (2011)(Grenada Child Protection Agency, 2011)**

The protocol is designed for the “prevention, reporting, investigation, and management of child abuse.” The protocol is also intended to be a collaborative tool where agencies are working together to resolve cases and not duplicating tasks. The protocol emphasizes that trained personnel should conduct the medical exam, and that interviews should be conducted in the presence of a police officer and/or child protection worker. The protocol also provides detail on reporting of child abuse, and follow-up interventions.

**Guatemala (2010)(Ministro de Salud Publica y Asistencia Social, 2010)**

This protocol was designed to guide the care of any victim/survivor of sexual abuse at anytime point in his/her life. The protocol highlights routine screening for child abuse and provides detail on how to classify potential indicators of abuse and the actions necessary for the severity of abuse; at the minimum suspected cases should be reported to social work. The protocol also provides guidance on the roles and responsibilities doctors and nurses may assume when handling a case of abuse. Further, the protocol also addresses inclusion of the parents or guardians in the plan of care, as well as, detail on how to treat infections.

**Guyana (2010)(*Guyana medical practioners guide to the Sexual Offences Act 2010*, (nd))**

This protocol was intended to provide medical professionals with a standardized tool/instrument for the purpose of accurately collecting and documenting evidence and dealing appropriately with the medical needs of the complainant including injury from the rape as well as preventative measures for HIV, other sexually transmitted diseases, and pregnancy. Follow up and counseling is recommended for up to six months after the event. The protocol highlights the importance of proper documentation.

**Mexico (1999)(Ministerio de Salud del Mexico, 1999)**

This protocol is intended to establish criteria for the care and guidance provided to those involved in situations of family violence. All institutions, agencies, and organizations of the national health system should be about to respond and attend to those involved in family violence. The protocol states what health care workers should be able to accomplish – identify those affected, assess one’s risk for violence, identify indicators of abuse. Specifics on how to conduct the recommended procedures are not provided.

**Nicaragua (2009)(Nicaragua Ministerio de Salud, 2009, 2013)**

This protocol is designed to strengthen the capacity of the health care workforce to diagnose and care for survivors of DV and sexual violence. The protocol provides extensive lists of the signs and symptoms of all types of abuse, and then provides the activities that should be completed once abuse is suspected. Mental health care is highlighted in follow-up care, and the inclusion of family in the plan of care and rehabilitation is recommended.

**Paraguay (2012)(Ministerio de Salud Pública y Bienestar Social Paraguay, 2012)**

This protocol is intended to support health care efforts towards the prevention and support of gender-based violence and domestic violence among women and children. Health care workers are provided guidance on how to conduct the history taking, physical exam, collection of evidence and informed on the necessary diagnostic tests. Mental health care is also highlighted as a need.

**Peru (2001 & 2007)(Ministerio de Salud del Peru, 2001, 2007)**

This protocol is intended to guide the health sector approach to different levels of family violence and child abuse thereby contributing to the prevention and recuperation of survivors of child and/or sexual abuse. The protocol provides guidance on how to conduct the history taking, highlights working with the survivor/victim on a safety plan and encouraging him/her to make his/her own decisions. There is also guidance on follow-up care including mental health services and home visits. Throughout this protocol team-based care is emphasized.

**St.Kitts & Nevis (nd)(Ministry of Community Development, (nd))**

This protocol was intended to “provide the minimum standards for prevention, investigation, reporting, judicial intervention, care, treatment and support of each case of child abuse and neglect.” The protocol specifies the roles of the Ministry of Health such as assessing the ongoing needs of the survivor/victim and referring the child and family to appropriate community resources. For this nation, questioning is to be reserved for those conducting the investigation. All medical personnel, teachers, police, clergy should report abuse within 24hrs to the Probation & Child Protection Service.

**St.Vincent & Grenadines (nd)((unknown), (nd))**

This protocol is intended to present guidelines and procedures to be used for investigating, identifying, and handling child abuse. The protocol focuses on documentation of findings, and following a report system – the physician report is then reported to the district medical officer for example. It is also implied however, that not all suspected cases of abuse warrant immediate attention.

**Trinidad & Tobago (2012)((unknown), 2012)**

This protocol is intended to guide health care professionals in providing comprehensive care to survivors of sexual violence. This protocol recommends that only a “registered” doctor conduct the exam; a “pre-exam” is also recommended where the survivor’s mental health or emotional needs are addressed first to “stabilize his/her emotional equilibrium.” Guidelines are also provided for diagnostic studies and prophylactic treatments. There are also guidelines regarding when the survivor should be admitted to a health facility. This protocol also addresses the need to train providers and developing a sexual assault team, there are also plans for a "one-stop-shop.”

**Uruguay (2009)(Ministerio de Salud Publica del Uruguay, 2009a, 2009b)**

This protocol focuses on VAC as all action or omission of actions - psychological, physical, sexual, abandonment or neglect - that are intentional and continuous and committed by the parents or caregivers resulting in the compromise of basic physical, social, or emotional needs of the child/adolescent. The protocol describes basic care and support that should occur at initial intake in a primary care setting and recommend referral to another institution for optimal care, including children’s hospital, and private health clinics, with follow-up by social workers. Similar to other protocols there is extensive detail on the symptoms to be investigated in suspected cases of child abuse - including community factors such as poor/insufficient social networks. The protocol includes recommendation on when and when not to admit a child to the hospital, for example - admit when the injuries require extensive care, complex assessments are needed, if the child lives with the accused, if the child does not live in a protective environment; Do not admit if - there is no diagnosis of abuse, the child does not live with the accused, if a protective environment exists for the child, etc.

**Venezuela (2013)(Ministerio del Poder Popular para la Salud, 2013)**

This protocol is intended to inform the prevention and optimal care (information, counseling, psychological support, medical attention, and legal advise) for those experiencing abuse and domestic/family violence. The protocol offers guidelines on assessing the risk for repeat abuse, conducting the history taking, physical exam, identifying discrepancies in the history, all suspected cases of abuse should be reported immediately.

***3. Data tables***

| **Table S1.** Studies describing health services, provision of care, and referral to other services (N=59) | | | | | | | | | | | | | | | | | | |
| --- | --- | --- | --- | --- | --- | --- | --- | --- | --- | --- | --- | --- | --- | --- | --- | --- | --- | --- |
| **Study (Author, year pub)** | **Country** | | | **Year(s) of study** | | **Study Objective** | **Study design, participants** | **Services offered** | **Linkage to other services** | | | **Identified gaps/limitations** | | | | **Results / Impact** | | **Other relevant summary of study** |
| Nunes, C.B. et al 2009 | | | Brazil | 2003 | | The objective of the study was to understand how heath care professionals approach family violence against children and teenagers. | Qualitative case study with 30 health care professionals (nurses, dentists, doctors, social workers, unit managers, nursing assistants, dental assistants and community health workers). | NR | NR | | Lack of integration between professionals and the several sectors that should address these issues; Lack of integrated work on the team itself; Lack of structure that enables the community health worker to act with greater safety; Absence of an approach by professionals, that prioritizes the suspected or confirmed cases of violence; Disregard for the real problem of children and their families, due to pre-established norms; Lack of effectiveness in solving some situations, given the lack of partnership with external bodies; | | | | | | NR | NR |
| Silveira, J. L. G. C., Mayrink, S., Sousa, N. O. B. (2005) | | | Brazil | 2003 | | To describe the child abuse in Blumenau - Santa Catarina, valuating the dentist knowledge and attitudes in these situations with healthcare professionals from SUS - Health Unified System and from the private sector | Cohort study design. Records from the Legal Medical Institute were analyzed in the year of 2003. | NR | NR | | | Limitation is due the level of study design. | | | | 35% of dentists reported ever attended some case of child abuse. In relation to the dentists’ attitudes to deal with the cases of VAC, 23% informed to wait and observer, 29% reported to the Tutelary Council, 19% searched for more information in order to know what to do; 29% didn’t know how to react. 84% considered themselves unprepared to deal with cases of VAC. 6% reported to fear consequences in report the cases, 32% did not know how to proceed and 42% reported to feel unsecured to report the cases for competent authorities | |  |
| Gomes, V. L. O., Fonseca, A. D. (2005) | | | Brazil | 2004 | | To investigate the perception of caregivers of an Infantile Education Institution and teachers of a Primary School regarding the concept of violence, as well as the conduct adopted by them in relation to violent situations against children and adolescents | Qualitative study design among caregivers in Institutions | NR | NR | | | Limitation is related to the level of study design | | The study showed that when participants were inquired if they have witnessed violence against children, physical aggression provoked by the mother or father in the interaction with children was reported. In addition, when questioned about if these caregivers have performed any act of violence against children, the participants demonstrated justify their attitudes as a mechanism of self-defense, when they reported to have not wanted to yell with the children but have done anyway. | | | | The study concluded that the concept of violence is misunderstood among these teachers and caregivers in the school. Violence is associated to physical aggression and appearance of signals such as hematomas and bruises |
| Andrade, E. M. et al 2011 | | | Brazil | 2005 | | To understand the experience of health professionals concerning domestic violence against children and teenagers, describing the identification of the cases and the assistance offered to the victims in the Unidades Básicas de Saúde (UBS - Primary Health Care Units). | The techniques used were participant observation, focal groups and semi-structured interviews conducted at two primary healthcare units in the city of Embu, Metropolitan Region of São Paulo. Participants (N-23) were the directors of the primary care units, who took part in a semi-structured interview, and health care professionals (4 doctors, 2 nurses, 4 nursing assistants, 4 community health agents, 2 dentists, 1 psychologist, 1 speech therapist and 3 administrative assistants) who took part in the focal groups. | It is mentioned that professionals reported identified cases to the Child Protection Agency or to the police. However the main focus of the study was their feelings in relation to the management of cases of violence.  In addition the professionals mentioned that the creation of a Reference Center for Children Victim of Violence could be an alternative to the problems they face when dealing with this issue. According to this study, there is no data related to specific interventions, implementation of protocols, or education and training. | NR | | | Several difficulties reported in tackling the question of violence, such as fear of getting involved with criminals, lack of protection in the services where they work and the families' lack of commitment. Also cite reproduction of cultural standards in which physical punishment is accepted as an educational practice. Participants do not feel responsible or qualified to deal with the problem. It was also observed that professionals attribute the limitation of their work to the lack of mental health professionals who can best complement the multidisciplinary work. The assistance offered to the victims is restricted, mainly due to the professionals’ difficulties in handling the social-cultural characteristics of the population and to the lack of connection with the institutions that are responsible for the cases. | | | | The professionals mentioned that the creation of a Reference Center for Children Victim of Violence could be an alternative to the problems they faced when dealing with cases of violence. | | It highlights the need for continuing education for healthcare professionals, related to the recognition and reporting of signs and symptoms of child abuse and neglect. It was also mentioned that doctors, teachers or the person responsible for health care and education services, according to Article 245 of the 1990 ECA, are obliged to report the suspicion or confirmation of domestic victimization of children and adolescents to the competent authorities. The service that should be referred to is Guardian Council in their area. |
| Costa, L. F., (2009) | | | Brazil | 2005 | | To examine the benefits and limitations of the Multifamiliar Group on the family unit. | Research-action methodology. Participants were children and teenagers victim of sexual violence. | The Multifamiliar Group provided counseling and uses playful activities such as theatrical performances by the victimized children to express their feelings and face their trauma. | Children victims of sexual violence and its family members were referred from the justice system to receive therapy on this Multifamiliar Program in Minas Gerais - Brazil. | | | Although the Multifamiliar Group is presented as a promissory initiative and has a strong collaboration with the Justice system in Brazil, the study was limited to a small sample to conduct focus groups (N=8) and it is limited to one district in the state of Minas Gerais. | | | | Families and children reported to feel safe in sharing their experiences at the Multifamiliar Group and the initiative has shown positive results in the collaboration with the justice system in Minas Gerais - Brazil. | | It is important to point out that the Multifamiliar Program is a pilot innovative. |
| de Souza, M. K., Santana, J. S. (2009) | | | Brazil | 2006 | | To analyze the participation of the local health managers in the assistance provided to the adolescent victim of violence in the city of Itabuna, Bahia, Brazil. | Qualitative study design. Participants were heath managers of the Municipal city of Itabuna, Bahia. | Centers of Psychosocial Attention (CAPS) is a service directed to the family in the care of individuals in psychological suffering. In this study, participants interviewed reported that CAPS is expanding their services in order to assist the needs of adolescent victims of domestic violence in some municipalities in Brazil. | The Sentinel Project is a policy initiative from the federal government and constitution of a set of specialized and multi-professional social actions directed to victims of sexual violence as part of the National Plan to Combat Sexual Violence against Children and Adolescents. | | | The study describes the need for policies specifically focused on violence against teenagers and the need to expand the services of CAPS in other municipalities. | | | | Healthcare professionals are not sensitized with the issue of violence against teenagers. They do not know how to care the adolescent victims of domestic violence. In addition, participants reported that there is a lack of standard procedures in the referral to healthcare services in the community. Healthcare managers reported to have linkage issues with the Juvenile Council but the Sentinel Project has been showing positive results in reducing these problems. | | Even being a qualitative study, it is still quite informative in regards policy initiatives within the healthcare system in Brazil. |
| Lima, P. D., (2008) | | | Brazil | 2006 | | To know the type of violence identified by the healthcare professionals in the confirmed or suspected cases of violence on hospitalized children; identify the conducts adopted by the healthcare professionals and identify the healthcare professionals that most help in the decision-making to deal with these cases. | Cross-sectional study design. Participants were 235 healthcare professionals who works in a hospital specialized in Children in Roraima - Brazil. | NR | Victims were referred to the Tutelary Council of the city | | | Limitation is related to the level of strength of the study design | | | | With respect to decision making in dealing with cases of VAC in the hospital, 55% of the social workers reported that nursing technicians were the category that most helped in the decisions of procedures to be taken in terms of reporting the case to the Tutelary Council and assist the victim; 33.0% of physicians reported that technical / auxiliary nurses were the ones that took more actions in to assist the cases of VAC. Fifth eight percent of the psychologists reported that the nurses technicians and nurses that were more directly involved in the procedures to assist the victims; 38% of the participants identified that nurses and nurses technicians were the professionals that reported of the cases at the hospital for the competent authorities. | | The study highlighted the prevalence of nursing in the assistance and report of suspected and confirmed cases of children victims of violence. |
| Souza, M. K., B., (2009) | | | Brazil | 2006 | | To analyze the participation of the local health managers in the assistance provided to the adolescent victim of violence in the city of Itabuna, Bahia, Brazil | Qualitative study design. Participants were healthcare managers of the public healthcare system in Itabuna. | NR | NR | | | Participants reported that children victims of violence are mainly victims of the poor Brazilian healthcare system. Lack of structure to assist the victims and unprepared professionals to deal with these cases were cited by the respondents. In addition, the lack of integration between social, justice and healthcare systems were also reported. | | | | Participants considered violence as any act of aggression from an individual to another in which the power of action is taken and consequently financial, emotional, physical damages are created. Participants also cited that if the healthcare system does not provide the appropriate support for children victims of abuse, damages will be reflected in their adolescence. According to participants these teenager who have suffered violence in their childhood are more likely to provoke violence against others as a result of their experiences as victims. Participants recommend that public policies should focus on these adolescents. | |  |
| Vieira, EM 2012 | | | Brazil | 2007 | | To explore health workers knowledge and attitudes towards women victims of intimate partner violence (IPV). | Mixed methods research: quantitative surveys with health care professionals (N=221) and qualitative interviews with doctors (N=14) to explore knowledge, attitudes towards health assistance for women victims of IPV | NR | NR | | | NR | | | | Study focused on VAW but found that 79.6% of providers felt that a security plan should be established for the female victim and her children | | NR |
| Sauret, G. V., (2011) | | | Brazil | 2007 | | To examine health-care professionals' representations of the families of children and adolescents who have been victims of violent crime at a public-sector health referral center in Recife, in the Brazilian State of Pernambuco. | Qualitative study design. Semi-structured interviews were conducted with psychologists, health professionals, teachers and social workers. | NR | NR | | | Limitation is due the level of evidence of the study design | | | | The study revealed that participants tend to dichotomize children and teenagers as the victims of sexual violence and those victims of other forms of violence. In addition, the study also identified in the interviews that there is a sense of extra care in the interaction of healthcare providers with children/teenagers victims of sexual abuse. The social worker interviewed informed that in terms of identification of the violence, it is easier to identify sexual abuse than psychological abuse in the families in their routine. Participants also revealed that some families abandoned the psychological treatment but return later when realize that the children still require care after the experience of violence. | |  |
| Lobato G. R., et al, 2012 | | | Brazil | 2008 | | Objective: to analyze the challenges in addressing domestic violence against children and adolescents by professionals of the Family Health Program (PSF). | Qualitative study approach in which individual interviews were conducted, using a thematic script composed of open questions, the material was subjected to content analysis with thematic approach. The participants were the professionals part of team of the Family Health Program (PSF), being: three doctors, three nurses, two nursing assistants, three nursing technicians and 14 community health workers (N = 25). | Some professionals mentioned the organization of educational group activities, targeting parents and especially adolescents as a strategy to promote health, and contribute to building peace in the families and communities. | They reported that these educational group activities are sometimes carried out in partnership with the Guardianship Council, the schools and the Court for Children, Youth and Elderly. | | | Almost all respondents stated that they do not feel empowered to deal with cases related to violence against children and adolescents. Some talked about the need for support from psychologists to guide professionals in the Family Health's teams, as well as the families involved. They stated that up to that moment they did not participate in any continuing education activity on the subject of domestic violence. The partnership of other institutions was highly valued by respondents, but considered insufficient, especially in actions targeting adolescents. According to the professionals, there is a of lack information to adolescents, but mostly lack of sports, and artistic activities, and professional courses as a way to get them out of idleness and prepare them for a better future. | | | | The respondents do not feel qualified to deal with domestic violence; Furthermore, there is no consensus among them about their responsibilities on these cases. Home visits and educational groups are their main strategies; inter-sectoral action, although present, are intermittent. | | NR |
| Acioli, et. Al, 2011. | | | Brazil | 2008 | | To describe the profile and the conduct of phonoaudiologists who work for the public health authorities in the city of Recife, in the Brazilian State of Pernambuco. | A cross-sectional descriptive survey with phonoaudiologists (N=89) from 22 public services and 4 philanthropic services working in partnership with the public sector. | When asked about the institutions responsible for the care of children and adolescents victims of violence professionals mentioned: the Guardianship Council (86 times, 96.6%), the Childhood and Youth Court (72 times, 80,9 %), the Management of the Children and Adolescents Police/Institute of Forensic Medicine (GPCA/IML) (69 times, 77,5%), the Prosecutor's Office (57 times, 64%) and the Health Unit of Reference (34 times, 38,2%). | Patients were referred to social or psychological services (24 cases, 34.3%) Health care worker:  - provided guidance to the family about how to proceed (18 cases, 25.7%),  - reported cases to the competent bodies (2 cases, 2.9%), - referred the patients to a doctor (3 cases, 4.3%), - did not take any action (14 cases, 20%). | | | Limitations of the design do not allow extrapolation of results, but authors believe that similar situations possibly occur in other regions of Brazil. | | | | Most phonoaudiologist presented good knowledge about the institutions that can be used in case of violence against children and adolescents, but results showed that they are unprepared to address the problem.  Therefore, authors suggest that the topic violence against children be part of the curriculum of undergraduate courses in phonoaudiology. In addition training courses and continuing education should be offered to professionals that are already in public and private health services. | | NR |
| Cocco, M. et al 2010 | | | Brazil | 2008 | | To analyze the flow of care in hospitals assisting children and adolescents who are victims of violence and professionals' feelings about dealing with child abuse. | Descriptive and exploratory study with quantitative approach, with the participation (N=34) of health professionals (doctors, nurses and nursing technicians) who work in the Emergency Care Unit and Pediatric Unit in three different institutions. | NR | 31% of participants report VAC cases to the Public Prosecution (Ministério Público); 25% report to the Guardianship Council; 12% complete the mandatory reporting form, communicate to the Guardianship Council and provide care to the patients; 6% refer to the Social Worker's Hospital and the Child Protection Agency, 6% communicate the Social Assistant of the Hospital and the Specialized service. | | | NR | | | | The study reveals that 91.66% of the subjects know that there is a compulsory notification form for cases of abuse and violence against children in the institutions. Responsibility for reporting: 66.66% of the subjects believe that this is the doctor's responsibility, followed by 25% who believe this is responsibility of the nurses, 37.5% believe this is the social worker's responsibility. It is observed that most professionals know the notification form, but do not believe it is their responsibility, passing it onto the nurses and the doctors. | | NR |
| Deslandes, S.,(2011) | | | Brazil | 2008 | | To present indicators for the evaluation of local government actions for reporting and recording cases of domestic violence and sexual exploitation of children and adolescents. | Community-based participatory research. The following experts throughout the country composed the study: Healthcare, Social Welfare, Education, Child Protection Council and Rights, Pole of Children and Youth, State Prosecutor, Public Defender, Tourism, Public Security, universities and civil society organizations. | NR | NR | | | Limitations are due the need of further discussion among the social, educational and healthcare sectors in regards domestic and sexual violence in children and teenagers. | | | | Experts identified three main indicators require attention. 1) The need for all government departments to have a standardized instrument to report situations of family violence. 2) Discusses the leadership of healthcare in the country and highlighted the need for properly trained teams focused in the assistance of children and teenagers victims of domestic and sexual violence. 3) The need of intrasectoral and intersectoral actions and interventions that demand and promote continuous data flows, especially with the agencies that compose the Rights Assurance System. | | The study concluded that investments in such government departments in order to integrate services of each sector is critical and urgent in Brazil |
| Lima, M. C. C. S., (2011) | | | Brazil | 2008 | | To analyze the performance of professionals of FHS and Basic Health Care Unit (HBU) in Feira de Santana for identifying the notification of children and adolescents victimized by physical and sexual violence | Cross-sectional study design. Participants were 583 healthcare providers at the Family Health Strategy in Feira de Santana. The sample included nurses, physicians, community agents, dentists and nursing technicians. In this study, 67.8% (N=320) of participants aged 30-49 years. The majority were female 88.9% (N=447) and more than 68.0% (N=345) had children. | NR | The study revealed that 45.3% of cases were reported to the Tutelary Council and 33.2% were reported to the police | | | Although the professionals that compose the Health Family Strategy team are able to identify cases, they did not feel safe to report the cases for competent authorities | | | | Among the participants, 27.9% (N=135) reported having received prior training to work at the Family Health Strategy and 96.4% (N=460) have shown interest in being trained to deal with VAC. 34.8% of the UBS employees and 42.3% of ESF reported past identification of a case of violence. Doctors (48.9%), nurses (48.8%) and ACS (41.6%) were the professionals that most identified children and adolescents victimized by physical and sexual violence. Over 50% of professionals reported that cases identified in the community were reported to the Guardianship Councils and Police; however, in most of the cases, the professional was not the person notifying the guardianship. Community Agents are more likely to identify cases of violence given that they are part of the community and spend more time with the families assisted by the Health Family Strategy team. | | Study does not describe specific care provided by healthcare providers. |
| Carlos, DM 2014 | | | Brazil | 2009 | | To assess the understanding of adolescents regarding the social support received in situations of domestic violence. | Qualitative research in Campinas, SP with 17 adolescents (aged 12-18) who were victims of domestic violence | NR | Child survivors of DV engaged with the Bureau of Social Welfare services (community centers, transit shelters, Casas de Passagem). Guardianship Council is main protection service to children/ adolescents man main location for reporting and follow-up of cases (though not followed-up in long-term) | | | Lack of monitoring and long-term follow-up of cases | | | | Services are placed at strategic locations, improving access for adolescents | | NR |
| de Silva, LM 2012 | | | Brazil | 2009 | | To identify the work developed by the Judiciary to prevent sexual violence against children and adolescents within the family. | Qualitative study with members from the Courts for Crimes against children and Adolescents (CCCAs) included judges, assistants, judicial technicians, analysts, interviews and FGDs (N=17) | Focuses on preventing worsening of damage and occurrences of new cases, interrupt violence behavior against children, promote reparation for victim and encourage seeking of legal punishment; | NR | | | Children/adolescents not seen as a priority for public policies; public funding and coordination for SV against children are redistributed and allocation not based on need; Seen as bureaucratic process and a legal case can take at least 4 yrs.; CCCAs not integrated with multidisciplinary teams; no separate facilities for victims and perpetrators - often overlap in buildings and can cause secondary trauma | | | | NR | | Study focuses on legal procedures and CCCAs, but does not provide much detail on linkages to other health services and multidisciplinary groups, other than to say is less common |
| Amorim de Ávila et al 2012 | | | Brazil | 2009 | | To examine professional practice of nurses from five primary health care units from a city in southern Brazil in relation to sexual abuse of children and adolescents. | This is a qualitative study, developed with nurses (N=7) working in the Family Health Strategy teams in the suburban areas of Rio Grande/RS. Data were collected through semi-structured interview, focusing on knowledge about intervention in case of suspected sexual abuse during childhood and adolescence, and the perception of nurses on the assistance provided to victims and their family. | NR | NR | | | There is no victim care protocol to support professionals, which makes the delivery of care difficult. | | | | Professionals feel unprepared, helpless and disappointed with respect to the measures implemented to confirm or refuse the suspected sexual abuse. Another highlight is that there is no victim care protocol to support professionals, which makes the delivery of care difficult. In addition, the study emphasizes the need for training courses for all professionals that provide clarification on how to handle these child abuse situations. | | Authors mention availability of some protocols/policies: - Política Nacional de Redução da Morbimortalidade por Acidentes e Violências. Portaria GM/MS 737, de 16/05/2001; - Política Nacional de Promoção à Saúde, instituída pela Portaria GM/MS 687, de 30/03/2006. - Notificação de violências contra crianças e adolescentes na rede do Sistema Único de Saúde (Portaria 1968/2001); - Rede Nacional de Núcleos de Prevenção das Violências e Promoção da Saúde (Portaria GM/MS 936/2004). |
| Gawryszewski, V. P., (2012) | | | Brazil | 2009 | | To describe the profile of the reports of child and adolescent abuse in the state of São Paulo in 2009, and to analyze possible associated factors | Cohort study design. A total of 4,085 reports regarding children and adolescents younger than 15 years recorded by the Domestic, Sexual, and Other Interpersonal Violence Surveillance System (VIVA) were analyzed. | NR | 70.7% of cases were referred to the following institutions: Guardianship Council (GC), Child and Juvenile Courts (CJC), and Public Prosecution Office (PPO) (25.8% of referred cases); special Police Departments and Forensics Institutes (27.5% of referred cases), and other institutions, such as shelters, surveillance programs, and Child Protection and Social Assistance Services (46.7% of referred cases). | | | Limited generalizability given that data were not collected universally, but in surveillance services in Brazil. | | | | The study indicated that health professionals are identifying suspected abuse, breaking the silence and reporting cases of violence in Brazil. | |  |
| Bezerra, K. P., (2012) | | | Brazil | 2009 | | To analyze the performance of nurses of the Family Health Strategy by facing family violence against children and identifying actions to prevent the problem | Qualitative study design. Participants were 20 nurses that worked in the Health Strategy Family and in the Family Health Units in Brazil. (Both are primary care clinics focused on family health). | NR | NR | | | Participants revealed the following barriers in dealing with VAC in the Family Health Strategy: Fear of reprisal by the aggressor of the violence in reporting the case; work overload as a barrier in recognize suspect cases and prevent violence against children, lack of managerial support and difficulty for the accomplishment of interdisciplinary, intersectionality and comprehensive care in primary care. | | | |  | | The study is focused on the experiences and perspective of nurses in the Family Health Strategy and revealed that there are considerable barriers in dealing with VAC. |
| Zanelatto, P. F. (2012) | | | Brazil | 2009 | | To understand the meanings attributed by Family Health Strategy professionals on violence against children and adolescents, and their attitudes toward violence evidences noted in health care context | Qualitative study design. Participants were 18 healthcare professionals who works in four Family Health Strategy teams in Goiânia - Brazil | NR | Participants revealed to have referred cases of VAC to the Tutelary Council. | | | Limitation is due the level of evidence of the study design | | | | Participants suggested to consider violence against children as any form of aggression in which emotional, physical and social harm is caused. In addition, they shared that lack of family plan and socio economic issues are the main triggers for VAC among the families assisted by the Family Health Strategy. Lastly, the healthcare workers pointed out that creating an open dialogue with the families is the most effective strategy to prevent cases of VAC. | | The study is informative and cited the Tutelary council as the main referral service for cases identified in the Family Health Strategy. |
| Paixão, A. C. W. 2011 | | | Brazil | 2010 | | The article analyzes the activities of the Municipal Department of Health and Civil Defense of Rio de Janeiro in ensuring specialized care for the population of children and adolescents who are the victims of sexual violence. | Eight semi-structured interviews were conducted with managers of the Program for Women, Adolescents and Children, the Health Promotion Advisory, and the Superintendence of Primary Health Care. These interviews identified the SMSDC-RJ actions for specialized care to children and adolescents in sexual violence situations, their families and perpetrators of this abuse. | NR | NR | | | Participants shared that there is fragile relationship between the State and the Municipality in the shaping of policies to address sexual violence. In addition, statements reflect a gap in the dialogue among pairs of the Municipal Secretary themselves, and little dissemination of the State Plan between municipal managers working in the same field. Statements reflect criticisms of the management systems: non-participation of all program managers in the creation of the plan; dissemination of the Plan to the Secretariat managers made in an informal way; difficulties in the process of sensitization of other institutions on the theme of violence; and possible gaps in its development in health care. | | | | **Service flow:** Within the organization's network it is necessary to create a flow of care, this is one of the actions cited by most respondents and considered by them as fundamental. **The assistance to families:** Participants expressed difficulty of managers to sensitize the area of mental health on the importance of this type of care. **Assistance to the Perpetrator:** Identified as an area of need. | | **Continuation of results/impact... Training:** There is institutional interest in encouraging the improvement of all its members. Health professionals directly involved, despite having received a first orientation and participated in some study groups, have yet to benefit from a more systematic educational support.  **Monitoring and evaluation of actions taken:** The vast majority of managers are unaware of any official assessment of SMSDC-RJ actions related to addressing infant-juvenile sexual violence. |
| Aragao, A. S. et al 2013 | | | Brazil | 2011 | | To analyze how primary care nurses approach cases of violence against children and adolescents, identifying limits and possibilities for dealing with these cases. | Qualitative study, used semi-structured interviews with nurses (N=8) in the Family Health teams in the city of Uberaba, state of Minas Gerais. Study sites were selected based on the location of the Program of Integrated and Referential Actions to Confront Sexual Violence against Children and Youth in Minas Gerais (PAIR), 2006/2007 and evidence from this program on areas of increased vulnerability. | Reports reveal that the actions of the nurses are restricted to notifications and/or referrals to: doctors, members of the Family Health Support Centers (NASF), as a psychologist and social worker, to the Reference Centers of Social Assistance (CRAS) and Guardian Council. | There is no mention about the discussion of cases with other members of the Health Team, nor is mentioned a shared action with other public or private entities, or even in other sectors, such as education or social protection. | | | Challenges are related to unawareness or discontinuity of the flows cause some respondents to act in isolation, disarticulated and making use of personal contacts. Gaps exist in the training of the professionals, who feel unprepared to carry actions related to the problem of violence, even in regard to the procedures involved in the notification | | | | In general, violence against children and adolescents is not recognized as a problem to be solved by nursing. Recurrently, respondents associate the problem to the competence of other professionals in the health teams, or outside of it | | NR |
| Salomão, P. R. (2014) | | | Brazil | 2012 | | To understand the nursing care provided to children/teenagers victims of violence living in orphanages and to identify the strategies used on the treatment of these individuals when they arrive at the institution. | Qualitative study design. Seven nurses who works at the Special Protection Foundation of the State of Rio Grande do Sul in the city of Porto Alegre were interviewed through focus group. | Nurses reported to often provide affection in their interaction with these children in order to fill the lack of familial care. Playing was also reported by the nurses as essential to help children and teenagers to deal with their emotions and to help in establishing a good relationship with these victims. | NR | | | Limitations reported in nursing assistance were related to the mental challenge in dealing with children and teenagers who experienced different types of violence. Nurses reported to not feel prepared to answer the children questions in regards the judicial process in which they are involved, as well to provide them psychological support and care. There is also a lack of standardized nursing care among this sheltered children and teenage population. | | | | Regarding nursing care, the nurses interviewed informed that there is a lack of standard protocols to follow and led their practice with the children and teenagers in the shelter. Regarding prevention of violence within the shelter, nurses reported that initially, the main focus in dealing with a child or teenager that is immediately institutionalized is to provide the best caring environment as possible in order to they feel able to express their individuality and their feelings with all the staff of the shelter. | | This study revealed through the perspective of nurses, how sheltered children and teenagers experience violence in institutions of assistance for children victims of violence as the challenge for nursing practice with this population. |
| Habigzang, L. F., (2006) | | | Brazil | 1992-1998 | | To present the mapping of risk and protective factors in network resources for children and adolescences victims of sexual violence, who were identified in the judicial processes of the District Attorney's Office from 1992 to 1998 in the state of Rio Grande do Sul, Brazil. | Cohort study design. Seventy one records between 1992 and 1998 were analyzed. | NR | All the cases analyzed were referred to the Public Ministry after the case be reported to the Tutelary Council | | | Limitation is related to the level of evidence of the study design | | | | The study showed that initially all the cases analyzed were reported at the Tutelary Council and after the reporting of the case, 98.6% of the victims were placed in shelters, 32.4% received psychological treatment, 31.1% were placed in another family, 25.7% of the cases were referred to the police department for investigation, 9.5% were referred to the Legal Medical Institute, 9.5% of the victims had the guard given to other relatives and 6.9% were referred to hospital (6.9%). Sexual violence was the reason for referral of 87 of the 94 cases to prosecutors. | | Cases of violence against children and teenagers were considerably referred to health and social services in the decade of 90. |
| Cunha, J. M., A (2005) | | | Brazil | 1993-2003 | | To reflect about the nursing care to the child who is victim of familiar violence from the analysis of scientific production concerning this subject. | Integrative review. Thirty seven publications addressing the role of nursing for children victim of familiar violence were analyzed. | NR | NR | | | Though nurses are prevalent in all areas of care for VAC, they are less involved in research. | | | | Nurses appeared as a fundamental professional to deal with children victims of violence. The study also showed the active presence of nurses at all levels of assistance. | | The study was included even having international and national studies in the analysis. The role of nurses in the VAC in Brazil is presented separated from the other study countries. |
| Zambon 2012 | | | Brazil | 2003-07 | | To study children and adolescents victims of domestic violence treated at the Referenced Pediatric Emergency Unit of the Hospital de Clínicas of the Universidade Estadual de Campinas and its specialized outpatient clinic. | Prospective study of child/adolescent (<15yrs) victims of domestic violence who were treated at UERP HC (N=95). Med rec review. | All treated at UERP HC PEP provided to 49% of cases; 48.4% tested for HIV; 44.2% tested for syphilis; 46.3% tested for HBV; 47.4% tested for HCV - PEP and STI testing most common for rape survivors | Child protective services call for 76.5% of cases; | | | NR | | | | NR | |  |
| Costa, M. (2007) | | | Brazil | 2003-2004 | | To estimate the prevalence of violence against children and adolescents through the records of Child Protection Councils, associating this violence with the age of the victims and their links with the aggressor. | Cross-sectional study design. Tutelary Council records were analyzed in order to estimate the prevalence of violence against children in Bahia state. Most of the cases registered were reported by denounce calls. | NR | Referral by Tutelary Council to inquiry commission. Cases are directly referred by the Tutelary Council to this commission are judged based on the findings of the police investigation, medical evidences and interviews with the victim and the suspected aggressor. | | | The authors pointed it out that lack of data in the records regarding ethnicity/race of the aggressor and referral to therapeutic services for the children limited the analysis of this study | | | | Reports were mainly (398) anonymous (30.8%). The study showed that from the 1525 cases analyzed, more than 40% were notified to the justice system (483 cases) and more than 25% were attended by an inquiry commission, part of the justice system in Brazil specifically created to deal with these cases. | | NR |
| Costa, M. C. O., . (2010). | | | Brazil | 2003-2006 | | To present the evaluation of PAIR actions implemented in Feira de Santana city, on political and institutional integration, strength and performance of the Network Care for the violence victims in the period of 2003-2006. | Mixed methods study design; Interviews were conducted with professionals from health services, community leaders and representatives of the commission of National Program of Integrated and Referential Actions (PAIR) in order to present the impact of the PAIR to children victims of sexual violence in Feira de Santana - Bahia state. | The PAIR is a national program that aims to integrate all services to children victims of violence in Brazil | The PAIR is responsible to integrate the social services and the justice services in the referral of children victims of violence in Brazil. | | | They pointed out that costs are considered a limitation of the PAIR program. | | | | The impact of PAIR is positive in integrate services of social care and justice for children victims of violence. The study found that the PAIR is a positive strategy in integrating and strengthening the access of children and families to the Tutelary Council and to the justice system. In addition the role of social media in encouraging denounces against child abuse was also reported as beneficial in facilitate access to these services. | |  |
| Carvalho de Lima, ML 2010 | | | Brazil | 2005-06 | | To conduct a situational diagnosis of the prehospital healthcare network for victims of accidents and violence in Recife, Brazil. | Cross-sectional survey and semi-structured interviews with managers and health workers in 13 hospitals (10 municipal, 3 state) and one mobile unit | Multidisciplinary team includes doctor, social worker, psychologist, nurse, nursing assistant; ambulatory system (SAMU) for response to victims of accidents/ violence | Refers to tutelary council (45.4%*), special police for children & adolescents (35.2%), general hospital (35.3%), specialized outpatient (35.3%) | | | Dentist, biochemist, lab tech, radiologists rarely available; further professional training in the handling of accidents or violence sought on individual basis as a result of institutional policies; Only two of services had protocols for dealing with victims of accidents and violence; Only four managers were aware of national policy for preventing accidents and violence | | | | Little provision of prevention and health promotion in facilities; only one facility provided services for families as a whole; Has physical structure complying with the required standards, such as having a reception area and a waiting room, medical records, screening for risk, social services, consulting rooms for doctors, dentists and psychologists, emergency rooms, treatment rooms, suture rooms, washing of materials, sterilization, storage space, pharmacy, a clinical pathology lab, cloak room, refectory and morgue. | |  |
| Costa, L. F., (2007) | | | Brazil | 2006-2007 | | To present reflections on the psychological and emotional conditions that families, with children and/or adolescents sexually abused, have before any therapeutic attendance | Research-action methodology. Children and teenagers victims of domestic violence that are at the end of judicial process and are referred to the Multifamiliar Group in order to get therapeutic assistance. | The Multifamiliar Group is a pilot project in conjunction to the Juvenile Court in the Federal District - Brazil. The program provides counseling to children victims domestic violence | Children and teenagers victims of domestic violence are referred to receive therapy on this Multifamiliar Program in Minas Gerais - Brazil. | | | Limitation is related to the level of evidence of the study design | | | | The study challenges the Justice System of Brazil to consider the vulnerability of children in the judicial process that can takes a long time (sometimes more than 5 years) to be concluded and only then obligates the victims to seek for free therapy. | | This is a pilot study in which researcher described the "rite of passage" of children from the judicial normative to a therapeutic assistance by participating of the Multifamiliar program. |
| Bannwart, T. H. 2011 | | | Brazil | 2007 and 2008 | | To describe and analyze the difficulties found in identifying and notifying abuse against children and adolescents in the opinion of pediatricians. | This is a qualitative study involving eight pediatricians from a charity hospital in a midsized municipality of the state of São Paulo. | The Child Protective Service is responsible for protecting children and professionals at the same time, avoiding professional exposure; and the police is esponsible for investigating the case. | NR | | | The medical culture leads doctors to treat the phenomenon of abuse as if it was a disease, a diagnosis to be confirmed by tests, limiting it to the biological aspects when it is a social problem instead. Challenges noted by the pediatricians were disbelief in the effectiveness of the Child Support Service, non-existence or inadequate training on child-abuse issues by the pediatricians, and personal limitations. | | | | Professionals thought that in order to report the case, confirmation of the suspected abuse is needed, which contradicts the law.  Only one participant reported he took a theoretical course in a Department of Legal Medicine and another participant reported cases he/she saw during his/her training, that is, he/she appar- ently expanded the concept during the residence period, learning about the topic during clinical practice. | | The professionals’ lack of training to deal with victims of violence might be caused by their lack of knowledge on how to proceed in such cases. In Brazil, the shortage of regulations setting technical procedures, the absence of legal mechanisms to protect those responsible for violence in health care facilities, and the lack of respect for professional secrecy are considered barriers for reporting. |
| De Oliveira, M. T., (2012) | | | Brazil | 2008-2009 | | To verify the association between assistance, referral and reporting of Intra-family Violence/IFV against pregnant adolescents, and the socio-demographic characteristics of 84 professionals of the Family Health Program/FHP in the agreste region of Pernambuco. | Cross-sectional. Participants were 82 healthcare professionals working at the Family Health Strategy in the state of Pernambuco | NR | NR | | | This is a cross sectional study. | | | | Twenty three out of 82 professionals interviewed reported to have attended some case of violence against children during their working lifetime (28%). 15 professionals reported that the reasons for not referring the cases involving adolescents in situations of violence were: lack of official instrument to fill in the moment of the occurrence of the situation (4/15); fear of retaliation by the aggressor and lack of evidence (7/15) and ignorance of the obligation to notify (2/15). | | The study points out the lack of linkage between the Tutelary Council and the Family Health Strategy in terms of reports of violence against children |
| Garbin, C. A. S., (2011) | | | Brazil | 2008-2009 | | To investigate the profile of the occurrence of abuse against children and adolescents. | Cross-sectional study design. All records of assaults against children and teenagers in 2008 from a police department in Aracatuba. Analyzed the data recorded in police reports from the Department of Women’s Defense, Araçatuba-SP, in 2008, relating to assault, victim-offender relationship, sociodemographic characteristics of offenders and victims, and victims’ demand for health services. | NR | NR | | | The study pointed out psychological violence as the most important type of violence and therefore requires special attention from the health care provider, since its manifestation, sometimes subtle, and often unnoticed. Reinforce the importance of healthcare professionals to detect and report the proven or even suspected cases to the competent authorities. | | | | The study showed that 59.8% of the violence occurred in the residence of the victims. 67.84% of cases of violence registered. Only 23 cases of the total sample sought healthcare assistance (7.39%). No other data on types of services provided /accessed were reported. | | The authors suggest that the lack of seeking for healthcare assistance it might be associated to the lack of trust of the families in the healthcare services to provide adequate psychological support to the victims and also due the non severity of physical lesions. |
| Harr, C 2008 | | | Brazil | NR | | To observe the different interdisciplinary approaches used by two major hospitals, one in the United States and the other in Brazil, in their attempt to confront the social problem of child maltreatment. | NR | Hospital database dedicated to collecting data on perpetrator; information also gathered to share with the Protective Councils/boards, as required by law. The Council, along with the DA/Prosecutor represents the child, when necessary, to take action to protect the child or possibly remove from the family | Hospital admission through the Emergency Dept., then referred to the Dept. of Social Services; interview of family conducted in conjunction with psychologist to determine if necessary to contact gov. agencies. Brazilian Statute of the Child and Adolescent states that health organizations and other competent agencies are required to report all situations where there is suspicion of violence against children/adolescents | | | No formal team within the hospital that deals with VAC; internal structural problems within Protective Council | | | | NR | | NR |
| Braha, N 2010 | | | Brazil | NR | | To investigate the thoughts and perceptions of professionals concerning the safeguarding systems in Bahia, barriers to recognition, reporting and management of VAC. | Cross-sectional, mixed methods: 10 FGDs, 8 semi structured interviews, and 55 questionnaires conducted with experts working to safeguard Bahia's children | NR | NR | | | Participants identified numerous barriers to effective safeguarding, including a strong fear of violent reprisals for reporting cases (despite anonymous reporting systems) and a lack of training and multidisciplinary working. There was a perception that the newly-established Child Protection Agency is ill-equipped to deal with the complexity of cases and that prosecutions are too slow, reducing motivation to report abuse. They felt that better training and stronger communications between professional groups working together to support the Child Protection Agency, would substantially improve the situation. | | | | NR | |  |
| Nunes, 2008 | | | Brazil | NR | | To understand the conceptions held by health professionals with regards to violence within the family against children and adolescents. | Qualitative research with health professionals (N=19, including community health agents, social assistants, dental assistant, dentists, nurses and nursing assistant, and physician. | NR | NR | | | Health professionals think about violence with preconceived views, not only regarding the poverty of families cared for by the Unit of the PSF, but also regarding the types of individuals who are usually identified as aggressors. This leads to a pre-disposition of  associating violence to certain contexts and individuals, without assessing what actually happened. | | | | NR | | NR |
| Azambuja, M. P. R. 2005 | | | Brazil | NR | | To discuss the difficulties of management of child abuse situation and, more specifically, sexual abuse. | Based on an experience in a Community Psychology practice, the author discusses the difficulties of management of child abuse situation and, more specifically sexual abuse, despite the existence of “Child and Adolescent Law”, (ECA-Brazilian regulation related to children and adolescent) which determines that any case of child abuse, even if it’s only a suspicion, must be notified to authorities. | The alternative found by many institutions, especially hospitals, is the creation of interdisciplinary committees. Thus, besides having greater understanding of the case, made possible by the collaboration of professionals from different areas, the responsibility for reporting to the Guardian Council is not the responsibility of only one professional. | NR | | | Authors indicate that even though the law named ECA (Brazil, 1990) requires mandatory notification, even in cases of suspected violence against children, this is not what happens in reality. Even with mandatory notification, even in cases of suspected abuse, the author states that this is not what happens in reality. Professionals who work in a health center usually located in needy communities have close contact with the community, leading to fear of reprisals/ threats by the offenders. Recommended alternative for this problem of exposure/ fear found by many institutions, especially hospitals, is the creation of interdisciplinary committees. | | | | The author describes a case in which the report to the Guardianship Council was done in partnership between the health unit, the school and the recreation center, so that the responsibility would be shared and possibly this would help the family to realize the gravity of the situation, and generate better acceptance of the facts. However a lot of resistance was found from the school and the recreation center. | | The author cites some institutions such as the Regional Centre of Attention to Maltreatment during Childhood (CRAMI), in Campinas, and the Multidisciplinary Brazilian Association of Childhood and adolescence Protection (ABRAPIA), in Rio de Janeiro. |
| Brito, A. M. et al. 2005 | | | Brazil | NR | | To understand predictors for child abuse from the perspective of families. To examine how the families received treatment offered by the institution; to assess the impact of intervention in the intensity of family violence. | Descriptive study, in which it was used the application of a questionnaire to the person legally responsible for the child (N=55). They were approached by phone or during a visit to their place of residency. | The work with families is developed by psychologists and social workers, through activities such as: follow-up performed through home visits, weekly, every other week, or monthly as needed; Weekly psychotherapeutic services for adults, children and adolescents, in individual sessions or in groups, as indicated; Focal groups weekly meetings with parents, held in two neighborhoods that have high incidence of reports of domestic violence; lectures, awareness and committees, aimed at professionals from various areas of care and protection to children and adolescents; materials prepared for guidance about violence an its implications, and with technical content in the areas of care for children and adolescents. | | NR | | NR | | | | Post-intervention assessment included: 61% reduction in attribution of violence to am conflicts between the couple, 58% reduction associated with the outbreak of violence to the child's or adolescent characteristics, 29% reduction in alcoholism. As for those who pointed out the life history as a triggering factor, 20% said it was overcome. | | The approach which established the proposed work is founded on the understanding that domestic violence against children and adolescents is a "symptom" modulated by difficulties in regard to cultural, social, economic aspects and difficulties in interpersonal relationship, causing conflicts in family relationships, therefore the care must be extended to the whole family group. |
| Milani, R. G; 2008 | | | Brazil | NR | | Objective: a) to characterize the psychosocial risk conditions and the protective measures applied by the Child Protection Agency three years ago to families reported for domestic violence; b) compare the resources and adversities of a group of families assisted three years ago by the Child Protection Agency for domestic violence, with a group of families that did not have psychosocial risk history related to domestic violence. | The investigation tecniques included: semi-structured interviews with the familiar environment resources inventory and the adverse events scale. | As for the protective measures implemented by the Child Protection Agency, in all cases, measures were related to legal advice, based on the ECA, and, as appropriate, the measures prioritized social or legal areas or mental health. | Regarding the social area seven referrals were made to social protection programs, four to social and educational programs, with full-time care for children, and three to temporary shelter, where they stayed for a few days. In total, they made fourteen referrals to mental health. Of these, six were directed to children, five referred to psychological care, because they have behavioral and / or emotional difficulties that preceded the event that motivated the complaint, and one was also referred for psychiatric care for presenting psychotic symptoms. The eight targeted referrals to parents involved psychological care, mostly for treatment of alcoholism. A total of fourteen referrals were made to mental health. Of these, six were directed to children, five referred to psychological care, because they have behavioral and / or emotional difficulties that preceded the event that motivated the complaint, and one was referred for psychiatric care for presenting psychotic symptoms. The eight referrals to parents involved psychological care, mostly for treatment of alcoholism. | | | | NR | | | NR | | It is noteworthy that the child protection agency, must be attentive so that, by focusing on the rights of children, they are also able to have the perspective of the family as a complex system that also has needs, so there is no risk of the institution becoming repressive. In this sense, it takes mental and psychosocial health measures, with interventions that mobilize the problem-solving ability of the family. |
| Ferreira, A. L., (2008) | | | Brazil | NR | | To analyze indicators used in research on services that provide care to such children and adolescents. | Exploratory study design. An exploratory study was conducted, selecting five studies in four services: two nongovernmental organizations in two cities in the interior of São Paulo State and two organizations, one nongovernmental and the other governmental, in the city of Rio de Janeiro. | The institutions studied gives the initial treatment and make monitoring visits in situations in which there is suspicion of violence (domestic or others) against children and adolescents. These actions carried out by health professionals. | Tutelary Council | | | In general, the services do not intend to accompany children and adolescents after the end of treatment, so it is unknown the changes in the situation of these patients. | | | | Eighteen indicators for "process of assistance" and nine for "results" were identified. The study concluded that some indicators are considered essential for a minimum assessment of the care provided by the services that accompany families involved in situations where there is suspicion or confirmation of violence against their children: follow-up duration (to assess the dynamics of care), proportion of regular monitoring and abandonment (to assess adherence), and proportion of high recidivism rate (to evaluate solutions) and proportion of reporting cases to the Guardian Council, especially those who are confirmed (to evaluate integration with the network). | |  |
| Ferriani, M., . (2008) | | | Brazil | NR | | To describe the health care offered to children and adolescents sheltered at the Care Center for Child and Adolescent Victims, in Ribeirão Preto-SP, Brazil | Qualitative study design. Semi-structured interviews were performed with professionals that works with children and teenagers in the shelter. | The educators are employees who works full-time with the sheltering; They are responsible for the care of children and adolescents. They also assist in food, in the bath, in bedtime, in school lessons, try to maintain order and avoid fights, and often administer medicines, accompany the children in medical appointments, etc. | NR | | | Limitations are due the level of evidence of the study design | | | | The study showed that the administration of medication is an issue that divides opinion among the shelter staff studied. In addition, great disorganization in regard to this topic were observed. The administration of medication ends up being done by those who are "available" at the time. The study found that in general, the staff considered the administration of medication a simple and routine task which can be performed in any manner by any person. They do not consider the importance of the procedure, maybe for do not understand its complexity. | | Regarding physical space, the study found many inadequacies in the shelter as well lack of sensitization and mobilization of governmental entities to develop a more appropriate structure in order to promote a better quality of life for children and adolescents. |
| Gava, L. L., DellAglio, D. D. (2013) | | | Brazil | NR | | To investigate the techniques used by psychologists in forensic examinations in cases of suspected child and adolescent sexual abuse in the context of the criminal investigation. | Qualitative study design. Participants were twelve psychologists who practiced as expert witnesses in the Medical-Legal Institutes of the following state capital cities: Natal, Palmas, Brasília, Goiânia, Florianópolis and Porto Alegre. | Psychologists at the Medical-legal Institutes are responsible for interviewing victims as part of the process of investigation of the cases of violence in Brazil | NR | | | Limitations is related to study design | | | | The study showed that in the investigations of situations of child sexual abuse, when considering the absence of physical marks and other witnesses, the interview is considered the main instrument of the psychologist. The observation of symptoms, although they may be associated with a traumatic event, does not provide information about a concrete and specific situation. In the majority of cases, therefore, it is only the testimony of the child that can make the violence suffered visible and audible. Participants reported that the use of ludic techniques to interview children and teenagers such as drawing, dolls and others are not often used, depending on each case. In addition, written report and clinical observation are also part of the Statement Validity Analysis to verify the veracity of the sexual abuse. | | Participants carry out the forensic examination assessment using diverse methods (interviews, tests, credibility assessment) and information sources (documents from the investigation or judicial proceedings, the alleged child victim and the parents/guardian). However, in some situations it was found that the delimitation of the methods used is driven by the previous knowledge of the professionals rather than by a technical decision, or by the influence of the judicial authorities, who sometimes ask for certain tests. It can also be observed, cases in which the lack of material, e.g., the lack of availability of the tests, prevents their use, causing the professional to resort to other available techniques or even to create new techniques to investigate the cases. |
| Gawryszewski, V. P. (2005) | | | Brazil | NR | | To identify information about homicide mortality from different data sources such as death reports, autopsy reports and police reports in the city of Sao Paulo | Cohort study design. 2.844 cases of homicide ocurred in the second semester of 2001 were analyzed. | NR | NR | | | The study did not specifically address violence against children and teenagers. Limitation is related to the difficult in establishing what kind of assistance the victims transported to hospitals received in these locations. That's because the autopsy reports showed only the name of the hospital and not the assistance provided. | | | | The study showed the prevalence of male predominance in 93.2% of the death reports observed. The values of mortality rates (per 100,000 population) are high: 57.2 for the total population; 111.1 for males and 7.4 for females. The hazard ratio for males is 15.4 times more than in female. More than half of these victims were teenagers and young adult males and men between 15 to 29 years concentrated 56% of all homicides. In addition, the coefficients in this population reached high values, peaking in the group of 20 to 24 years, with rates reaching 262.8 / 100,000; followed by 25 to 29, 226.8 / 100,000 and 15 to 19, 205.4 / 100,000. The age that represented the most number of deaths was 19 years old (mode). | | The study is mainly focused in aspects related to mortality for homicide in the city of Sao Paulo. Does not provide significant information about violence against children or teenagers. The only significant information is related to the prevalence of deaths of teenagers although the causes of these deaths among these age group were not assessed. Although, the consumption of alcohol and drugs among the overall victims of homicide is discussed. |
| Gawryszewski, V. P. S., (2006) | | | Brazil | NR | | To present the new Injury Surveillance System based on Sentinel Health Services, carried out by the Ministry of Health in order to broaden the knowledge of these causes | Essay. The article describe the process of implementation of the Injury Surveillance System (VIVA) based on Sentinel Health Services in Brazil | NR | The Injury Surveillance System promotes better surveillance and referral of victims between hospital and other healthcare institutions specializes in violence | | | Limitation is related to the level of the study design. | | | | The article highlighted the advantages of the system in provide quality of information and reliability of data about, as well the agility in to identify and report cases of violence identified in hospital or healthcare clinics. In addition, the study stated that purpose of the new system is to characterize the profile of victims of sexual violence, domestic and other interpersonal violence; to characterize the profile of visits resulting from accidents and violence in the selected hospital emergency services; validate the Notification Form for Investigation of Sexual Violence and/or Domestic Interpersonal Violence and the Accidents and Violence Notification for Urgent and Emergency Units form; test the operation of the system; and use the information in the definition of public policies. Therefore, the Service Sentinel works when two forms are filled: the first form is stored in the healthcare service and the other is forwarded to the epidemiological surveillance of the Municipal Health Department, in which typing and data analysis is performed in the VIVA program. In case of violence against child and adolescent, three form are need to be filled. The first one is required to be sent to the Guardian Council in the region as determined by the Child and adolescent (ECA). After entering the database and the first analysis be done, the report is referred to the state Department of Health, which passes the information to the Secretary of Health Surveillance in Ministry of Health. | | The study pointed out that violence against children is rooted in the Brazilian family culture. The most common forms of violence against children in Brazil are classified as physical abuse, sexual abuse, abandonment / neglect and psychological abuse. |
| Giacomello, K. J., (2011) | | | Brazil | NR | | To understand institutionalized children victims of violence through therapeutic play sessions. | Qualitative study design. Participants were three sheltered children of preschool age, one boy and two girls. | The children interviewed expressed through the performance of Disney characters how was the relationship with their families, how is their living in the shelter. | NR | | | Limitations are related to the study design and the focus of the study only in the children sequelae | | | | The study showed that while children play, they bring common themes of life such as education, family relationships and many other people roles that are represented in their culture. The study showed that the therapeutic playing is beneficial to allow them to express their feelings. In addition, the study also showed that the living of institutionalized children is different from that designed and recommended by the Child and Adolescent Statute given that these children have to take the responsibility of their own care. | |  |
| Gomes, R., Minayo, M. C. S., Assis, S. G., Njaine, K., Schenker, M. | | | Brazil | NR | | To analyzes the success of Brazilian experiences engaged in prevention of violence as well as their limitations, and seeks to show ways for a solution of the problem | Qualitative study design. National literature was consulted in order to identify nine Brazilian "experiences" or "organizations" that deal with violence. (Note: services were not linked, but selected as sites for study) In addition, qualitative interviews were performed with professionals that work in these areas. | Violence preventions includes provision of services such as sports in poor communities in order to maintain children and teenagers distant from urban violence; raising awareness of population in regards racial discrimination. Regarding violence against children and teenagers, the Sentinel Project and the Circle of the World are the only ones focused in the support of victims of violence. The Sentinel Project offers psychotherapeutic care to victims of sexual and domestic abuse and the Circle of World works with children in vulnerable situation or victim of sexual abuse through activities such as theater performance, painting, etc. | NR | | | Although most of the experiences were considered successful separately, challenges are related to the lack of financial, human and material resources which limits the expansion of this success to other parts of the country. Regarding the Sentinel Program and the Circle of the Everyone program, Challenges are related to provide a multidisciplinary assistance to children and teenagers victims of violence. | | | | NR | | NR |
| Granville-Garcia, A. F.,   (2008). | | | Brazil | NR | | To verify the perception and responsibility of dentists in relation to child abuse | Cross-sectional study design. Fifth four dentist surgeons were interviewed. | NR | In general, dentists would report child abuse (96%); the Guardian Council (63%) and the Childhood and Adolescence Court House (22%) were the most cited organizations for child protection. | | | Most dentists do not know how to document child abuse case (61%) nor received such information during their education at the dentistry school (93%) | | | | The study showed that most of the participants interviewed (87%) reported having knowledge of child abuse and considered themselves capable of diagnosis (65%). However, few professionals mentioned oral lesions as evidence of child abuse (4%). | | The study highlighted the need to inform dentists of their legal and ethical responsibility and obligations in cases of child and adolescent abuse. |
| Habigzang, L. F., | | | Brazil | NR | | To identify the measures adopted by the social and emotional support network of children and  adolescents after disclosure of sexual abuse. | Cross-sectional. Participants were 40 girls aged 9 to 16 years old, victims of sexual violence. | NR | Thirty five percent of the girls were referred to shelters and the rest remained living with the family. The Tutelary Council refers the victims to the justice system process but not for healthcare assistance, unless there is evidence of the severity of the aggression. | | | Nurses revealed that they do not report the cases for fear reprisal by the perpetrator. The nurses interviewed also revealed to let social workers and psychologist takeover of the cases | | | | The abuser was responsible for the victim care in 47.5% of cases and 42.5% of the offenders had sexually or physically victimized others. Results also showed that 42.5% girls revealed the abuse to parents and 12.5% to another family. Most of the people that heard from the children believed in the revelation of the abuse situation (92.5%), however 10% of these did not adopt any protective attitude for the children. The security measures adopted by families were the complaint and the removal of the attackers. However, in 32.5% of cases the family did not make a formal complaint of violence to protection agencies, which maintained a vulnerability for the child's situation. | | According to this study, when a formal report is done, the victim is not necessarily or immediately removed from the place in which the aggressor is. Until that actual evidence be found, the Tutelary Council only recommends the removal of the child or teenage victims of violence but this process can take weeks or months before the justice system take over the case. Thus, children and teenagers are kept in a vulnerable situation for more abuse. |
| Ramos, M. L., Cabrera O., Silva, A. L. (2011) | | | Brazil | NR | | To investigate how professionals with the Family Health Strategy act when confronted with situations of domestic violence against children | Qualitative study design. Data were collected through semi-structured interviews performed with twenty-one professionals who works in five primary health care units located in Sao Paulo | NR | Professionals reported to refer the victimized child to the Tutelary Council. | | | The study findings suggest that although the professionals working at the Primary Care clinics do take actions to report and refer victims to the Tutelary Council, they reported to feel unprotected and unprepared to assist the demands of domestic violence against children. Intersectionality, integrality and problem-solving capacity were present in the interviewees' discourse, showing that there are gaps that generate suffering on these professionals and requires concerns by the Ministry of Health and competent agencies. | | | | The study found four thematic categories: identification of domestic violence; types of domestic violence; assistance difficulties in domestic violence situations; and professionals' actions in domestic violence situations. Identification happens during visits made by professionals in the community, particularly the Community Health Agents, and is corroborated in clinical consultations through observation and physical examination of the child. | | The study is informative and was considered to be included |
| Souza, K. C., (2010) | | | Brazil | NR | | To evaluate the relevance and the level of knowledge of dentists, the Basic Health Units, in Joao Pessoa, PB, in regards VAC. | Cross-sectional study design. | NR | NR | | | No information was provided in terms of legal procedures taken to report the cases. | | | This study showed that 94.7% of the interviewed demonstrated to have basic knowledge in regards VAC and physical violence was the most cited by the dentists. 21% of the interviewed reported to have found cases of sexual violence in their practice at the UBS and 10.5% experienced cases of neglect related to lack of hygiene. 15.8% reported to have found cases of lack of parental attention and 21 % of the interviewed never experienced cases of VAC. The remained percentages were associated to experiences of the dentists with cases of physical violence. During clinical exam, 10.7% of the dentists reported have found contusions. 3.6% scratches, 21.4% edemas, 10.7% burnings, 14.2% identified by the children behavior, 7.2% reported that the children develop some mental disorder, and 25% identified by having the children with dirt and unkempt. | | | NR |
| Azevedo, M. S. et al 2012 | | | Brazil | NR  (Ethical approval was in probably 2009) | | To assess the attitudes and perceptions of dentists regarding child abuse, and to investigate professional characteristics associated with the identification of suspected child abuse. | Cross-sectional study using a self-administered questionnaire provided to dentists (N=276) practicing in the city Pelotas. | NR | NR | | | Among those who did suspect, 76.0% did not report the case to legal authorities. The low number of suspicious cases may also be attributed to a lack of dentists’ awareness about dental neglect, the most frequent type of abuse that may be seen in a dental office. | | | | Most dentists believed they could detect cases of child abuse (78.7%). When asked if they had already suspected child abuse, 85.7% reported that this had never occurred. Among those who did suspect, 76.0% did not report the case to legal authorities. The low number of suspicious cases may also be attributed to a lack of dentists’ awareness about dental neglect, the most frequent type of abuse that may be seen in a dental office. Adequate qualification of dental professionals is required for the clinical treatment of the victim, as well as for the early detection of the aggression and notification of legal authorities. Authors believe that it is necessary that dental professionals receive interdisciplinary training to enhance their ability to care for and protect children. Their argument is based on the assumption that interdisciplinary approach may help in eliminating the confusion, and poor decision-making caused by professionals unprepared to interact with one another. | | Child abuse has been included in the curriculum framework of Brazilian dental schools to qualify dentists (and future pediatric dentists) in the diagnosis, treatment, and official reporting of these cases. In the local dental school, the theme is addressed in the discipline of Pediatric Dentistry. |
| Angelo, M. et al 2013 | | | Brazil | NR  (Ethical approval was probably in 2009). | | The purpose of this study was to understand the experience of nurses in their care of child victims of domestic violence, using the phenomenological social approach by Alfred Schütz. | Qualitative study with semi-structured interviews with nurses (N=15) who work in emergency units, intensive care and pediatric inpatient units, about their care of child victims of domestic violence, using the phenomenological social approach by Alfred Schütz, to understand their experience. | NR | NR | | | Nurses not always have autonomy in the institution where they work to perform their role and move forward with the report. Therefore, it is not enough to identify and confirm the violence, because there are still institutions that insist on not report violence against children. | | | | This article mentions the need for institutional care protocols to minimize errors in the identification of cases of violence against children and the existence of a supporting network for professionals taking care of patients in this situation. This network could help professional in the identification, care, assessment and reference of cases of child victim of family violence, as well as to handle their own feelings of emotional ambivalence, and identify the family vulnerability. | | NR |
| Cabelus, N 2007 | | | Colombia | 2005 | | Conduct a forensic nursing assessment on the ICITAP sex crimes project in Bogota, Colombia | Forensic nursing assessment/consultation | All-in-one center: combines healthcare and law enforcement in one building | NR | | | Survivors and cross paths with perpetrator in the same building; facilities too small to accommodate clients; few number of legal investigators results in referral to psychologists who act as investigators; victims of sexual abuse cannot access prompt medical care or to the Institute. of Legal Medicine for forensics examinations due to lack of transportation in more remote areas; | | | | NR | | NR |
| McNaughton Reyes, 2012 | | | El Salvador | NR | | To examine health sector guidelines for the treatment of sexual violence in El Salvador, Guatemala, Honduras and Nicaragua, and assess services in 34 private- and public-sector facilities in the four countries. | Evaluation of medical facilities for adherence to the 2007 SV guidelines; survey of med professionals and med rec review of survivors; N=6 facilities | Provides explicit instructions for examination of victims; treatment of injuries; pregnancy testing and providing emergency con- traception; testing and treatment for STIs/HIV; and psychological support. Facilities have analgesics and antibiotics but lack HBV vaccine, HIV PEP, and pregnancy tests | Require legal reporting of VAC/VAW with no opt-out of reporting; Guidelines recommend referral, but only 62% of the 34 facilities assessed indicated they provided referral or on-site psychosocial support services and counselling, and only 26% provided on-site services or referral for legal aid; all of these were private sector NGOs | | | Guidelines not well /widely disseminated | | | | Guidelines revised in 2010 | |  |
| Coope, CM 2006 | | | Guatemala | 2001 | | To delineate the definition, common forms, and perceived risk factors con- tributing to child neglect in Guatemala from the perspective of different stakeholders and identify the challenges faced by child protection practitioners in identifying children at risk of neglect within the context of Guatemala. | Qualitative research conducted in Guatemala City, including: semi-structured interviews (SSI) with key stakeholders involved in child neglect (n = 9); semi-structured interviews with professionals working in the child protection services (n = 14); focus group discussions (FGDs) with professionals, voluntary workers and children (n = 60), and the group consensus method with key informants (KI, n = 10). Research objectives: (1) delineate the definition, common forms, and perceived risk factors contributing to child neglect in Guatemala from the perspective of different stakeholders and (2) identify the challenges faced by child protection practitioners in identifying children at risk of neglect within the context of Guatemala. | NR | NR | | | Legal recognition of child neglect: lack of legislation recognizing child neglect as a legitimate form of child abuse was a major obstacle to improving the detection and services for neglected children in Guatemala. Service providers agreed that children in Guatemala are afforded a very low status and that the concept of a child as an individual with rights is not commonly accepted. Options for care: the scarcity of service options for children who were maltreated was highlighted. Going into a care home was described as the only option, other than being placed with another family member. A care home placement was considered a poor response to neglect. | | | | NR | | Majority of study focuses on assessment of perceived risk factors related to VAC and neglect |
| McNaughton Reyes, 2012 (Same as above) | | | Guatemala | NR | | To examine health sector guidelines for the treatment of sexual violence in El Salvador, Guatemala, Honduras and Nicaragua, and assess services in 34 private- and public-sector facilities in the four countries. | Evaluation of medical facilities for adherence to the 2006 SV guidelines, survey of med professionals and med rec review of survivors; N=7 facilities | Emergency contraception; STI diagnosis / prevention/treatment provided. The Forensic Medicine Department in the Public Ministry is responsible for conduct of the forensic examination, analysis of specimens, and producing medico-legal reports for judiciary procedures. | Mandatory reporting of child SV to legal authorities without exception for provision of patient-provider confidentiality. Provide referral to legal/justice institutions and referral to support groups. | | | No specific health sector guidelines on care for sexual violence, except for 1997 guidelines on health services for adolescents which included provision of services for adolescent victims of SV. | | | | NR | | NR |
| McNaughton Reyes, 2012 | | | Honduras | NR | | To examine health sector guidelines for the treatment of sexual violence in El Salvador, Guatemala, Honduras and Nicaragua, and assess services in 34 private- and public-sector facilities in the four countries. | Evaluation of medical facilities for adherence to the 2007 SV guidelines; survey of med professionals and med rec review of survivors; N=7 facilities | Provides explicit instructions for examination of victims; treatment of injuries; pregnancy testing and providing emergency con- traception; testing and treatment for STIs/HIV; and psychological support. Facilities have analgesics and antibiotics but lack HBV vaccine, HIV PEP, and pregnancy tests | Require legal reporting of VAC/VAW with no opt-out of reporting; Guidelines recommend referral, but only 62% of the 34 facilities assessed indicated they provided referral or on-site psychosocial support services and counselling, and only 26% provided on-site services or referral for legal aid; all of these were private sector NGOs | | | Guidelines not well /widely disseminated | | | | Guidelines revised in 2010 | |  |
| Armenta, 1999 | | | Mexico | NR | | To determine important predictors of discretion in detecting and reporting VAC among health professionals. | Cross-sectional; N=269 medical professional | NR | NR | | | Predictors of discretion in detection and reporting of VAC: young age, the lack of training in detecting and handling child abuse cases, and having reasons for being discretional. | | | | NR | | NR |
| De Los Angeles Rodriguez-Bolanos, R. 2005 | | | Nicaragua | | 2003 | To assess the knowledge and attitudes of health care personnel towards the identification and referral of gender-based violence victims (GBV). | Cross-sectional study conducted among healthcare professionals (physicians, nurses and nursing technical aids; N=213), in 5 of the 17 Local Systems of Integral Attention (SILAIS) from the Integral Program of Attention for Women, Children and Adolescence (AIMNA) in the primary level of attention in MINSA. Measured attitudes and knowledge of GBV | NR | NR | | | NR | | | | Study found general consensus on opposition to violence (76%). Barriers to providing medical care included lack of training on the subject (59.9%), fear of getting involved in legal issues (52.6%), and the concept that violence is a private affair and not a social one (50.7%), | |  |
| McNaughton Reyes, 2012 (Same as above) | | | Nicaragua | | NR | To examine health sector guidelines for the treatment of sexual violence in El Salvador, Guatemala, Honduras and Nicaragua, and assess services in 34 private- and public-sector facilities in the four countries. | Evaluation of medical facilities for adherence to the 2006 SV guidelines, survey of med professionals and med rec review of survivors; N=15 facilities | Selectively screen for violence against women/children, if provider suspects violence ; General comment that physicians must attend to patient's physical and psychologic needs; Facilities have analgesics and antibiotics but lack HBV vaccine, HIV PEP, and pregnancy tests | Require legal reporting of VAC with no opt-out of reporting. Guidelines recommend referral, but only 62% of the 34 facilities assessed indicated they provided referral or on-site psychosocial support services and counselling, and only 26% provided on-site services or referral for legal aid; all of these were private sector NGOs | | | Guidelines not well /widely disseminated. Guidelines also recommend follow-up of patient but no specific implementation plans | | | | Guidelines revised in 2009 | | NR |
| Nunez, 2008 | | Venezuela | | 2004 | | To understand conceptions held by health professionals with regards to violence within the family against children and adolescents. | Review of data registered in Forensic Medicine in Aragua State (N=487 victim - 421 of whom were <19yrs) | Forensic exam | Only 20% of the cases of child abuse were reported by the person conducting the physical exam; only 2.46% were offered a referral to a psychiatric exam; | | | Gaps in linkages to other services (reporting, psychosocial) | | | | NR | | The study provides data across age groups (not disaggregated for children & adolescents, except for age of victimization). Study focuses on analysis of the types of violence, perpetrator, etc., but less detail in types of services offered, etc. |

| **Table S2.** Studies describing exposure to training or developed and tested curriculum to train health providers on addressing VAC (N=19) | | | | | | | | | | | | | | | | | | | | | | | | |  |  |  |  |
| --- | --- | --- | --- | --- | --- | --- | --- | --- | --- | --- | --- | --- | --- | --- | --- | --- | --- | --- | --- | --- | --- | --- | --- | --- | --- | --- | --- | --- |
| **Study (Author, year pub)** | **Country** | | **Yr(s) of study** | | **Study objective** | **Target profession/degree for training** | **Training content** | | **Duration of training** | | **Method of evaluation** | | | **Identified gaps/limitations** | | | | **Results / Impact** | | | | | **Any other summary of study** | |  |  |  |  |
| de Silva, LM 2012 | Brazil | 2009 | | | To identify the work developed by the Judiciary to prevent sexual violence against children and adolescents within the family. | Members from the Courts for Crimes against children and Adolescents (CCCAs) inc judges, assistants, judicial technicians, analysts, interviews and FGDs (N=17) | NR | | NR | | NR | | | Study discussed past exposure to training/education: 5/17 indicated that sexual violence against children was taught in undergrad program; 11/17 indicated that they attended a lecture or course on SV against children | | | | NR | | | | Qualitative study with members from the CCCAs inc. judges, assistants, judicial technicians, analysts, interviews and FGDs (N=17) | | | |  |  |  |
| Brum, C. R. S. et al, 2013 | Brazil | 2009 | | | Objective: To evaluate the beliefs and possible changes resulted from intervention, in health professionals in relation to domestic violence in Primary Health Care services through an intervention methodology focused on the training of these professionals to work in a preventive manner towards family violence issues. | Population: Primary Health Care providers (N=65). | Training about practices on the prevention of substance abuse and domestic violence. Regarding domestic violence, the training sought to develop conceptual, communication, interpersonal and political skills of the professionals in relation to the impact of violence on health; the actions of prevention; the care of the victims and the management actions. The content of the training was based on guidance materials for health professionals, prepared and published by the Ministry of Health (Brazil, 2002a, b). | Three modules of about 4 hours | | | The instrument was applied before and after a conceptual training on domestic violence, in order to analyze the beliefs and possible changes from this type of intervention. Besides other socio demographic information the questionnaire had questions concerning previous participation in some kind of training. | | For the evaluation it was used a questionnaire about sociodemographic aspects, which also included items concerning previous participation in some kind of training; and other questionnaire that consists of a first part that evaluates the practices of prevention of domestic violence, and a second part, which analyzes the professional's beliefs regarding the situation. | | | | | The content and form of presentation of training touched on relevant theoretical aspects as well as the importance of their contribution to the care of patients involved in this type of situation. - After training there was an increase from 13.8% to 27.7% of the number of professionals who reported knowing enough about the causes of problems related to violence  - Increase from 24.6% to 43.1% in the number of professionals who mentioned that they could properly orient their patients about domestic violence and its effects.  - Decrease from 24.6% to 13.8% in the number of professionals who agreed with the statement that they had not much to offer to patients victims of domestic violence.  -Decrease from 71.9% to 46.9% in the number of professionals who said they were too busy with other health problems. -Decrease from 47.7% to 30.8% in the number of professionals who reported not knowing how to effectively address patients to reduce domestic violence. -Decrease from 24.2% to 21.0% in the number of professionals who agreed that there was not enough time to counsel patients about domestic violence. | | | The Family Health Program (PSF) is in prime position to implement preventive measures of domestic violence, due to its proximity to the local community, intersectoral action, the establishment of partner-ships, constant dialogue with different sectors, and primarily, its responsibility in health promotion. | | | | | | | |
| Cardoso, F. A. et al 2011 | Brazil | 2009 | | | The goal was to conduct a training workshop for 22 community health workers (ACS per its acronym in Portuguese) in order to enlighten them about their role in community work and raise their self-esteem. | Target population of the activity: Community health workers (N=22) | The use of active methodology facilitated learning. Chosen themes included: child health; elderly health; and violence against children, women and elderly; plus questions about ACS's assign-ments, professional ethics, communication and teamwork. A social worker provided information to the group on how to act when witnessing a case of violence and the different types of notification. She also emphasized that ACS must have knowledge of family history and the probable kind of violence that is taking place to find out when and how to make a notification. | | NR | | | 20 hours distributed in five weekly meetings, of which 16 hours were in-person and 4 hours were completed with texts that the participants were to read at home. | The authors used the problematization (active) method for the activities and did not perform any type of performance evaluation. However, to evaluate the activities, at the end of each section, it was carried out an assessment of the day's activities by the participants, through the use of colored cards with drawn faces. The green card represented a positive assessment about the day, yellow card represented a regular day, while the red card represented a bad day. Each participant was asked to choose the card that best indicated their assessment of the activities, and present to the rest of the group giving an explanation about their assessment. Participants reported that they often see the signs of violence in families, but do not know how to act, how to support victims and end up silencing. | | | | | | After two months, in order to check what kind of impact the educational workshop had on participants' performance, it was delivered a questionnaire with open questions about changes perceived by them in relation to their work. In this evaluation, almost all participants (98%) pointed out that there were changes in their work routine. They said they were applying new techniques for addressing issues and assisting families.  Many said they were feeling more valued and thinking more collectively, which had eased the way they did their activities. | The training generally provided to the community health agents proves to be insufficient and deficient, not preparing them properly to act on the problems they encountered in the exercise of their comm-unity work. | | | | | | |  |  |
| Gomes, L. S. P. (2011) | Brazil | 2010 | | | To verify the perception of students in 4th and 5th year of Dentistry of State University of Paraiba about child abuse. | Cross-sectional study design. Participants were undergraduate students in dentistry of the University of Paraiba. | The training is mainly focused in clinical assessment to identify VAC and ethical aspects involving the theme VAC. | | The course takes one semester and it is offered for students in their 4th year, before their clinical internship. | | NR | | | NR | | | | The study showed that the majority of the participants reported that is the responsibility of the dentist to diagnose cases of child abuse (91.2%). Similarly, the majority of respondents reported that they would be able to identify cases of child abuse (94.5%) and regarding the most mouth and body signals cited, edema / hematoma, corresponded to 31.4% and 70, 9%, respectively, of the all signals mentioned. | | | | | The study suggested that theme of child abuse should be approached in a multidisciplinary way, in disciplines such as Pediatric Dentistry, Surgery, Trauma Maxillo-Facial and the Preventive and Social Dentistry, in order to train future healthcare professionals to recognize and report cases of VAC. As result of this study, the theme of child abuse was included in the curriculum of the courses of dentistry in the University of Paraiba. | |  |  |  |  |
| Koifman, L., (2012) | Brazil | 2010 | | | To survey the understand the theme Violence against Children in the medical school of the Universidade Federal Fluminense (UFF) and to analyze the perceptions of the students, teachers and medical residents regarding their ability to deal with VAC. | Cross-sectional study design. Participants were 120 med students, 10 residents and 10 professors who completed self-assessment questionnaires | NR | | NR | | NR | | | The article revealed not only the lack of training of medical students in regards VAC during the course but also de lack of sense of responsibility of the faculty in approach this theme with the students. | | | | The study showed that most of the students (44%) reported to do not to feel prepared to deal with the issue in the work setting, but believe they will be by the end of the course. There was a decrease across the years of internship (73%, 50%, 10%, respectively); indicating that as they approach graduation, they lose hope in becoming prepared to deal with the issue. There was an increase (8% 33%, 45%) of students who do not believe the end of the course will be prepared. The majority of students during internship (30%) reported feeling prepared to identify cases of VAC but not to deal with the case. However, many teachers believe that it is not their responsibility to teach the students about this topic. Five out ten medical residents reported to have had experiences with VAC. From these, one reported feeling secure with reporting the case but not to deal with it. The remaining medical residents reported feeling secure in handling the cases, however the procedures they followed were different from what was recommended by experts (authors did not mention what are the standard recommendations). | | | | | | | | | |  |
| Moeira, G (2013) | Brazil | 2012 | | | To analyze training and knowledge of professionals in the Family Health Team on reporting the mistreatment of children and adolescents | Health professionals in Ceara State, NE Brazil - physicians (N=9), nurses (N=26), dentists (n=16) who work in Family Health Strategy (N=51 totals); | Developed in response to the Child and Adolescent Act (Estatuto da Criança e do Adolescente - ECA), which regulates mandated reporting of any suspected or confirmed abuse case by professionals in health care and education (art. 13), establishing a penalty for those who fail to do so (art. 245), and Ordinance n. 1.968/2001, from the Brazilian Ministry of Health, which established mandatory reporting of abuse against children and adolescents, treated at the Brazilian public Unified Health System (Sistema Único de Saúde – SUS). | | | NR | Cross-sectional survey of physicians (N=9), nurses (N=26), dentists (n=16) who work in Family Health Strategy (N=51 totals of 104 in the population); | | | Overall gap in exposure to training/education on VAC and reporting | | | The majority of professionals had not participated in violence against children and adolescents training (86.3%); but majority who knew the Child and Adolescent Statute (90.2%), and how to notify mistreatment (62.7%). Most interviewees said that the health unit had the form (70.5%), and they knew where to refer victims to (82.3%). Only 37.2% had already identified some case and, among them, 60.0% reported the occurrences. There was a significant association (p=0.035) between the act of notifying and past participation in training on the subject of reporting VAC. | | | | | | |  |  |  |  |  |
| Bannwart, T. H. 2011 | Brazil | 2007 - 2008 | | | To describe and analyze the difficulties found in identifying and notifying abuse against children and adolescents in the opinion of pediatricians. | This is a qualitative study involving eight pediatricians from a charity hospital in a midsized municipality of the state of São Paulo. | Pediatricians | | NR | | NR | | | The Child Protective Service is responsible for protecting children and professionals at the same time, avoiding professional exposure; and the police are responsible for investigating the case. However, the medical culture leads doctors to treat the phenomenon of abuse as if it was a disease, a diagnosis to be confirmed by tests, limiting it to the biological aspects when it is a social problem instead. The main difficulties noted by the pediatricians were disbelief in the effectiveness of the Child Support Service, non-existence or inadequate training on child-abuse issues by the pediatricians, and personal limitations. | The professionals’ lack of training to deal with victims of violence (often identified in their statements and in the questions they made) might be caused by their lack of knowledge on how to proceed in such cases. In Brazil, the shortage of regulations setting technical procedures, the absence of legal mechanisms to protect those responsible for reporting the abuse, the failure to identify violence in health care facilities, and the lack of respect for professional secrecy are considered barriers for reporting. | | | | | | | | Professionals thought that in order to report the case, confirmation of the suspected abuse is needed, which contradicts the law.  Only one participant reported he took a theoretical course in a Department of Legal Medicine and another participant reported cases he/she saw during his/her training, that is, he/she apparently expanded the concept during the residence period, learning about the topic during clinical practice. | |  |  |  |  |
| Carvalho, L. M. F. et al 2013 | Brazil | 2009 (Approval of Ethics Committee) | | | Evaluate the knowledge, of the dental professionals (N=80) in public and private services of the city of Guaratingueta, Brazil, on identification procedures when dealing with domestic violence against children, women and elderly. | Descriptive study in which it was used a structured questionnaire specifically designed for this study, containing closed questions on domestic violence. Items analyzed: experience of professionals to identify domestic violence at three levels (children, women and elderly); the professionals behavior in situations of domestic violence; and teaching domestic violence in graduation courses. | NR | | NR | | NR | | | NR | Most respondents indicated having received no information during the undergraduate course and / or post-graduation on the topic domestic violence. And in that sense, most do not feel prepared to identify the cases of abuse. The authors noted that there is no standardization of care by the professionals analyzed in case of confirmation of domestic violence against children, as 60% of professionals in the public and 50% private network would communicate the fact to the competent authority, but do not truly know who to notify. | | | | | | | | Health workers are afraid to notify suspected cases of abuse because of fear of losing patients, lack of trust in the protection service, they are afraid to deal with parents when children are involved, lack of knowledge about the responsibility to notify. Some professionals believe also that the judicial system will do nothing regarding the case and therefore, the notification to the competent authorities would not bring any positive results. | |  |  |  |  |
| Lidchi, (2007) | Brazil | NR | | | To design a training program in child protection to respond to the particular challenges to effective practice posed by the local environment and to address obstacles to its achievement in the existing child protection system | Local agencies and institutions in health, education, social services (governmental and NGO); 2 participants per group, in Rio de Janeiro, Minas Gerais, Maranhao, and Pernambuco | IPSCAN and CEIIAS training that aimed at fostering an integrated approach and using concerns/beliefs of providers to identify and resolve challenges related to the following framework: Legal provisions, Beliefs and knowledge, Identification, Services activation, Service response, Coordination, Review. 1) Sensibilização (raising consciousness) addressed beliefs and knowledge, which provided definitions, presentation material and identified levels of intervention in keeping with a public health approach. 2) Capacitação (capacity building) focused on skills specific to each sector, including clinical, psychological, and forensic areas. 3) *Trabalho de Rede* (working on the network) focused on developments in inter-sector networks aimed at overcoming fragmentation and in reviewing and improving the effectiveness of the system. | | NR | | Evaluated participants in Minas Gerais (N=120; 63 with complete data) | | | Identified heterogeneous use of mandatory reporting across groups; lack of central organization in Brazil for coordination, collection and archiving of reports. Mandatory reporting forms are produced at the state and municipal levels and will be joined by general violence reporting forms that include women, children, elderly. Providers indicated that they did not report cases to to *conselhos tutelares* (who are legally responsible for assessing children) and reported directly to agencies due to lack of training or perception of ineffective *coselhos tutelare*s. Providers avoided mandatory reporting because of risks and concern about child welfare post-notification. Some avoided reporting b/c of risk to the provider.  Results of survey and provider experiences/beliefs were used to further inform the development of the training curriculum. | NR (did not evaluate results from training) | | | | | | | | Used training of trainers approach- participants expected to teach others | |  |  |  |  |
| Bourroul, M.L.M. et al 2008 | Brazil | NR | | | The objective of this study was to understand the perceptions of pediatricians regarding domestic violence against children and adolescents (DVCA). | Medical and social qualitative research, performed in two interrelated and dependent stages. The first stage was conducted with 67 medical residents of the second year of pediatric residency offered in nine programs in the city of São Paulo. The 67 participants were presented with a "clinical case" and then they were asked to answer a questionnaire, through which they should write diagnostic hypotheses, describe referrals, and further explanation of the case itself and the difficulties of the care provision, learning opportunities and the role of the pediatrician in relation to DVCA. In the second stage, nine in-depth interviews were conducted. | Medical residents of the second year of pediatric residency | | NR | | NR | | | NR | | Participants indicate that the guidance given in manuals, protocols and classes are insufficient. Most of the residents recognize personal difficulties (prejudgment, anguish, embarrassment, indifference, denial, rejection, alienation, fear of the perpetrator), more than half cited technical difficulties (how to assess the case, how to diagnose, how to break the covenant of silence and omissions), and a minority described the families involved in this situation as "hostile". The residents who reported some form of fear also considered their own training as insufficient. Many residency programs create mechanisms that avoid the resident conduct on cases of violence, putting these cases as a responsibility solely of the social workers. The participants also showed lack of commitment and resistance towards notification, which was justified by negligence or difficulty of involvement with the court. | | | | | | | Regarding the role of the Child Protection Agency, the interviews showed both anger at the mismanagement of certain cases, and disagreement in relation to the basic proposal of re-inclusion of the child in the family.   The results of the interviews also showed that the structures of the programs do not involve or do not prioritize outpatient care, and even less, the follow-up of cases with suspicion of violence, which results in an incomplete approach and therefore a limitation of the training. | |  |  |  |  |
| Cavalcant, A 2009 | Brazil | NR | | | This study evaluated the perceptions and knowledge of pediatricians and pediatric dentists (PD) from the city of Campina Grande, PB, Brazil, about child abuse. | Descriptive study in which it was used a structured questionnaire specifically designed for this study, containing closed questions on domestic violence. The sample was composed of 63 professionals (28 pediatricians and 35 PDs) A questionnaire was used for data collection. | NR | | NR | | NR | | | Most professionals (65%) declared they did not receive information about child abuse during college. When asked about the institution to which the notifications of child abuse should be made a percentage of 4.7% reported they did not know, | | Most professionals surveyed (93% pediatricians and dentists 91.4%) emphasized the need for the theme child maltreatment be present in the undergraduate curriculum of both courses, medicine and dentistry, respectively. A significant percentage of pediatricians (78.6%) and only 34.3% of dentists had already suspected the occurrence of abuse involving children and adolescents. When asked in relation to the adoption of conduct in suspected cases, all the pediatricians (100%) and 83.3% of dentists reported having adopted an attitude, the most frequent attitude was notification to the Child Protection Agency (62.5%) and a talk with parents (31,2%). All pediatricians (100%) and 88.6% of the PDs considered themselves able to identify cases of physical violence in their working environment.  The presence of injuries was the most frequent clinical evidence for the suspicion of abuse (67.7%). Most professionals (68.2%) affirmed to know their obligation to report cases of violence against children, but pediatricians had better knowledge of the compulsory notification than dentists (92.8% versus 48.6%). When asked about the institution to which the notifications of child abuse should be made, the Child Protection Agency was the most mentioned (41.3%), followed by the Curator of Children and Youth (35%), Police Station (3.2%) and Other Bodies (ABRAPIA / SOS) with 3.1% .A percentage of 4.7% reported they did not know, while 12.7% did not answer this question. | | | | | | | The authors state that it is necessary to conduct training courses on the subject of child abuse for pediatricians and dentists, since these professionals are responsible for providing care to children from an early age through adolescence. Additionally, changes in the curriculum to add specific content directed to the diagnosis of domestic violence become indispensable in order to best qualify the future professionals. | |  |  |  |  |
| Luna, G. L. M. et al 2010 | Brazil | NR | | | The article analyzes the process of notification of violence against children and adolescents carried by doctors, nurses and dentists part of the family health teams in Fortaleza, Ceará State. | Quantitative approach, it is a cross-sectional study. Participants: doctors, nurses and surgeon dentists registered in the family health teams (N=359). | NR | | NR | | NR | | | A total of 40.2% of the doctors, 34.8% of the nurses and 44.6% of the dentists do not trust the protection agencies. A total of 59.8% of doctors, 50.7% of nurses and 63% of dentists stated the subject "violence against children and adolescents" is not discussed in the unit where they work. | A total of 66.3% of the doctors, 68.5% of the nurses and 71.8% of the dentists that participated in the research stated they had never participated in training related to violence against children and adolescents. A total of 10.4% of the doctors, 7.3% of the nurses and 14.7% of the dentists not interested in participating in training on this subject. A total of 37.7% of doctors, 35.6% of nurses and 68.5% of dentists stated they didn't know the Child and Adolescent Statute (ECA per its acronym in Portuguese). A total of 49.4% of doctors, 39.5% of nurses and 60.8% of dentists didn't know the notification form and a total of 40.2% of doctors, 25% of nurses and 61.5% of dentists stated they do not know if the unit where they work have the notification form. Among the professionals that had assisted patients victims of violence a total of 55.6% did not notify the cases; 41.5% stated they don't know where to refere the patient to; 62.2% are afraid of legal involvement; 81.6% stated they do not know institutions that give support to the victims. | | | | | | | | The study concludes that the increase of continuing education programs and the expansion of professional support networks may reduce insecurities and increase the number of notifications. | |  |  |  |  |
| Pires, J. M. et al 2005 | Brazil | NR | | | The objective was to determine factors interfering with the reporting of child abuse by pediatricians to children protection services. | Cross sectional observation study. Participants were a random sample of pediatricians from Porto Alegre (N=92) | NR | | NR | | NR | | | NR | A total of 58.70% of the participants presented enough knowledge about violence against children and adolescents; 52.17% did not participate in specific training in this area; 53.26% does not trust the child protection institutions; and 94.57% reported to fear legal sanctions for notifying a case of child abuse. | | | | | | | |  | |  |  |  |  |
| Souza, E. R., (2009) | Brazil | NR | | | To identify the conceptions and suggestions of professors of the medicine and nursing courses about the insertion of the topic intrafamilial violence in the formation of their students | A questionnaire was applied to coordinators of the nursing and medicine disciplines at public and private universities in the cities of Rio de Janeiro and Cuiabá | NR | | NR | | NR | | | This study identified gaps such as discomfort and pre-judgements, between the students and professors in the classroom when this topic is approached, which do not makes them feel able to discuss this thematic. The study suggested that a renovation in the curriculum is urgently necessary in the nursing and medical schools in Brazil. | Many of the participants recognized violence as a health-related problem. The coordinators interviewed informed that 40.7% of the medicine professors and 71.1% of the nursing professors discusses this topic in their classes. The study also identified that the medicine professors insert this topic in their students curriculum by performing visits to institutions of children assistance, meeting groups with people in violence situations and encouraging students to conduct interviews with experienced professionals of the area. The nursing professors prefer resources such as films and videos, seminaries and conferences in order to approach this content. | | | | | | | |  | |  |  |  |  |
| Azevedo, M. S. et al 2012 | Brazil | NR  (Ethical approval was in probably 2009) | | | To assess the attitudes and perceptions of dentists regarding child abuse, and to investigate professional characteristics associated with the identification of suspected child abuse. | Cross-sectional study using a self-administered questionnaire provided to dentists (N=276) practicing in the city Pelotas. | Child abuse has been included in the curriculum framework of Brazilian dental schools to qualify dentists (and future pediatric dentists) in the diagnosis, treatment, and official reporting of these cases. In the local dental school, the theme is addressed in the discipline of Pediatric Dentistry. | | NR | | NR | | | Among those who did suspect, 76.0% did not report the case to legal authorities. The low number of suspicious cases may also be attributed to a lack of dentists’ awareness about dental neglect, the most frequent type of abuse that may be seen in a dental office. | Most dentists believed they could detect cases of child abuse (78.7%). When asked if they had already suspected child abuse, 85.7% reported that this had never occurred. Among those who did suspect, 76.0% did not report the case to legal authorities. The low number of suspicious cases may also be attributed to a lack of dentists’ awareness about dental neglect, the most frequent type of abuse that may be seen in a dental office. Adequate qualification of dental professionals is required for the clinical treatment of the victim, as well as for the early detection of the aggression and notification of legal authorities. Authors believe that it is necessary that dental professionals receive interdisciplinary training to enhance their ability to care for and protect children. Their argument is based on the assumption that interdisciplinary approach may help in eliminating the confusion, and poor decision-making caused by professionals unprepared to interact with one another. | | | | | | | |  | |  |  |  |  |
| Fierro Monti, C 2012 | Chile | NR | | | To describe the knowledge and attitudes regarding VAC among dentists serving 9 health posts in Concepcion, Chile. | Dentists (N=53) | No a training program; study of awareness and history of training/education on VAC among dentists | | NR | | NR | | | Training not common during undergraduate training | 50% of dentists had received info on VAC - most from formal sources, such as conferences; median knowledge score was 6/10; 43% had ever suspected VAC and, of these, 57% had officially reported the case | | | | | | | | Quantitative study to assess awareness of VAC and history of training/education related to VAC; | |  |  |  |  |
| Alzate, DP (2009) | Colombia | NR | | | To develop a a network to understand the situation of child abuse in the region and coordinated approach to this problem among the nursing program and the Law Faculty of Libre University Pereira. | Joint nursing & law | Integrated nursing and law curriculum for VAC; transdisciplinary research; develop educational curriculum intervention for children | | NR | | NR | | | Non-unified definition of VAC in country; | ND | | | | | | | | Proposal - no data on whether implemented | |  |  |  |  |
| Vidal, A (2007) | Puerto Rico | NR | | | To evaluate the knowledge of health professionals about clinical management of sexual aggression in children based on the sexual aggression survivors manual in Puerto Rico. | Physicians (N= 37) , Nurses (N=96) | No training but an evaluation of provider knowledge on management of sexual aggression in children based on the country's manual | | NR | | NR | | | Its not clear how long after the publishing of the protocol this study was conducted. Also, it is not reported how health care workers would have become aware of the protocol | 73%of physicians and 67% of nurses report no training in SA management. 75.9% of the HCW ignored physical signs and symptoms of SA; 24.1% recognized all physical indicators. 66.2% recognized emotional indicators. 82.7% reported that child victims should be referred to an MD or nurse, 94.7% were unaware of steps needed to carry out a crisis intervention, however the majority were aware of the what should be included in the physical exam and where to conduct the exam. | | | | | | | | According to the protocol, all health professionals are supposed to be certified to possess the knowledge and skills necessary to evaluate SA | |  |  |  |  |
| Fernandez, G (2007) | Venezuela | | | 2008 | To make a socioeducative intervention with simulation, diaporames and mental maps that facilitate self-diagnosis of mistreatment among women and children and promote family values. | 60 medical students, 14 medical residents, 2 university docents, 4 nurses, one social worker, one psychologist, and one philosopher | Provides education sessions in three main areas: overview of the situation of family violence (20 sessions of 4hrs each); strategies for improvement and change and to strengthen values between parents and children (25 sessions of 4 hours each), and reflexive learning strategies about changes in family dynamics (Reflexiones de las estrategias de aprendizajes). Education sessions incorporated mental maps, simulations, and diaporamas. | | 20 sessions of 4hrs each | | Pre-post design, little detail provided on the methods of assessment | | | Very little statistical results provided to support claims of improvement | "Improved family functioning, eliminated 95% of cases of neglect, strengthened family values (respect, responsibility, spirituality), improved self-esteem of women and children, and improved school performance by 7%" | | | | | | | |  | |  |  |  |  |

| **Table S3.** Studies describing interventions to address VAC in the health sector (N=20) | | | | | | | | | | | | | |
| --- | --- | --- | --- | --- | --- | --- | --- | --- | --- | --- | --- | --- | --- |
| **Study**  **(Author, year pub)** | **Country** | **Yr(s) of study** | **Study objective** | **Target population** | **Study Design, population** | | **Intervention description** | **Identified gaps/limitations** | | | **Results / Impact** | | **Any other summary of study** |
| Campos, M. A. M. R. et al 2005 | Brazil | 2004 | Implementation of the Integrated Actions Protocol to the Care of Victims of Sexual Violence, involving the areas of Health and Public Safety in the Hospital Complex of Sorocaba - SP. | The target population of this intervention is victims of sexual violence, therefore it includes adults and children. | Epidemiological research, using data collected from the "Notification Form of Immediate Assistance to Person in Situations of Sexual Violence" used by the institution for registration of performed care. It was established the occurrence period per semester, and the months from April to September / 2003, the period prior to the implementation of the Integrated Action Protocol, and the months of October / 2003 to March / 2004, the period after implementation. The final sample was composed of 211 notifications. | | After the joint efforts between Public Safety and Public Health, it was observed that children previously sent to the Forensic Institute for the realization of forensic examination, were not informed about the care provided in the health facility nor received orientation in regard to the sequels prevention program, as determined by Technical Rule issued by the Ministry of Health (Brazil, 2002). In this sense, the Hospital Complex of Sorocaba, in partnership with the Institute of Forensic Medicine, the Public Security Bureau, the Municipal Health Secretariat, the NGO - Women's Center for Integration and a civil society representative, signed in September 2003, the Integrated Actions Protocol (PAI) in regard to care provided to victims of sexual violence. The effectiveness of these partnerships was prompted by the publication in the Official Journal of the State of São Paulo of the Law No. 10,920 of 2001, which obliges the police stations to inform victims of crimes against sexual freedom, about the right to preventive treatment against contamination by HIV. | | NR | | The number of people seeking care was four times greater after the implementation of the integration protocol. The Police and Guardianship Councils referrals increased significantly in the second period, after the implementation of the integration. It was concluded that the integration between the sectors of Health and Public Safety is essential for victims of sexual violence to receive health care as early as possible, with a focus on the prevention of sequels that threaten their physical and emotional integrity. This joint effort enables faster identification of the aggressor, knowledge of the result of anti-HIV serology, and consequently for the victim, reducing the use of antiretroviral chemoprophylaxis. | | The articulation and the formalization of a referral service between health services, police and the Legal Medical Institute is essential for ensuring adequate care. |
| Apostólico, M.  R. et al 2013 | Brazil | 2010 | The present study aimed to identify the limitations and potential of the International Classification of Nursing Practice in Public Health (Classificação Internacional das Práticas de Enfermagem em Saúde Coletiva – CIPESC®) in nursing consultations with children that were victims of domestic violence. | Children that were victims of domestic violence | Qualitative descriptive case study which examined 15 web-based reports on violence completed by primary care nurses from the Curitiba in the state of Paraná. | | The intervention studied was the use of the International Classification of Nursing Practice in Public Health (Classificação Internacional das Práticas de Enfermagem em Saúde Coletiva – CIPESC®) which is a tool that systematizes care and identifies child abuse during nursing consultations. | According to the authors, CIPESC® showed potential, but the diagnoses and interventions presented in the classifications were not fully utilized by the respondents. Participants did not suggest any intervention which aimed the inclusion of the child or family in the Protection Network for Children and Adolescents at risk for violence. In addition, it was identified a disconnection between the CIPESC® diagnoses and the actions recommended by the Network Protection. The CIPESC® Curitiba nomenclature has diagnostics for recognition of violence and interventions for addressing the problem. However, many interventions are diffused among the diagnoses that point to other processes rather than specific diagnoses of violence, hindering the visibility of the problem and making the continuity of care difficult, since the focus is not on specific diagnoses. It is necessary to develop specific diagnostics for situations of violence that address different aspects and needs of the child, the abuser, and the family.  The reports showed worrisome limits regarding the recognition of needs and vulnerabilities involving the phenomenon of violence. In conclusion, it is necessary to contribute to the nomenclature the attributes concerning freedom and autonomy, which are essential for addressing violence, in addition to methods for evidence-based interventions. | | | The nursing consultation, when performed by well-trained professionals, is an important resource to detect cases of violence, given increased proximity with the family and the understanding of family dynamics.  It is emphasized the importance of promoting debates and training of health professionals on the subject violence against children and adolescents, so that they can provide adequate care. The theme of violence should be incorporated in their training so they can learn to deal with this issue, and use their knowledge in their everyday practice. | | In the introduction is mentioned the National Health Policy to Reduce Accidents and Violence (Política Nacional de Saúde para Redução de Acidentes e Violências) and the existence, in Curitiba (city where the study was conducted), of a Protection Network for Children and Adolescents at risk for violence. |
| Cortes, C.,. (2011) | Brazil | 2011 | To describe and analyze the approach of an occupational therapy intervention to violence prevention in youth and assess the impact of activities and resources used on the understanding of the phenomenon and in the development of coping strategies against violence. | Children and teenagers aged 12 to 14 years old from a public school in Minas Gerais - Brazil. | Qualitative study design. Children and teenagers from a public school in Minas Gerais, Brazil. | | The Multifamiliar Group were created in order to offer a therapeutic environment for children victims of violence and its family members to express their feelings through, games, theatrical performances and counselling sections. | Limitation is related to the level of evidence of the study design | | | The participants reported that theatrical performance is a valid strategy to reflect about violence and elaborate coping mechanism against violence | | The study highlights the role of principal and teachers in promote a closer relationship between community, parents and the school in the awareness regarding violence |
| Moreira, T. N. F. et al 2014 | Brazil | 2012 | Objective of the present article is to understand the care strategies developed by Family Health Program Teams to handle domestic violence situations. | Population assisted by the Family Health Program Teams. | A qualitative analysis approach of selected cases identified by the teams as “difficult”, “typical” and “successful” was adopted. Eleven interviews with 27 participants. (N=27) | | Analysis of the flowcharts enabled five types of case management strategies, combined in different ways in each situation studied, to be identified: 1) closeness and support strategies; 2) monitoring and control strategies; 3) health assessment and treatment; 4) articulation in the network; 5) legal/medical (hospitalization or refuge). Team meetings with the participation of a social worker and/or psychologist were indicated as the main spaces for defining care strategies, a way of integrating the visions of different professionals, but also a strategy for protecting the team, a way of sharing responsibilities and recognizing the emotional aspects the situations of violence aroused in the professionals. The family’s socio-relational diagnostic comes to weigh heavily in defining the team’s work, while the knowledge of the psychologist, the social assistants and community health agents took central place. The community health agent’s knowledge of the family and community’s day-to-day life was constantly drawn on. The different case management strategies included medical consultations, laboratory examinations, home visits by community health agents, social workers, nurses and nursing technicians, dentist appointments and individual and family care from social workers, psychological consultations and educational groups with adolescents. | The authors observed limited action on the part of doctors, nurses and nursing technicians in getting close to and managing the situations of violence, despite having contact with the families on various occasions, highlighted as an aspect to be better exploited by the teams. | | | In the cases analyzed, the agents played different roles: they alerted the teams to risks or probable failures of planned actions; functioned as “conflict mediator” between professionals and community residents; and helped the team to choose the best tool (consultation or home visit) to identify possible “allies” in the family of the neighborhood. Monitoring the cases and the effects of the proposed interventions on the families’ day-to-day life was also a role primarily played by community health agents, put into effect through home visits and, often, through information from neighbors and other relatives. The psychologist mainly contributed by participating in meetings and providing specific actions, as well as sharing in the discussion and reflection of the cases. The social worker also acted together with the community health agents in home visits, meetings with other services from the inter-sectorial network and coordinating adolescent groups. Doctors, nurses and nursing technicians were the professional’s whose participation was most discrete in dealing with the violent situations studied. Medical consultations were used specifically to evaluate general state of health and in looking for possible hepatitis and HIV infections. The nurses acted as a link with the team, and also facilitated communication between professionals. | |  |
| Pfeiffer et al., 2011 | Brazil | 2003-2004 | To report different clinical presentation of child physical abuse and propose a method for classifying its severity as applied to a program that provides assistance for children and adolescents under risk in Curitiba, in Southern Brazil. | Children and adolescents | Retrospective study; notified cases of violence against children and adolescents for 2003 (1537) and 2004 (1974). | | Not an intervention but a proposed system for classifying situations general. Classification was based on 4 determinants of morbidity, these determinants were based on frequency of cases from 2001: condition of the victim, the make-up of the family and its attitude to the violence detected, the type and characteristics of aggression and the profile of the aggressor. No Evidence of Violence (0 to 3 points): the s/s presented by the child are not compatible with the characteristics of intentional physical injury, psychological violence, sexual violence or negligence; there is no aggression or aggressor identified and there are well-established bonds of respect, confidence and affection between the child and its guardians. Mild Case (4 to 5 points): s/s compatible with a single episode of physical or psychological aggression, or negligence is detected, with no consequences for the physical or mental health of the victim, and the victim’s guardians are willing to review their aggressive behavior. Moderate Case (6 to 8 points): s/s demonstrate physical and/or psychological violence and/or negligence that could lead to physical or emotional harm and require outpatients-level treatment and specialist follow-up of both victim and aggressors, or which are mild, but repeated. Severe Case (9 to 11 points): cases of sexual abuse or physical violence, psychological violence and/or negligence, with the victim presenting physical or emotional signs of the violence suffered, needing hospital and/or specialist treatment, including mental healthcare, and guardians failing to clearly demonstrate interest in the treatment and/or in changing their aggressive attitudes. Case involving Risk of Death (12 points): cases of sexual abuse perpetrated by guardians or people who cohabit with the victim, physical violence, psychological violence and/or negligence requiring tertiary-level, specialist and emergency treatment, including mental healthcare, with signs of involvement, collaboration and/or impotence in the victim’s nuclear and extended families | The benefits of the classification system are not further developed in this paper; further the data are retrospective - there is no work to show how the classifications could be used with current admits to the ER or current cases of suspected abuse | | | 2003 - 92.6% of cases were domestic, 7.4% extra-familial (similar trend for 2004). Highest incidence was in the 5-9 age group. Majority of notification of aggression were due to negligence; physical violence was more common than sexual and psychological violence. Moderate cases of violence were the most common, followed by severe and mild cases. Classifying the cases allowed for establishing criteria for urgent and emergency cases, and to create flow diagrams | |  |
| da Silva Franzin, 2014 | Brazil | 2004-09 | Identify and analyze cases of child and adolescent abuse and neglect in Curitiba, Brazil | VAC cases entered into surveillance system | The study included all institutional notifications of individual situations of abuse or neglect against children and adolescents between 0 and 18 years old, living in Curitiba, and in contact with services with reporting responsibilities for the period of time under study. In total, 19,316 records were analyzed. This is an exploratory and descriptive study that takes a quantitative approach. Data were collected from the registry of RPCAV, Curitiba, Brazil, considering all records entered in the database between January 2004 and December 2009. The database includes information provided by trained professionals working at schools, clinics, and other institutions that work with children and adolescents who were able to identify confirmed or suspected individual maltreatment situations according to the network protocol and complete a standard notification form. This form is sent to the municipal, regional, or local reference institutions that are responsible for confirming the case, if suspected, and forwarding the case file to the database manager at the Municipal Secretariat of Health. | | The intervention includes the use of the Network for the Protection of Children and Adolescents at Risk for Violence in the city of Curitiba, Brazil (RPCAV) and evaluation of the mandatory reporting/surveillance system. The RPCAV, which is organized by the municipal government, was established in 2000 and is driven by the magnitude of child and adolescent abuse and neglect in the city. The aims of the network include bringing visibility to child and adolescent abuse and neglect; improving the capacity of professionals for integrated and intersectoral work; offering help to victims, their families, and the author of the aggression and neglect through an educational, not punitive, approach to neutralize the conditions that generate maltreatment; controlling relapses; and developing preventive actions with community participation. The network is based on a mandatory reporting system for all forms of abuse and neglect, suspected or confirmed, with legal basis in the Federal Constitution (Article 227) (Brazil, 1988), and the Statute of Children and Adolescents (Articles 13 and 245) (Brazil, 2001a, 2001b). It involves more than 200 municipal public institutions that work with children and adolescents, as well as non-governmental institutions, including Guardian Councils, Public Ministry, hospitals, organized civil society institutions, military and civil police, and the Court of Childhood and Youth. It encourages the reporting of cases of child and adolescent abuse and neglect through an integrated and intersectoral work. | Does not seem to capture data on response and follow-up of survivor. Notifications of physical violence appear to be low and may reflect under-reporting and normative use of physical punishment | | | Captures cases of neglect, physical violence, sexual violence, psychological violence, abandonment, etc. Increases visibility of VAC | |  |
| Habigzang, LF 2009 | Brazil | 2006-08 |  | Adolescent girls (9-16yrs; N=40) who survived sexual abuse | Non-randomized longitudinal study of cognitive behavioral therapy for sexual abuse; intragroup comparison across time points; assess impact among 3 groups according to their respective waiting time: immediately after the abuse was reported, from one to six months after abuse was reported, and more than six months after abuse was reported. | | Group therapy was aimed at reducing symptoms of depression, anxiety, and post-traumatic stress disorder, in addition to aiding in restructuring dysfunctional beliefs in regard to the sexual abuse experience. 16-session group therapy with various activities (Table 1) | Participants maintained perception of being different form other adolescents. | | | Overall decrease in symptoms of depression, child stress, dysfunctional thoughts; PTSD. No difference in impact across when intervention was applied (immediately following victimization or later). | | Small sample size may explain non-significant difference across groups. |
| Celia, S 1993 | Brazil | NR |  | NR | NR | | Vida Centro Humanistico - program that targeted poor population in Northern Porto Alegre. Integrated health education, culture, leisure, citizenship, science, technology, and professional training. Aimed to address social factors, poverty, and undernourishment to prevent violence. Co-managed with 80 CBOs. Strong focus on women, pregnancy, children. | NR | | | NR | | Review of VAC and programs in Brazil, not a study. Very general description of program (supported by UNICEF) |
| Njaine, K. et al 2006 | Brazil | NR | This article aimed to discuss the experience of networks for the protection of people exposed to situations of violence or prevention networks; it also analyzes the possibilities and limitations in the construction of networks for the prevention of violence, seeking to understand the sense of actions and movements carried out in networks. | Network focusing on health, education and social assistance; Network focused in the prosecution service. | The authors present information about two networks for prevention and protection of children and adolescents victims of violence. The authors conducted a case study of two network initiatives in Brazil. For that purpose the authors of this study applied standardized questionnaires containing general information for each network, they also conducted a focus group with professionals from each network, and performed "field observation". | | NR | **Network focusing on health, education and social assistance:** - The network does not have statistical and centralized records describing the types of action and means of protection or prevention performed. However regional coordinators have records which are not systematized.  - The notification form for cases of violence has been used to track cases and help to build a "database on the situation of violence against children and adolescents" in the city. - The practice of evaluating the results of the protective and preventive actions carried out in the network is not yet established, preventing an appropriate appreciation of the results. **Network focused in the prosecution service:** - There is lack of a team specialized in assisting cases of violence in each municipality to offer technical support to other professionals. - The results of the protective and preventive actions coordinated by the Network are not likely to be evaluated, as the program is under implementation. - The networks need greater financial support, less bureaucracy in the flow of information, and greater integration of the institutions responsible for children and adolescents. | | | Network focusing on health, education and social assistance: The structure of this network, though not a hierarchy, is based on a municipal, regional and local coordination, developing coordinated actions among themselves or autonomously. Each institution involved acts in whole or in part of the recognition process, reporting and monitoring of abuse and violence prevention. The participants of the network are trained through seminars, courses, study groups and participation in events organized by various entities. The network has the physical infrastructure of the municipal public service (phones, computers, rooms, fax, vehicles and other resources) .There is no specific budget for its activities. To the extent possible, the necessary resources for making graphic material, training and infrastructure emerge from the solidarity of different organs. Network focused in the prosecution service: The central concept that guides the construction of the Santa Catarina Network is the one of an information system that is able to contribute to strategic data for all the institutions involved on violence affecting children and adolescents. The Network has a management and technical committee composed of officials from different agencies. Training on the topic of violence and awareness of the need for network service is offered to all professionals participating in the launch of the program in each region. Telephones, fax, computers, rooms and vehicles of the institutions participating in the agreement are available to the network. There is no specific funding to maintain the activities of this initiative, which makes it dependent on the resources available on the organizations involved. | |  |
| Dell'Aglio, D. D.(2011) | Brazil | NR | To gather information about the psychological care provided to mothers of children and adolescent victims of sexual abuse and the perpetrators of such violence. | Mothers of children victims of sexual abuse | Qualitative study design. Forty four mothers of children victims of sexual abuse. | | The mothers were randomly assigned to two different groups. The first group received a therapeutic support treatment and the other group received an treatment based on a cognitive-behavioral approach. | The study points out that healthcare services are not fully equipped to assist the aggressor in all their psychosocial needs. | | | The results showed that the group who received cognitive behavioral therapy showed greater decreasing in recidivism rates as compared to the other group. | | Did not provide any statistical finding of this intervention. Little methodological description provided |
| Habigzang, L. F. (2008). | Brazil | NR | To evaluate a group therapy, based on cognitive-behavioral approach to the girls who are victims of sexual abuse. | Ten girls victims of sexual abuse. | Longitudinal study design. Girls victims of sexual abuse | Twenty sections of therapeutic group were performed with girls aged 9 to 13 years old victims of sexual abuse. Coordinated activities were performed in which aimed to restructure thoughts, emotions and dysfunctional behaviors related to sexual violence experience; reduce symptoms of disorder of post-traumatic stress, stress, depression and anxiety; and the learning of self-protection skills to prevent the victims to blame themselves again in the future. The process for the group therapy development was divided into three steps as well its techniques: Step 1 - Psychoeducation (seven sessions); Step 2 - Inoculation Training stress (five sessions); and Step 3 - Prevention of relapse (eight sessions). In addition, four meeting were performed for the non-abusive family members of the participants during the intervention period. | | Limitation is related to the small sample size to generalize the findings. | | | The study showed that depression and anxiety symptoms among the participants were statistically significant reduced in the post test of the intervention as compared to pretest (p<.001). In addition was found a statistically significant increase in the self-confidence of the girls in the post test (Z= 2, 71, p<0,05). | | The therapeutic group demonstrated beneficial outcomes in promote healthy cognitive and behavioral changes in children and teenagers victims of sexual abuse. |
| Gomez, E (2010) | Chile | 2008 | To provide the findings from a multicenter study conducted in Chile, describing features, problems and risk factors for abuse and neglect Children in users treated by the Protectora de la Infancia in eight of their brief intervention programs  for targeted prevention- later referred to as "Viviendo en Familia" | Children & adolescents | Cross-sectional study of children and adolescents (N=591), their parents and families served by eight brief intervention programs (PIB) “Viviendo en Familia”, | Screened cases of VAC to determine level of abuse and whether intervention was needed; evaluated family relationships and environments to assess risk and environmental factors; Children linked to care from following sources: 1) education and health networks (29%) as schools, kindergartens and clinics; 2) demand spontaneous (27%); 3) specialized networks violation of rights (22%) and Courts Family Rights Protection Offices Children and Youth (OPD) and SENAME; 4) networks promotion and local action (19%) and Centers Children and Youth and Community Programs Family Intervention Network partners SENAME; and finally 5) other networks (3%) , but varied by region | | NR | | | NR | Predominantly described types of violence, perpetrators, demographics of victims, family characteristics. Does not describe the types of healthcare provider, referrals, or linkage to services. Does not include perspectives of other actors (managers, professionals, and networks) on the characteristics or program needed to address these problems | |
| Espinosa, 2008 | Colombia | 2004-05 | To develop a domestic violence surveillance system. | Communities in Valle | Evaluation of surveillance system implemented in 21 municipalities for monitoring VAW and VAC | | Surveillance system: This strategy involved standardized information gathering procedures originating from the different data sources and subsequently compiling, validating and digitalizing the information through a common software program. As an outcome, the observatories presented reports providing compiled information and detailed analysis to policy decision makers, stakeholders, and interested community members. In terms of epidemiological surveillance systems (ESS), this project reflects a universal ESS system, i.e. one including all cases occurring within a defined population, with active collection and consolidation procedures. In other words, those in charge of the ESS contact the persons who record the information and request data directly from them, or verify and complete questionable or incomplete data. Inter-institutional networks for prevention of and attention for intra-familial violence were strengthened. This involved training in legal aspects of domestic violence, regular assessments of progress toward program objectives, and a review of updated competencies and qualifications of the individuals involved. A testing process was used that objectively assessed staff knowledge of flow charts to screen child abuse cases, procedural manuals, and technical documents outlining agency competencies and integral involvement in the care process. The project developed consensual inter-institutional flow charts for the attention of domestic violence cases and group exercises for the development of internal flow charts intended to clarify the process. In each municipality the project team identified local procedures regularly employed for the prevention and early detection of child abuse. This involved providing encouragement, support, and any needed consultation in developing prevention activities identified by teachers, parents, and health or protection personnel. The promotion of children's rights, humane childrearing practices, and assertive correction and discipline as a substitute for corporal punishment were emphasized. To provide for ongoing educational efforts the team identified a base of core facilitators and trained them in the early detection of cases. the project team made concerted efforts for sensitizing and training welfare and administrative teams from the network of health service providers handling cases of domestic violence. They did this in agreement with the guidelines by the Colombian Ministry of Health for Minors and Battered Women, which provides the framework for child and violence problems, among which domestic violence is included. Seventh, the project provided support for the development of public policy on children, and for giving greater attention to child abuse prevention issues. This strategy promoted, within the Social Policy Councils of the municipalities, the elaboration of public action proposals aimed at influencing the social policies of the municipality. These bodies have the legal mandate to design and recommend social policy guidelines to their mayors. In line with the development of prevention programs described in item five, the project tried to inform and educate the general community. As the overall process intended to strengthen local governance and relations with citizenry, the team project developed an indicator for the number of complaints of family violence reported by community members to authorities responsible for investigation and intervention. | In Cerrito and Candelaria, the process failed due to absence of political will. | | | A total of 6 893 cases were observed using 2004 and 2005 surveillance system data. The magnitude, characteristics and circumstances of cases of domestic violence were recorded. Representatives from nearly all of the participating institutions in the 21 municipalities reported improved integration and coordination of services in the quantity and quality of contacts made. The program administrators also noted greater adherence to protocol among participant institutions in inter-institutional networks, such as the Childhood and Family Sub-committees, Child Abuse and Domestic Violence Prevention Networks, and Social Policy Councils. An increase in the number of cases registered by source information institutions was observed in all phases of the implementation process for the 21 municipalities. Tuluá (185 000 inhabitants) is the most striking example: during 2002, the municipality documented 192 cases of domestic violence. In 2003, the total number was 394, while for 2004 the number soared to 1 059.16 This represents more than a five-fold increase in reported cases over a three-year period. The reliability and validity of the data recorded by each municipal surveillance system improved. Better coordination coupled with reporting of higher technical quality resulted in more comprehensive, valid and pertinent information. | | As a result of the project, all the observatories currently use geo-referenced systems that allow them to locate and intervene in cases of violence. For example, during the implementation of the observatories, the number of reports of domestic violence increased in Tuluá, as shown by the maps identifying cases. In Tuluá, as a result of the mapping, local authorities created new centers for the attention of those affected by domestic violence, including extended hours of attention up to 24 hours and during weekends. |
| Zolotor, 2009 | Colombia |  | To develop a child victimization survey among a diverse group of child protection experts and examine the performance of the instrument through a set of international pilot studies. | Children and institutions | Psychometric analysis of IPSCAN screening tool for children aged 11-18 (N=110 participants in Colombia, as well as three other countries outside of LAC) | | Screening questions about each type of victimization were designed to map to categories of assault or child maltreatment conventionally used in legal, research, and programmatic settings. These include physical abuse, physical discipline, sexual abuse, neglect, and psychological abuse for the home module. Institutional categories include sexual assault, physical assault, and psychological victimization. All questions are asked of the respondent with reference to the past year. Children are given the response options of “many times,” “sometimes,” “never” and “not in the past year but this has happened.” If children respond affirmatively, they are asked to identify the perpetrator as “adult,” “another child or adolescent” or “both.” Questions related to sexual abuse were originally followed by the response options of “more than 2 times,” and “once or twice.” | Study limitations included: no construct validity or other psychometric analyses; small, convenience samples. Limitation of screening tool: does not provide guidance on linkage to services | | | Screening tool identified physical and sexual violence in the home and institution among Colombian participants. Reliability: With the exception of the violence exposure scale (alpha = .69) which has a fair alpha, all other scales have alpha coefficients in the good to very good range (.72–.86). | | Study combined results from Colombia with 3 other non-LAC countries. |
| Agusti, 2012 | Guatemala | 2007-09 | To implement a comprehensive care program for sexual violence victims in six municipalities of Escuintla. | N=164 survivors (M&F, inc. children) | Observational; | | Comprehensive health services for adult and child survivors of SV to provide Health (inc. psych, RH, etc.); Prevention; Active case finding; collaboration with MSPAS; included case reporting and network of providers addressing GBV | Challenges related to under-reporting/under-utilization of health facilities by survivors; low reporting of VAC on the part of providers | | | Informed advocacy and policy for protocol on sexual violence | | Focuses on presenting types, frequencies, and perpetrators of sexual violence, less on the access to care/linkage to services |
| Ramos 2014 | Mexico | NR | To evaluate the acquisition, consolidation of the knowledge, and the modification of the attitudes associated with violence after participating in the Better Without Violence intervention. | Health professionals (N=132; 60% psychologists | Pre/post evaluation following training session | | Educational intervention with Health Professionals and Youth. Based on WHO model, "Skills for Health" and attempts to reduce violence against children and support health professionals. Covers 5 themes: What is violence, Violence in families, Violence among friends or romantic partners, Violence in Public. |  | | | Improvements in knowledge about violence and effects; no significant change in attitudes about violence | | Immediate pre-post study; no evaluation of how this translated into practice |
| Streuli, 2013 | Peru | 2008 | To implement the *Juntos* project for the prevention of VAC and other social health determinants. | Children & caregivers | Multistage sample conducted in Ayacucho. Participants included 49 children, aged 6-14yrs (31 Juntos and 18 non-juntos), and 60 adults (19 juntos and 11 non-juntos). Qualitative research methods: individual interviews, group discussions, well-being and community mapping, home- and school-based semi-structured observations and child-led photography | | *Juntos:* Conditional Cash transfer program aimed at poverty reduction. These programs offer cash to ‘poor’ people, with conditions requiring families to invest in their children through greater participation in education and health service | At the same time, however, there are limitations to these benefits, not least because Juntos cannot guarantee the quality and usefulness of government services that it obliges recipients to utilize. Given that a certain amount of stigmatization seems to accompany recipient children and families, one might also ask questions about effects on other aspects of children’s well-being and development, such as individual self-esteem, gender-based notions and practices, cultural identity and relationships within the community. | | | Has beneficial effects on certain aspects of children’s well-being, such as physical health, nutrition and schooling. | | Not peer reviewed |
| Jones & Marquez, 2014 | Peru | 2013 | To implement the *Juntos* project for the prevention of VAC and other social health determinants. | Children & caregivers | Not a study, Review | | *Juntos* Cash Transfer program: Provides cash support on the condition that children remain engaged in school, etc. ensures that all beneficiaries have civic identity documents, without which they cannot access basic social and protection services for themselves and their families. Complementary awareness- raising initiatives on intra-household violence and child protection issues have been integrated at the community level to varying degrees. In general, caregivers are expected to attend regular awareness-raising sessions on topics ranging from parenting to household management and children’s legal entitlements. | Provides social worker but does not link to child protection agency (DEMUNA) at the district level | | | Reportedly closed the poverty gap (by 5%) and reduced poverty severity (by 6%), while also contributing to a 28% increase in per capita household income among beneficiaries. Increased enrolment in service utilization and diet, modest improvements in school attendance, children with special needs in greater school attendance, reduction in child labor (10x less) | | Review of study; no study description |
| Reid , SD (2014) | Trinidad & Tobago | NR | To evaluate the Breaking the Silence Intervention (BTS) | Targeted three communities consisting of 13 villages in coastal and urban areas of Trinidad and Tobago (N=1,236 participants in BTS activities) | Implemented the BTS intervention, with pre-post design (four months between baseline and preliminary follow-up) | Breaking the Silence Gender and Community Empowerment Model. Used a community-based approach to develop final model. Activities included education, skills building, and service provision—that reflect the general needs identified by all three communities that were involved in the model design. All of the model activities introduced notions of gender and gender ideologies and how these ideologies affect differential risks, attitudes, and treatment approaches related to VAC. Educational activities aimed to (a) teach people about healthy relationships and to identify potentially abusive situations; (b) promote gender awareness and sensitivity to male and female participants; (c) inform about protective policies, services, and actions related to VAC; (d) educate about the relationship between VAC and HIV; and (e) motivate individuals to work toward changing the social structures and norms that support the occurrence of VAC. The skills building activities empowered adults and youth in the community to intervene directly or indirectly in the prevention of and response to VAC and the negative consequences associated with those experiences. (Tables 1 and 2 of publication). Service provision: With the assistance of trained professionals, community members who participated in the skills-building activities were involved in service provision. Services included community parenting support groups and groups for the empowerment of women and women survivors of VAC. Through family health fairs, local and national service providers also offered health screens, wellness workshops, and inter-active learning experiences addressing sexual health and condom use, family relationships and violence, and sexual abuse. | | Recognized challenges and delays related to broad inclusion of stakeholders - while provides the benefit of collective ownership, does take a long time and can lead to disagreements | | Increased knowledge, capacity building, and motivation to act in response to VAC. In each community, community members were motivated to establish and sustain support services to identify and help victims of VAC. One community established a parent support group, and the second established two support groups for VAC survivors. Of 1,236 participants in BTS activities, all reported a clearer understanding of VAC and had increased awareness of available community resources. Seventy-four percent of 221 participants (60% female) in a random sample of 10 education and skills-building workshops had an increase in knowledge of VAC, and 82% of 317 teachers reported increased knowledge of accurate definitions of VAC, the presenting signs, and ability to identify VAC. Eighty-two percent of teachers also reported an improved ability to build assertiveness in young persons’ sexual decision making and to talk about sexual issues in a youth-friendly manner. Seventy-four percent of 651 participants (except teachers) and 50% of teachers had greater understanding of gender ideologies and the relevance to VAC. Among 303 youth attending primary and secondary schools, 89% had greater knowledge/ understanding of VAC, gender ideologies, and the link between VAC and HIV. | | | Preliminary results are discussed |
